# Single-cell RNA sequencing of human kidney

**DOI:** 10.1038/s41597-019-0351-8

**Published:** 2020-01-02

**Authors:** Jinling Liao, Zhenyuan Yu, Yang Chen, Mengying Bao, Chunlin Zou, Haiying Zhang, Deyun Liu, Tianyu Li, Qingyun Zhang, Jiaping Li, Jiwen Cheng, Zengnan Mo

**Affiliations:** 1 Center for Genomic and Personalized Medicine, Guangxi Medical University, 530021 Nanning, Guangxi China; 2 Guangxi collaborative innovation center for genomic and personalized medicine, 530021 Nanning, Guangxi China; 3 Guangxi key laboratory for genomic and personalized medicine, Guangxi key laboratory of colleges and universities, 530021 Nanning, Guangxi China; 4 Institute of Urology and Nephrology, The First Affiliated Hospital of Guangxi Medical University, 530021 Nanning, Guangxi China; 5 Department of Urology, The First Affiliated Hospital of Guangxi Medical University, 530021 Nanning, Guangxi China; 6 Key Laboratory of Longevity and Aging-related Diseases(Guangxi Medical University), Ministry of Education, Nanning, Guangxi 530021 P.R. China; 7 Center for Translational Medicine & School of Preclinical Medicine, Guangxi Medical University, Nanning, Guangxi 530021 P.R. China; 8 Department of Urology, Affiliated Tumour Hospital of Guangxi Medical University, 530021 Nanning, Guangxi China; 9 Department of Cardiology, The First Affiliated Hospital of Guangxi Medical University, 530021 Nanning, Guangxi China; 10 Guangxi Key Laboratory of Precision Medicine in Cardio-cerebrovascular Diseases Control and Prevention, 530021 Nanning, Guangxi China; 11 Guangxi Clinical Research Center for Cardio-cerebrovascular Diseases, 530021 Nanning, Guangxi China

**Keywords:** Kidney, Cell biology, RNA sequencing

## Abstract

A comprehensive cellular anatomy of normal human kidney is crucial to address the cellular origins of renal disease and renal cancer. Some kidney diseases may be cell type-specific, especially renal tubular cells. To investigate the classification and transcriptomic information of the human kidney, we rapidly obtained a single-cell suspension of the kidney and conducted single-cell RNA sequencing (scRNA-seq). Here, we present the scRNA-seq data of 23,366 high-quality cells from the kidneys of three human donors. In this dataset, we show 10 clusters of normal human renal cells. Due to the high quality of single-cell transcriptomic information, proximal tubule (PT) cells were classified into three subtypes and collecting ducts cells into two subtypes. Collectively, our data provide a reliable reference for studies on renal cell biology and kidney disease.

## Abstract (summary)

**Table:** 

Measurement(s)	transcriptome • RNA
Technology Type(s)	RNA sequencing
Factor Type(s)	type of kidney cell
Sample Characteristic - Organism	Homo sapiens

Machine-accessible metadata file describing the reported data: 10.6084/m9.figshare.11365796

## Background & Summary

The kidney is a highly complex organ with many different functions, and consists of several functionally and anatomically discrete segments^1^. The glomerulus and renal tubules are important components of the nephron. The functional complexity of these structures appears to be associated with different cell types. Along with the glomerular endothelial cells, podocytes synthesize the glomerular basement membrane, which is the final filtration barrier, representing an important seal that prevents the loss of proteins into the urine^2^. Parietal epithelial cells (PECs) are another common glomerular cell type that might contribute to glomerulosclerosis, crescent and pseudocrescent formation^3^. The proximal tubule (PT) plays an important role in regulating systemic acid-base balance by controlling Na^+^-H^+^ and HCO_3_^−^ transport, while the distal convoluted tubule is more involved in electrolyte transport^4–6^. In previous studies, researchers have performed bulk RNA sequencing of different components of the kidney, providing a reference for understanding the transcriptome of different segments^7–9^. However, bulk RNA sequencing cannot reflect the transcriptome at the single-cell level, but only the overall average RNA expression.

With the development of next-generation sequencing, high-throughput single-cell analysis^10^ and the Human Cell Atlas^11^, scRNA-seq of the kidney became feasible. *Park, J et al*.^1^ characterised 57,979 cells from healthy mouse kidneys using unbiased single-cell RNA sequencing and revealed potential cellular targets of kidney disease. Later, *Young, M. D et al*.^12^ studied 72,501 single-cell transcriptomes of human renal carcinomas and normal tissue from foetal, paediatric and adult kidneys. These studies provided transcriptome maps of mouse and human kidneys. At the same time, it was reported that some renal diseases may be cell type-specific. For example, chronic kidney disease (CKD) is associated with PT cells^13^. Thus, PT cells have attracted extensive attention. However, the number of human PT cells obtained in the abovementioned studies was relatively small, i.e. approximately 1,000 high-quality PT cells which highly expressed marker genes. It is difficult to classify a subpopulation of PT cells. Moreover, in previous studies^1,13^ that considered kidney disease associated with specific cell types, results were obtained based on mouse kidney transcriptome data.

**Fig. 1:**
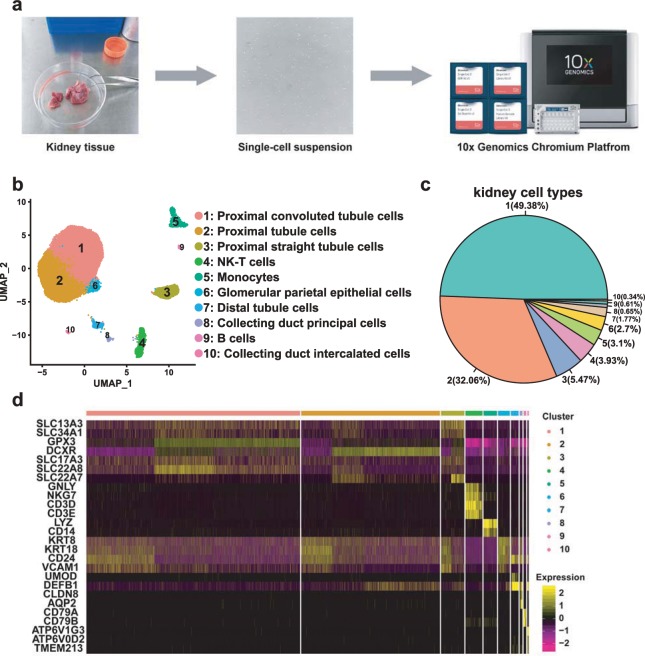
scRNA-seq reveals the cell populations of the human kidney. (**a**) Overview of the scRNA-seq process using human kidney tissue samples. (**b**) Uniform manifold approximation and projection (UMAP) plot showing the unbiased classification of renal cells. (**c**) Pie chart showed the proportion of each kidney cell type. (**d**) Heat map showing the marker genes of each cluster, highlighting the selected marker genes for each cluster.

To address this problem, we set out to obtain a single-cell suspension of the human kidney and performed scRNA-seq with a high throughput droplet-mediated scRNA-seq platform (10x Genomics Chromium^14^, Fig. 1a). We obtained a single-cell transcriptome dataset of 23,366 high-quality human kidney cells from three donors (kidneys 1, 2 and 3), including 20,308 PT cells. Considering the important role of PT cells in renal disease, this considerable individual cell transcriptome information may validate previously reported susceptibility genes for kidney disease. In addition, monogenic disease genes and complex trait genes identified by genome-wide association study (GWAS) may be associated with precise cell types. With the unbiased classification of cells, we can discover new genes with specific expression in some cell types. Taken together, the generated data provide more abundant transcriptomic information on renal tubular cells, representing an important reference for the accurate classification of renal tubular cells and the study of the relationship between renal tubular cells and diseases.

## Methods

We present an overview of the kidney scRNA-seq method. The whole process included the acquisition of human kidney tissue, the preparation of a single-cell suspension and 10x Genomics sample processing (Fig. 1a).

### Ethical approval

We received approval from the Institution Review Board (IRB) from the First Affiliated Hospital of Guangxi Medical University, and signed informed consent was obtained from all patients.

### Human kidney tissue procurement and isolation

Fresh human kidney samples (Supplementary Table S1) were collected at the First Affiliated Hospital of Guangxi Medical University and Affiliated Tumour Hospital of Guangxi Medical University. Two out of the three samples were obtained from patients undergoing radical nephrectomy, and the remaining sample was from a patient undergoing radical nephroureterectomy. Normal kidney tissues were obtained at least 2 cm away from tumour tissue.

First, fresh samples were taken from the operating room and placed in a solution containing Hank’s balanced salt solution (HBSS; WISENT, 311-512-CL) and 1% Antibiotic-Antimycotic (Gibco, 15240062) on ice, which was transported to the laboratory within 20 minutes. Then, 0.5–1 g full-thickness sections of kidney tissue were cut lengthwise using surgical scissors. Subsequently, the tissue was washed twice with cold Dulbecco’s phosphate-buffered saline (DPBS; WISENT, 311-425-CL). We placed the tissue on a stainless steel cell filter, crushed the tissue with the plunger of a syringe and washed it with DPBS. We added flushing fluid to a centrifuge tube, and collected the kidney fragments into the centrifuge tube, which was then spun at 350 *g* for 5 min at 4 °C; we then repeated this step. After discarding the supernatant, we used TrypLE™ Express Enzyme (1X, Gibco, 12605010) to further digest the sticky clumps of cells for 5–10 min at 37 °C, then terminated digestion using Dulbecco’s modified eagle medium (DMEM; WISENT, 319-006-CL) containing 10% fatal bovine serun (FBS; Gibco, 10099141). The digested cells were centrifuged at 350 g for 5 min at 4 °C. After discarding the supernatant, the cells were resuspended in 5 ml of DPBS and filtered through a 100 μm cell strainer. Next, we removed red blood cells using 1X RBC lysis buffer (10X diluted to 1X,BioLegend, B250015) for 5 min and centrifuged the cells at 300 g for 5 min at 4 °C. After discarding the supernatant, the cells were suspended in DPBS and centrifuged again. After discarding the supernatant, the cells were resuspended in cold DPBS and passed through a 40 μm cell strainer. Live cells were counted using trypan blue (0.4%, Gibco, 420301) staining. If the cell viability was above 80%, we perform 10x Genomics sample processing.

### 10x Genomics sample processing and cDNA library preparation

The 10x Genomics Chromium Single Cell 3′ Reagents Kit v2 user guide (https://support.10xgenomics.com/single-cell-gene-expression/index/doc/user-guide-chromium-single-cell-3-reagent-kits-user-guide-v2-chemistry) was used to prepare the single cell suspension. The single cell samples were passed through a 40 μm cell strainer and counted using a haemocytometer with trypan blue. Then, the appropriate volume of each sample was diluted to recover 10,000 kidney cells. Subsequently, the single cell suspension, Gel Beads and oils were added to the 10x Genomics single-cell A chip. We checked that there were no errors before running the assay. After droplet generation, samples were transferred into PCR tubes and we performed reverse transcription using a T100 Thermal Cycler (Bio-Rad). After reverse transcription, cDNA was recovered using a recovery agent, provided by 10x Genomics, followed by silane DynaBead clean-up as outlined in the user guide. Before clean-up using SPRIselect beads, we amplified the cDNA for 10 cycles. The cDNA concentration was detected by a Qubit2.0 fluorometer (Invitrogen). The kidney cDNA libraries were prepared referring to the Chromium Single Cell 3′ Reagent Kit v2 user guide.

### Single-cell RNA-seq details and preliminary results

Samples were sequenced by Hiseq Xten (Illumina, San Diego, CA, USA) with the following run parameters: read 1 for 150 cycles, read 2 for 150 cycles, index for 14 cycles. Preliminary sequencing results (bcl files) were converted to FASTQ files with CellRanger (version 3.0, https://support.10xgenomics.com/single-cell-gene-expression/software/pipelines/latest/what-is-cell-ranger). We followed the 10x Genomics standard seq protocol by trimming the barcode and unique molecular identifier (UMI) end to 26 bp, and the mRNA end to 98 bp. Then, the FASTQ files were aligned to the human genome reference sequence GRCh38. Subsequently, we applied CellRanger for preliminary data analysis and generated a file that contained a barcode table, a gene table and a gene expression matrix. We carried out preliminary quality control (QC) on the FASTQ files to ensure high quality scRNA-seq data. We also made a comparison between three different methods (Cell Ranger V2.1 or 2.2 with 150 bp × 2, Cell Ranger V3.0 with 150 bp × 2, Cell Ranger V3.0 with trimming the FASTQ data to 26 bp × 98 bp). We found that more single cells were actually identified using Cellranger V3.0 compared with Cellranger V2.0 or 2.1 (Tables 1 and 2). At the same time, we obtained some basic information about sequencing by a website, such as the number of cells, the median number of detected genes, sequencing saturation and sequencing depth (Table 2). The strategy of using CellRanger V3.0 and trimming the FASTQ data to 26 bp × 98 bp was used to pre-process the scRNA-seq data and perform downstream analysis.

**Table 1:** Detailed QC of FASTQ files.

Sample ID	Cellranger version and seq strategy	Q30 Bases in Barcode	Q30 Bases in RNA Read	Q30 Bases in UMI	Reads Mapped Confidently to Genome	Reads Mapped Confidently to Exonic Regions	Reads Mapped Confidently to Transcriptome
kidney1	Cellranger 2.1, 150 bp × 2	95.7%	89.6%	95.1%	92.8%	82.8%	79.4%
kidney1	Cellranger 3.0, 150 bp × 2	95.7%	86.6%	95.1%	91.3%	81%	76.5%
kidney1	Cellranger 3.0, 26 bp × 98 bp, 10x seq protocol	95.7%	89.6%	95.1%	92.9%	82.9%	79.3%
kidney2	Cellranger 2.2, 150 bp × 2	98.3%	94.1%	98.4%	96.1%	90.3%	85.6%
kidney2	Cellranger 3.0, 150 bp × 2	98.3%	94.2%	98.5%	96.1%	90.1%	85%
kidney2	Cellranger 3.0, 26 bp × 98 bp, 10x seq protocol	98.3%	96.9%	98.5%	87.4%	83.3%	79.6%
kidney3	Cellranger 2.2, 150 bp × 2	98.3%	93.7%	98.4%	96.1%	87.9%	82.8%
kidney3	Cellranger 3.0, 150 bp × 2	98.3%	93.7%	98.4%	96.3%	88%	82.6%
kidney3	Cellranger 3.0, 26 bp × 98 bp, 10x seq protocol	98.3%	94.6%	98.4%	96.5%	88.5%	83.2%

**Table 2:** Information and sequencing statistics of three samples and comparison between different sequencing strategies. NA, after pre-processing the data, some of them didn’t use for downstream analysis.

Sample ID	Cellranger version and seq strategy	Number of cells	Median number of detected genes	Sequencing saturation (%)	Mean reads per cell	Number of cells post filtering
kidney1	Cellranger 2.1, 150 bp × 2	8,091	856	85.9	42,485	NA.
Kidney1	Cellranger 3.0, 150 bp × 2	8,161	825	85.5	42,121	NA.
Kidney1	Cellranger 3.0, 26 bp × 98 bp, 10X seq protocol	8,164	837	85.7	42,105	7,221
kidney2	Cellranger 2.2, 150 bp × 2	6,472	960	85.6	37,763	NA.
kidney2	Cellranger 3.0, 150 bp × 2	6,493	910	80.2%	25,539	NA.
kidney2	Cellranger 3.0, 26 bp × 98 bp, 10X seq protocol	6,499	887	79.9%	25,515	5,543
kidney3	Cellranger 2.2, 150 bp × 2	10,705	692	85.7	21,064	NA.
Kidney3	Cellranger 3.0, 150 bp × 2	10,741	683	85.6	20,993	NA.
Kidney3	Cellranger 3.0, 26 bp × 98 bp, 10X seq protocol	10,741	684	85.6	20,993	10,602

### Using Seurat for quality control (QC) and data second analysis after mitigating the batch effect

**Fig. 2:**
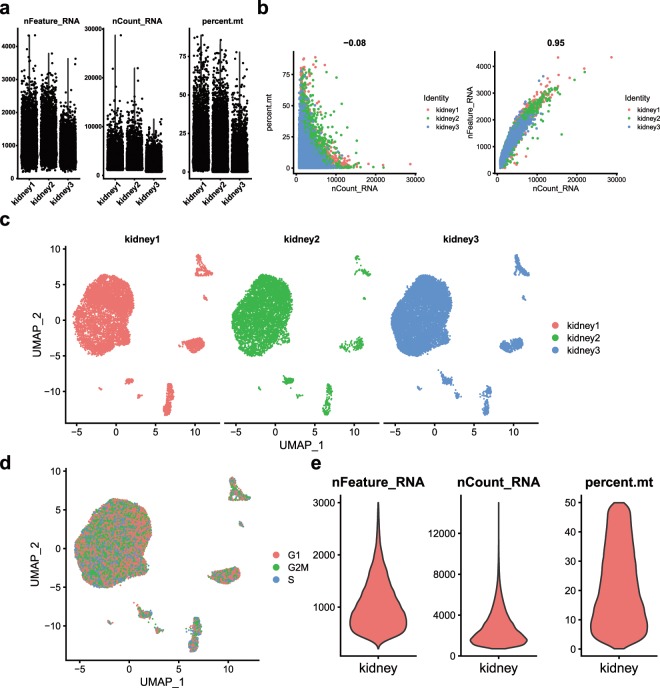
Quality control (QC) of human kidney single cell data. (**a**) Scatterplot illustrating the number of genes, unique molecular identifiers (UMIs) and the percentage of mitochondrial genes in each cell of three kidney samples. (**b**) The relationship between the percentage of mitochondrial genes and the mRNA reads, together with the relationship between the amount of mRNA and the reads of mRNA. (**c**) We detected the batch effect between three different kidney samples. (**d**) UMAP plot showing the cell cycle status of each cell. (**e**) Violin plot illustrating the number of genes, unique molecular identifiers (UMIs) and the percentage of mitochondrial genes in previous kidney single-cell data from GSE107585^1^.

We used the R (version 3.5.2, https://www.r-project.org/) and Seurat^15,16^ R package (version 3.1, https://satijalab.org/seurat/). We used the MergeSeurat function to merged the three kidney data sets. The filter criteria of cells were determined after reference to previous studies^1,12^. According to the median number of genes and the percentage of mitochondrial genes in the kidney samples (Fig. 2a), cells with <200 and >2,500 genes (potential cell duplets) and a mitochondrial gene percentage of >30% were filtered. After QC, 23,366 high quality kidney cells were obtained. The relationship between the percentage of mitochondrial genes and the mRNA reads were detected and visualised, together with the relationship between the number of mRNAs and the reads of mRNA (Fig. 2b).

After data normalisation, all highly variable genes in single cells were identified after controlling for the relationship between average expression and dispersion. All variable genes (n = 16471) were used in the downstream analysis, i.e. principal-component analysis (PCA).

Since this data came from three different samples, in order to avoid the batch effect affecting downstream analysis, we adopted a strategy of mitigating the batch effect, called ‘Harmony’. The R package Harmony focuses on scalable integration of scRNA-seq data for batch correction and meta analysis^17^ (version 0.99.9, https://github.com/immunogenomics/Harmony). To ensure the reliability of Harmony, we also applied another approach based on mutual nearest neighbours (MNN)^18^, which was implemented in the R package scran^19^ (the “fastMNN” function). We found that these two methods of eliminating batch effects produced similar results (Fig. 2c, Supplementary Fig. S1a). However, Harmony may be slightly better than fastMNN for our data. Harmony identified collecting duct intercalated cells with the same reduced resolution, but fastMNN could not (Fig. 1b, Supplementary Fig. S1b,c). Therefore, we used Harmony to eliminate batch effects and continued downstream analysis.

Subsequently, we used PCA with variable genes as the input and identified significant principal components (PCs) based on the jackStraw function. Twenty PCs were selected as the input for uniform manifold approximation and projection (UMAP) and t-distributed stochastic neighbour embedding (tSNE) when statistically significant. We detected the batch effect between three different kidney samples (Fig. 2c and Supplementary Fig. S2a). With a resolution of 0.25, cells were clustered by the FindClusters function and classified into 10 different cell types. Next, we used the FindAllMarkers function to find differentially expressed genes between each type of cell (Supplementary Table S2).

### Cell cycle analysis

Cell cycle analysis was performed by using the Seurat program. We used a previously defined core set of 43 G1/S and 54 G2/M cell cycle genes^20^. Cells were classified by the maximal average expression (‘cycle score’) in these two gene sets. When the cycle scores of G1/S and G2/M were both less than 2, we considered these cells to be non-cycling. Otherwise, we considered cells to be proliferative. After cell cycle analysis, no bias induced by cell cycle genes was observed (Fig. 2d and Supplementary Fig. S2b).

### Cell type markers

Cell type assignment was performed based on the marker genes reported in previous studies^1,3,7–9,12,21–23^ (Online-only Table 1).

### Reconstructing PT cells differentiation trajectories by Monocle2

PT cells fate decisions and pseudotime trajectories were reconstructed by the Monocle2^24^ R package (version 2.10.1, http://cole-trapnell-lab.github.io/monocle-release/). First of all, the three types of PT cells were selected by Seurat. The PT cell data, which included 20,308 PT cells, were imported into Monocle2. We used genes that were expressed in at least 10 cells and in greater than 5% of cells. Subsequently, we used thresholds on the cell local density (rho) and nearest distance (delta) to determine the number of clusters. Then, we performed differential gene expression analysis as before, but across all cell clusters. We used the top 1,000 most significantly differentially expressed genes as the set of ordering genes and performed dimension reduction and trajectory analysis. Once we established a trajectory, we used the differential GeneTest function to find genes that had an expression pattern that varied according to pseudotime.

## Data Records

All kidney sequencing data have been uploaded to the NCBI GEO database. It is possible to access these data through the project accession number GSE131685^25^. These data include barcodes.tsv, features.tsv and gene expression matrix (*.mtx) files. The raw data in the bam files have been deposited in the NCBI Sequence Read Archive (SRA) and are accessible through the project accession number SRP199294^26^.

## Technical Validation

Kidney specimens were collected fresh, dissected and digested into single cells from organ donors (two males and one female) aged 57–65 years (Supplementary Table S1, Methods). We used Seurat for QC, in which the number of genes, the number of UMI and the percentage of mitochondrial genes in each cell were calculated (Fig. 2a). We made a comparison with the previous kidney single-cell data from GSE107585^1^ on the number of genes detected per cell (Fig. 2e). We found that the median genes per cell were 941. This result was close to our scRNA-seq results. In general, the proportion of mitochondrial genes in the kidney cells was greater than in other organs, such as the liver, prostate, testis and peripheral blood mononuclear cell (PBMC)^1,12,23,27–29^. Since the proportion of mitochondrial genes reflects the state of cells, the exclusion criteria are controversial. Some researchers have suggested that kidney cells should discarded if their mitochondrial gene percentage is over 50%^1^, while other researchers remove any cells that have greater than 20% expression originating from mitochondrial genes^12^. In this study, we were conservative, in that cells with a mitochondrial gene percentage of >30% were filtered.

After QC, 23,366 high quality kidney cells were further analysed. We could identify 10 cell clusters that consisted of cells in the range of 79–11,539 cells per cluster (Fig. 1c). We visualised cell clustering using two different approaches (UMAP and tSNE), and the results were the same (Fig. 1b and Supplementary Fig. S2c). According to the marker genes (Online-only Table 1), we classified cells into clusters 1–10, corresponding to proximal convoluted tubule cells, proximal tubule cells, proximal straight tubule cells, NK-T cells, monocytes, glomerular parietal epithelial cells, distal tubule cells, collecting duct principal cells, B cells and collecting duct intercalated cells (Fig. 1d).

**Fig. 3:**
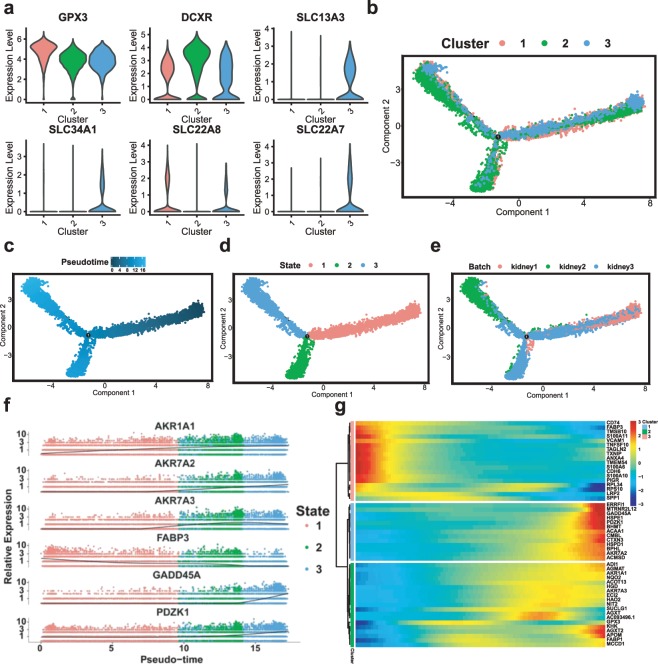
Subpopulations of PT cells and reconstructing the developmental trajectory of PT cells. (**a**) Violin plots representing the expression of marker genes in PT cells. Clusters 1, 2 and 3 refer to the proximal convoluted tubule, proximal tubule and proximal straight tubule, respectively. (**b**) Monocle2-generated pseudotemporal trajectory of three PT cell types (*n* = 20,308); imported Seurat data are coloured according to the cell name designation. (**c**) Pseudotime was coloured in a gradient from dark to light blue, and the start of pseudotime is dark. (**d**) The pseudotime trajectory was divided into three different states by Monocle2. (**e**) The trajectory showing the distribution of cells from three samples. (**f**) The top six genes influencing fate decisions are shown as line plots displayed as the expression level over pseudotime by Monocle2. (**g**) Heat map for clustering the top 50 genes that affected cell fate decisions. These 50 genes were divided into three clusters (cluster 1, cluster 2 and cluster 3), showing genes at the beginning stage, the transitory stage and the end stage of the developmental trajectory, respectively.

Our results show that PT cells were very abundant, with 20,308 PT cells. PT cells can be classified into three different clusters according to their markers, including the proximal convoluted tubule, proximal straight tubule and PT cells of no accurate classification (Figs. 1b and 3a, Online-only Table 1). Furthermore, we applied Monocle2 to perform pseudotime trajectories of all PT cells and showed the fate decisions between them (Fig. 3b–e). At the same time, we discovered the top six genes that influenced fate decisions (Fig. 3f). To show more detailed gene information, we presented the top 50 genes that affect fate decisions (Fig. 3g).

**Fig. 4:**
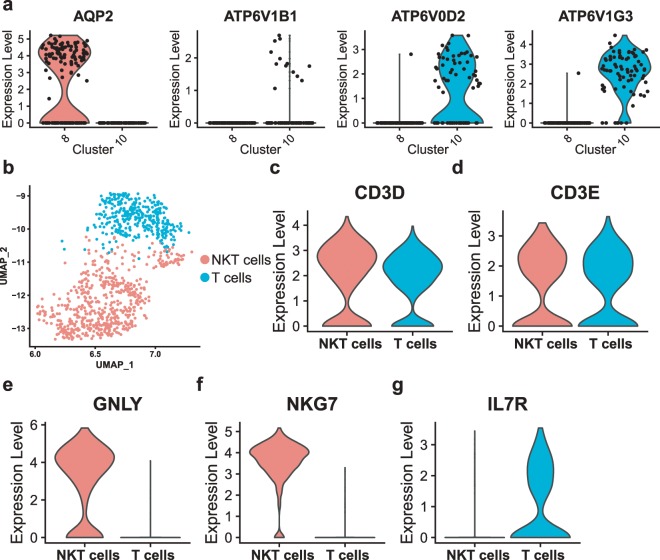
Detailed classification of collecting duct cells and NK-T cells by scRNA-seq. (**a**) Violin plot showing the expression of the collecting duct principal cell marker *AQP2* and the collecting duct intercalated cell markers *ATP6V1B1, ATP6V0D2* and *ATP6V1G3*. Clusters 8 and 10 are collecting duct principal cells and collecting duct intercalated cells, respectively. (**b**) UMAP plot showing the spatial location of NKT cells and T cells after dimensionality reduction. The red dots represent NKT cells and the green dots represent T cells. (**c**–**g**) Violin plots showing the expression of the NK cells markers *GNLY* and *NKG7*. Violin plots showing the expression of the T cell markers *CD3D, CD3E* and *IL7R*.

Initially the collecting duct was described as having a role only in water reabsorption, while in recent years the understanding of the function of the collecting duct has become greatly enhanced and has led to a new model for how the distal segments of the kidney tubule integrate salt and water reabsorption, potassium homeostasis and acid-base status^30^. Interestingly, our data also provide transcriptomic information for collecting duct cells (Fig. 1b, cluster 8, 10). We classified collecting duct cells into principal cells (cluster 8) and intercalated cells (cluster 10), according to marker expression (Fig. 4a, Online-only Table 1). Given that the three ‘healthy’ kidney samples were collected from renal cancer patients, we had to confirm their universal representativeness. Previous studies on human kidney scRNA-seq^12^ have provided us with many marker genes for proximal, distal and collecting tubule cells. We found that almost all the genes for PT cells were highly expressed in our PT cells (Supplementary Fig. S3a). Most of these genes for distal and collecting tubules cells were expressed in our data (Supplementary Fig. S3b). Thus, we consider these results to be reliable.

Finally, we present a method for the detailed classification of cell subsets. Initially, the parameters of 20 PCs and 0.25 resolution were selected to identify 10 cell types (Fig. 1b). We found that cluster 4 highly expressed marker genes of both NK cells and T cells, designated as NK-T cells (Fig. 1d, Supplementary Table S2). Interestingly, cluster 4 can be further classified into two subtypes (Fig. 4b). By modifying the parameters to 20 PCs and 0.8 resolution, we could accurately distinguish NKT cells (*CD3D*^+^*CD3E*^+^*GNLY*^+^*NKG7*^+^) and T cells (*CD3D*^+^*CD3E*^+^*IL7R*^+^) (Fig. 4c–g), which can be used for downstream analysis.

Taken together, we provide a transcriptomic map of human kidney cells that will help us to study renal cell biology and the relationship between cell types and diseases.

### Supplementary information

Supplementary Information

## Data Availability

The R code used in the analysis of the scRNA-seq data is available on GitHub (https://github.com/lessonskit/Single-cell-RNA-sequencing-of-human-kidney). This R code is also available at figshare^31^.

## Online-only Table

**Online-only Table 1:** Cell type assignment based on the marker genes reported in previous studies.

Gene	Marker of	Reference
*SLC13A3*	Proximal tubule	^7,9,12^
*SLC34A1*	Proximal tubule	^7,9,12^
*GPX3*	Proximal tubule	^8^
*DCXR*	Proximal tubule	^21^
*SLC22A8*	Proximal convoluted tubule	^7,9,12^
*SLC22A7*	Proximal straight tubule	^7,9,12^
*KRT8*	Glomerular parietal epithelial cells	^3^
*KRT18*	Glomerular parietal epithelial cells	^3^
*CD24*	Glomerular parietal epithelial cells	^3^
*VCAM1*	Glomerular parietal epithelial cells	^3^
*LYZ*	Monocytes	^23^
*CD14*	Monocytes	^23^
*GNLY*	NK cells	^23^
*NKG7*	NK cells	^23^
*CD3D*	T cells	^23^
*CD3E*	T cells	^23^
*IL7R*	T cells	^23^
*CD79A*	B cells	^23^
*CD79B*	B cells	^23^
*UMOD*	Distal tubule	^9^
*DEFB1*	Distal tubule, Collecting duct	^22^
*CLDN8*	Collecting duct	^7–9,12^
*AQP2*	Collecting duct principal cells	^1,7,9,12^
*ATP6V1G3*	Collecting duct intercalated cells	^1^
*ATP6V0D2*	Collecting duct intercalated cells	^9,12^
*TMEM213*	Collecting duct intercalated cells	^9^
