# Supplementary material for: Single-cell RNA sequencing of human kidney
Source: Sci Data. 2020 Jan 2;7:4. doi: 10.1038/s41597-019-0351-8 (PMC6940381; doi:10.1038/s41597-019-0351-8)
Supplement: Supplementary file 1 — Supplementary Information [file 41597_2019_351_MOESM1_ESM.pdf]

Supplementary Figure legends

Supplementary Figure S1 (Page 2) Comparing the results of fastMNN and Harmony in eliminating the batch effect. **a** After eliminating batch effects with fastMNN, we detected the batch effect between three different kidney samples. **b** After treating with fastMNN, cells were clustered by UMAP. **c** After treating with fastMNN, UMAP plot showed the cell cycle of each cell.

Supplementary Figure S2 (Page 3) Visualisation of cell clustering by tSNE. **a** tSNE plot showing the batch effect between three different kidney samples. **b** tSNE plot showing the cell cycle of each cell. **c** tSNE plot showing the unbiased classification of renal cells.

Supplementary Figure S3 (Page 4) Comparison between this study and kidney scRNA-seq data from previous studies. **a** Genes that were highly expressed in proximal tubule cells in a previous study were also expressed in this cell population in our study. **b** Most genes that were highly expressed in distal tubule cells and collecting duct cells in a previous study were also expressed in this cell population in our study.

Supplementary Table legends

Supplementary Table 1 (Page 5) Information of three different patients.

Supplementary Table 2 (Page 6) Differential expression genes (DEGs) of each cluster about human kidney cells.

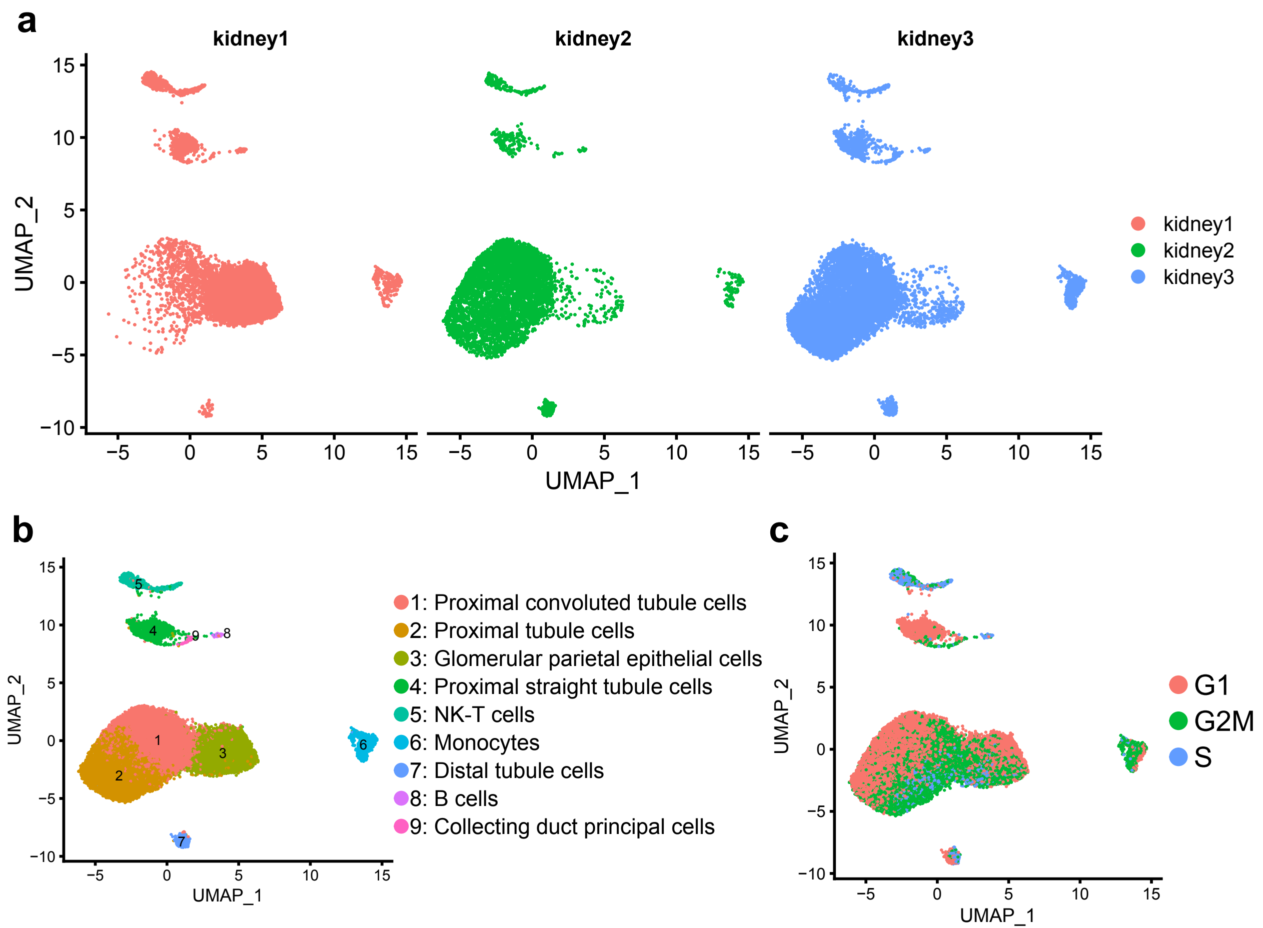
a
kidney1
kidney2
kidney3
UMAP_2
UMAP_1
kidney1
kidney2
kidney3
b
UMAP_2
UMAP_1
1: Proximal convoluted tubule cells
2: Proximal tubule cells
3: Glomerular parietal epithelial cells
4: Proximal straight tubule cells
5: NK-T cells
6: Monocytes
7: Distal tubule cells
8: B cells
9: Collecting duct principal cells
c
UMAP_2
UMAP_1
G1
G2M
S

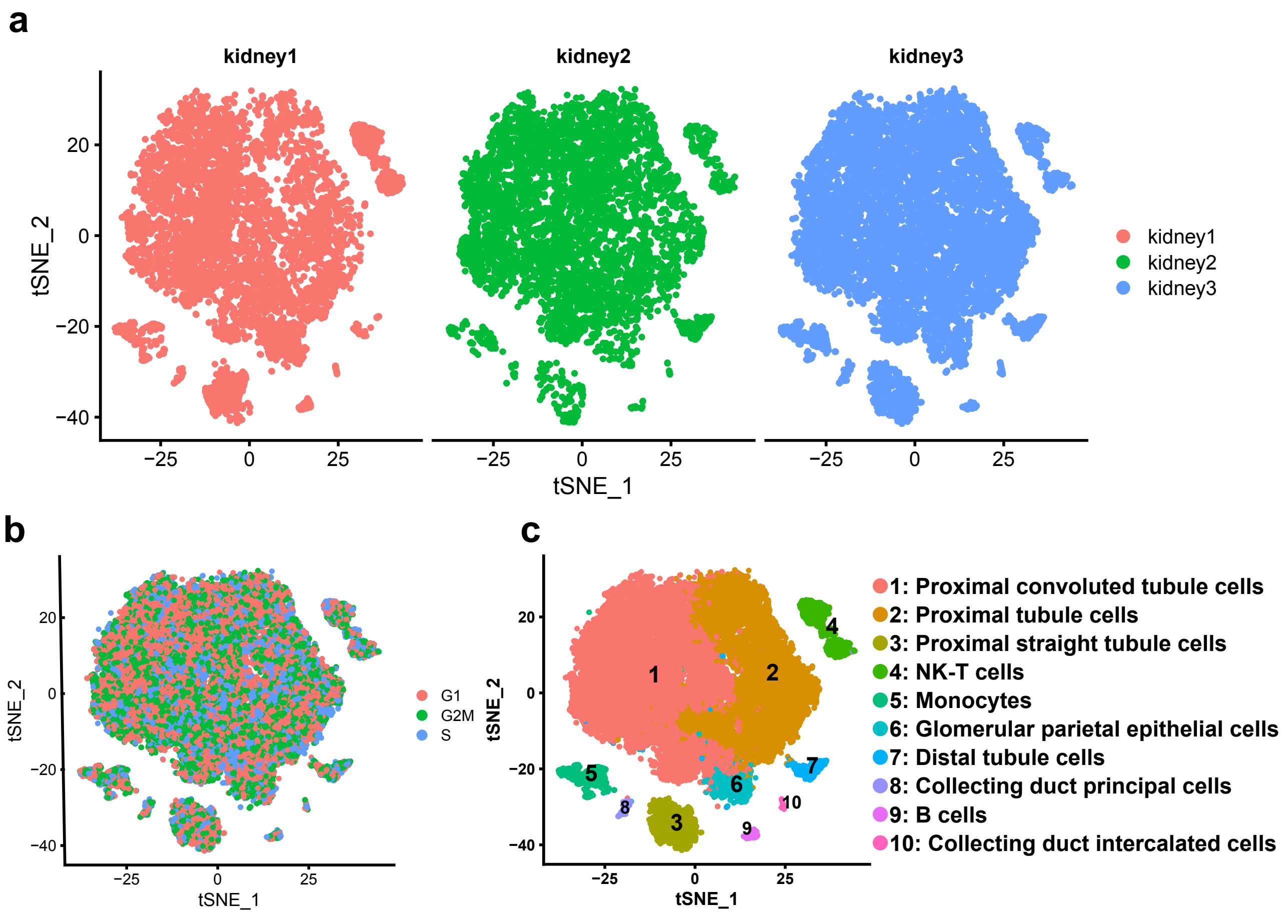
a
kidney1
kidney2
kidney3
tSNE_2
tSNE_1
20
0
−20
−40
−25
0
25
kidney1
kidney2
kidney3
b
tSNE_2
tSNE_1
G1
G2M
S
c
tSNE_2
tSNE_1
1: Proximal convoluted tubule cells
2: Proximal tubule cells
3: Proximal straight tubule cells
4: NK-T cells
5: Monocytes
6: Glomerular parietal epithelial cells
7: Distal tubule cells
8: Collecting duct principal cells
9: B cells
10: Collecting duct intercalated cells

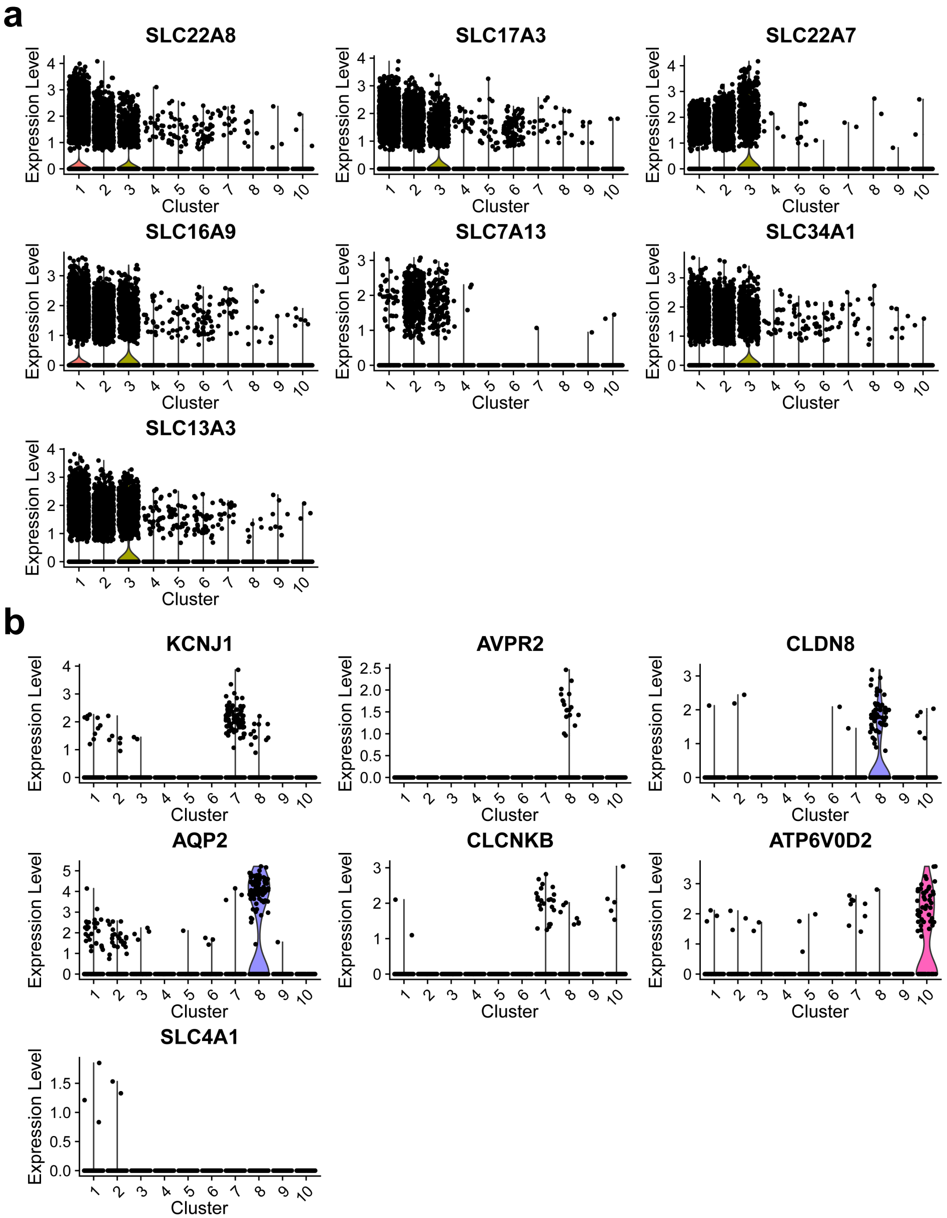
a
SLC22A8
SLC17A3
SLC22A7
SLC16A9
SLC7A13
SLC34A1
SLC13A3
b
KCNJ1
AVPR2
CLDN8
AQP2
CLCNKB
ATP6V0D2
SLC4A1
Expression Level
Cluster

Table S1 Information of three different patients.

ID	Hospital	Age/Sex	Ethnic	Histology	Smoker/Dringker	Height（cm）/Weight（kg）	Sample	Surgery Date
kidney1	The first affiliated hospital of Guangxi Medical University	57/male	The Mulao nationality	Papillary urothelial carcinoma of the renal pelvis	Y/Y	170/74	normal kidney	2018.4.16
kidney2	Affiliated tumor hospital of Guangxi Medical University	59/female	The Zhuang nationality	Clear renal cell carcinoma	N/N	152/47	normal kidney	2018.8.1
kidney3	Affiliated tumor hospital of Guangxi Medical University	65/male	The Han nationality	Clear renal cell carcinoma	Y/N	162/59	normal kidney	2018.8.2

Table S2 Differential expression genes (DEGs) of each cluster about human kidney cells.

p_val	avg_logFC	pct.1	pct.2	p_val_adj	cluster	gene
0	1.1133382	0.678	0.299	0	Proximal convoluted tubule cells	FABP1
0	1.09102681	0.995	0.889	0	Proximal convoluted tubule cells	GPX3
0	0.93289427	0.81	0.454	0	Proximal convoluted tubule cells	PCSK1N
0	0.91712919	0.314	0.079	0	Proximal convoluted tubule cells	FAM151A
0	0.90461562	0.347	0.11	0	Proximal convoluted tubule cells	SLC22A8
0	0.89613383	0.459	0.208	0	Proximal convoluted tubule cells	PRODH2
0	0.84730597	0.53	0.252	0	Proximal convoluted tubule cells	IGFBP7
0	0.78223775	0.375	0.16	0	Proximal convoluted tubule cells	PRAP1
0	0.78050296	0.694	0.442	0	Proximal convoluted tubule cells	UGT2B7
0	0.77930454	0.514	0.294	0	Proximal convoluted tubule cells	DPEP1
0	0.75902118	0.923	0.747	0	Proximal convoluted tubule cells	MT-ATP6
0	0.74101676	0.869	0.679	0	Proximal convoluted tubule cells	MT-ND1
0	0.73795918	0.965	0.789	0	Proximal convoluted tubule cells	APOE
0	0.73083161	0.96	0.827	0	Proximal convoluted tubule cells	MT-CO3
0	0.71097326	0.898	0.699	0	Proximal convoluted tubule cells	MT-ND3
0	0.70390041	0.372	0.169	0	Proximal convoluted tubule cells	SLC7A7
0	0.68908502	0.373	0.174	0	Proximal convoluted tubule cells	PAH
0	0.67582548	0.956	0.828	0	Proximal convoluted tubule cells	MT-CO1
0	0.6717499	0.799	0.56	0	Proximal convoluted tubule cells	SMIM24
0	0.64545047	0.932	0.778	0	Proximal convoluted tubule cells	MT-ND4
0	0.63232377	0.912	0.745	0	Proximal convoluted tubule cells	MT-ND2
0	0.63009999	0.908	0.738	0	Proximal convoluted tubule cells	MT-CYB
0	0.6123025	0.962	0.845	0	Proximal convoluted tubule cells	MT-CO2
0	0.55892361	0.914	0.718	0	Proximal convoluted tubule cells	CXCL14
0	0.54796713	0.659	0.476	0	Proximal convoluted tubule cells	SELENOP
0	0.52592385	0.72	0.524	0	Proximal convoluted tubule cells	MT-ND5
0	0.46731991	0.973	0.814	0	Proximal convoluted tubule cells	PDZK1IP1
0	0.36793116	0.899	0.789	0	Proximal convoluted tubule cells	RPS27L
7.00E-307	0.35716653	0.906	0.808	1.15E-302	Proximal convoluted tubule cells	DBI
7.40E-301	0.4043868	0.857	0.732	1.22E-296	Proximal convoluted tubule cells	ITM2B
3.23E-283	0.35566834	0.892	0.8	5.31E-279	Proximal convoluted tubule cells	NDUFB2
4.04E-283	0.76042808	0.393	0.195	6.66E-279	Proximal convoluted tubule cells	ALB
1.21E-280	0.35121279	0.984	0.887	2.00E-276	Proximal convoluted tubule cells	MT1X
1.47E-280	0.65576749	0.266	0.095	2.43E-276	Proximal convoluted tubule cells	THY1
6.10E-268	0.36606745	0.824	0.653	1.00E-263	Proximal convoluted tubule cells	NAT8
1.43E-260	0.44043145	0.726	0.57	2.36E-256	Proximal convoluted tubule cells	ASS1
1.00E-248	0.30207123	0.918	0.747	1.65E-244	Proximal convoluted tubule cells	ALDOB
3.08E-216	0.54291791	0.428	0.258	5.07E-212	Proximal convoluted tubule cells	CA2
2.11E-208	0.57591933	0.334	0.177	3.47E-204	Proximal convoluted tubule cells	PSAT1
8.89E-202	0.30534285	0.898	0.774	1.46E-197	Proximal convoluted tubule cells	GATM
2.19E-195	0.51113566	0.371	0.212	3.61E-191	Proximal convoluted tubule cells	CTSB
3.81E-189	0.34311988	0.829	0.749	6.27E-185	Proximal convoluted tubule cells	POLR2L
6.74E-183	0.30323931	0.8	0.672	1.11E-178	Proximal convoluted tubule cells	LGALS2
6.86E-176	0.41862081	0.683	0.532	1.13E-171	Proximal convoluted tubule cells	S100A1
6.86E-173	0.35333688	0.648	0.526	1.13E-168	Proximal convoluted tubule cells	NPC2
8.95E-173	0.31208079	0.895	0.781	1.47E-168	Proximal convoluted tubule cells	FXYD2
6.60E-168	0.35127943	0.655	0.52	1.09E-163	Proximal convoluted tubule cells	TMEM176A
3.83E-160	0.33622364	0.736	0.637	6.30E-156	Proximal convoluted tubule cells	ATOX1
1.47E-158	0.48477597	0.426	0.283	2.43E-154	Proximal convoluted tubule cells	MT-ND4L
9.25E-158	0.49346208	0.269	0.135	1.52E-153	Proximal convoluted tubule cells	NUPR1
3.99E-153	0.25268891	0.921	0.851	6.57E-149	Proximal convoluted tubule cells	UQCRQ
4.62E-149	0.34885822	0.671	0.528	7.61E-145	Proximal convoluted tubule cells	HPD
2.27E-132	0.34734407	0.734	0.616	3.73E-128	Proximal convoluted tubule cells	GSTA1
3.52E-127	0.46034885	0.319	0.192	5.80E-123	Proximal convoluted tubule cells	MCCD1
3.16E-122	0.44702362	0.326	0.207	5.20E-118	Proximal convoluted tubule cells	PTH1R
3.70E-101	0.33194402	0.549	0.449	6.10E-97	Proximal convoluted tubule cells	TMBIM6

1.22E-100	0.31600259	0.567	0.474	2.01E-96	Proximal convoluted tubule cells	FKBP2
3.13E-98	0.25453753	0.746	0.678	5.15E-94	Proximal convoluted tubule cells	ATP5IF1
1.40E-97	0.36523348	0.416	0.307	2.30E-93	Proximal convoluted tubule cells	TSPAN1
2.92E-96	0.27129363	0.549	0.433	4.82E-92	Proximal convoluted tubule cells	ATP1B1
3.93E-94	0.40345829	0.306	0.206	6.48E-90	Proximal convoluted tubule cells	PGRMC1
9.80E-94	0.37967125	0.667	0.604	1.61E-89	Proximal convoluted tubule cells	MDH1
1.14E-90	0.37465833	0.262	0.163	1.88E-86	Proximal convoluted tubule cells	SLC16A9
1.78E-87	0.40941784	0.271	0.174	2.93E-83	Proximal convoluted tubule cells	RARRES2
2.75E-87	0.36030058	0.433	0.339	4.54E-83	Proximal convoluted tubule cells	QDPR
2.64E-85	0.35284718	0.319	0.22	4.35E-81	Proximal convoluted tubule cells	GGH
5.23E-85	0.36341591	0.274	0.176	8.62E-81	Proximal convoluted tubule cells	SLC22A6
7.03E-77	0.28481775	0.546	0.475	1.16E-72	Proximal convoluted tubule cells	ATP6V0E1
1.00E-75	0.25338303	0.592	0.504	1.65E-71	Proximal convoluted tubule cells	TMEM176B
1.84E-74	0.26332585	0.491	0.388	3.03E-70	Proximal convoluted tubule cells	FABP3
3.03E-71	0.35794901	0.407	0.323	4.99E-67	Proximal convoluted tubule cells	CTSH
6.08E-68	0.25611064	0.555	0.476	1.00E-63	Proximal convoluted tubule cells	CD63
2.12E-67	0.27755805	0.434	0.344	3.49E-63	Proximal convoluted tubule cells	S100A13
2.67E-64	0.261708	0.533	0.47	4.40E-60	Proximal convoluted tubule cells	SNRPG
2.48E-63	0.32714735	0.32	0.238	4.08E-59	Proximal convoluted tubule cells	FCGRT
1.94E-62	0.28649914	0.439	0.366	3.19E-58	Proximal convoluted tubule cells	NENF
6.38E-59	0.26318519	0.473	0.395	1.05E-54	Proximal convoluted tubule cells	SH3YL1
6.91E-59	0.31369244	0.305	0.227	1.14E-54	Proximal convoluted tubule cells	KIAA1191
2.01E-58	0.33703241	0.269	0.193	3.31E-54	Proximal convoluted tubule cells	CTSA
1.01E-57	0.28390727	0.276	0.195	1.66E-53	Proximal convoluted tubule cells	LRP2
7.84E-56	0.33424921	0.283	0.21	1.29E-51	Proximal convoluted tubule cells	ALDH4A1
5.33E-55	0.33782243	0.326	0.252	8.77E-51	Proximal convoluted tubule cells	FAM107B
4.91E-44	0.25053774	0.304	0.233	8.08E-40	Proximal convoluted tubule cells	AQP1
8.68E-43	0.27090826	0.27	0.207	1.43E-38	Proximal convoluted tubule cells	ACOT7
5.94E-36	0.35959463	0.378	0.324	9.78E-32	Proximal convoluted tubule cells	PCP4
6.08E-33	0.25502903	0.27	0.215	1.00E-28	Proximal convoluted tubule cells	NAPSA
0	1.51340819	0.497	0.09	0	Proximal tubule cells	RBP4
0	1.30613413	0.422	0.071	0	Proximal tubule cells	AGXT
0	1.20966493	0.854	0.597	0	Proximal tubule cells	DCXR
0	1.1275391	0.361	0.063	0	Proximal tubule cells	AC093496.1
0	1.0631133	0.885	0.63	0	Proximal tubule cells	MIOX
0	0.90824912	0.501	0.194	0	Proximal tubule cells	HAO2
0	0.89804227	0.65	0.328	0	Proximal tubule cells	AKR7A3
0	0.88319926	0.61	0.308	0	Proximal tubule cells	GLYATL1
0	0.87222825	0.502	0.208	0	Proximal tubule cells	ACY3
0	0.84598806	0.84	0.551	0	Proximal tubule cells	BBOX1
0	0.84298187	0.292	0.057	0	Proximal tubule cells	GPT
0	0.83761656	0.276	0.057	0	Proximal tubule cells	GCAT
0	0.81975429	0.602	0.282	0	Proximal tubule cells	MPC1
0	0.81280969	0.691	0.401	0	Proximal tubule cells	ALDH6A1
0	0.80811686	0.529	0.293	0	Proximal tubule cells	SERPINA1
0	0.74853042	0.639	0.382	0	Proximal tubule cells	ALDH2
0	0.73113697	0.522	0.25	0	Proximal tubule cells	MSRB1
0	0.72777041	0.599	0.366	0	Proximal tubule cells	ADIRF
0	0.7116754	0.83	0.578	0	Proximal tubule cells	ACAT1
0	0.70225398	0.627	0.361	0	Proximal tubule cells	ACAA2
0	0.68475471	0.772	0.548	0	Proximal tubule cells	PCK1
0	0.68366711	0.267	0.074	0	Proximal tubule cells	LYG1
0	0.66468102	0.518	0.273	0	Proximal tubule cells	HGD
0	0.64617031	0.573	0.323	0	Proximal tubule cells	ACADM
0	0.63449456	0.625	0.371	0	Proximal tubule cells	NIT2
0	0.61557311	0.651	0.414	0	Proximal tubule cells	GRHPR
0	0.57798584	0.737	0.514	0	Proximal tubule cells	PEPD
0	0.5742302	0.772	0.564	0	Proximal tubule cells	RIDA

0	0.55116167	0.846	0.632	0	Proximal tubule cells	DAB2
0	0.52552034	0.747	0.531	0	Proximal tubule cells	GLYAT
0	0.51720389	0.973	0.863	0	Proximal tubule cells	PEBP1
0	0.51468623	0.743	0.532	0	Proximal tubule cells	C11orf54
0	0.5133821	0.993	0.959	0	Proximal tubule cells	GAPDH
0	0.4928283	0.895	0.735	0	Proximal tubule cells	ECHS1
0	0.40105302	0.956	0.858	0	Proximal tubule cells	TXN
0	0.3848988	0.925	0.806	0	Proximal tubule cells	PRDX1
0	0.32720806	0.999	0.962	0	Proximal tubule cells	MT1G
0	0.27931114	0.995	0.979	0	Proximal tubule cells	RPS6
0	0.27353406	0.999	0.995	0	Proximal tubule cells	EEF1A1
5.18E-300	0.60711163	0.326	0.127	8.54E-296	Proximal tubule cells	ASRGL1
6.36E-295	0.52866881	0.67	0.455	1.05E-290	Proximal tubule cells	CRYL1
5.41E-286	0.58759516	0.542	0.32	8.91E-282	Proximal tubule cells	PTGR1
5.93E-279	0.517918	0.616	0.392	9.77E-275	Proximal tubule cells	FBP1
4.82E-277	0.32416572	0.938	0.849	7.93E-273	Proximal tubule cells	GPX4
8.46E-276	0.59147108	0.455	0.239	1.39E-271	Proximal tubule cells	HIBCH
1.57E-274	0.31256954	0.951	0.887	2.58E-270	Proximal tubule cells	HINT1
4.09E-272	0.39922127	0.816	0.651	6.74E-268	Proximal tubule cells	DSTN
2.14E-267	0.54456955	0.595	0.387	3.52E-263	Proximal tubule cells	AKR7A2
4.21E-264	0.47254304	0.693	0.506	6.94E-260	Proximal tubule cells	SDHC
1.77E-260	0.60256037	0.616	0.411	2.91E-256	Proximal tubule cells	BHMT
3.93E-260	0.29982732	0.989	0.883	6.47E-256	Proximal tubule cells	MT1F
2.62E-258	0.32830437	0.937	0.82	4.31E-254	Proximal tubule cells	CYB5A
3.13E-258	0.30817717	0.944	0.841	5.16E-254	Proximal tubule cells	LDHB
5.37E-257	0.53898789	0.543	0.331	8.84E-253	Proximal tubule cells	ECI2
3.13E-253	0.54468067	0.289	0.113	5.15E-249	Proximal tubule cells	PTER
2.27E-251	0.46227598	0.723	0.543	3.74E-247	Proximal tubule cells	ETFB
7.81E-251	0.43651946	0.748	0.565	1.29E-246	Proximal tubule cells	AKR1A1
2.18E-249	0.56430339	0.458	0.253	3.60E-245	Proximal tubule cells	PHYH
8.11E-248	0.28408757	0.977	0.866	1.34E-243	Proximal tubule cells	CRYAB
4.18E-243	0.47748348	0.671	0.487	6.89E-239	Proximal tubule cells	ECH1
1.89E-242	0.43615613	0.739	0.579	3.12E-238	Proximal tubule cells	EEF2
3.74E-241	0.54025302	0.408	0.21	6.16E-237	Proximal tubule cells	BPHL
1.15E-240	0.47958915	0.643	0.443	1.89E-236	Proximal tubule cells	BDH2
1.13E-237	0.58303364	0.287	0.118	1.87E-233	Proximal tubule cells	WDR18
1.98E-235	0.3582388	0.892	0.737	3.26E-231	Proximal tubule cells	RBP5
3.79E-235	0.46577433	0.641	0.445	6.24E-231	Proximal tubule cells	HSPD1
1.32E-229	0.30211619	0.935	0.854	2.17E-225	Proximal tubule cells	SOD1
1.34E-220	0.54666699	0.308	0.138	2.20E-216	Proximal tubule cells	IDNK
9.05E-220	0.51196188	0.46	0.263	1.49E-215	Proximal tubule cells	ASPDH
6.20E-216	0.5268733	0.358	0.178	1.02E-211	Proximal tubule cells	AHCY
1.21E-214	0.25814379	0.991	0.88	1.99E-210	Proximal tubule cells	MT1H
3.32E-211	0.27678637	0.925	0.868	5.46E-207	Proximal tubule cells	NACA
1.21E-203	0.49422708	0.431	0.244	1.99E-199	Proximal tubule cells	ACMSD
7.19E-200	0.38901717	0.733	0.582	1.18E-195	Proximal tubule cells	ST13
5.90E-198	0.49353574	0.34	0.168	9.71E-194	Proximal tubule cells	SORD
4.14E-197	0.47782155	0.495	0.305	6.82E-193	Proximal tubule cells	MAOA
3.50E-193	0.37065906	0.774	0.638	5.77E-189	Proximal tubule cells	DDT
1.13E-192	0.36193546	0.749	0.609	1.87E-188	Proximal tubule cells	MGST3
1.18E-192	0.41828629	0.697	0.532	1.94E-188	Proximal tubule cells	PDZK1
4.05E-184	0.40851356	0.466	0.275	6.67E-180	Proximal tubule cells	GSTA2
2.72E-180	0.41204708	0.598	0.421	4.49E-176	Proximal tubule cells	BNIP3
1.27E-173	0.44276714	0.497	0.323	2.08E-169	Proximal tubule cells	CNDP2
3.00E-172	0.46862724	0.446	0.273	4.94E-168	Proximal tubule cells	SHMT1
9.69E-170	0.31359339	0.765	0.633	1.60E-165	Proximal tubule cells	ISCU
6.76E-166	0.47539832	0.28	0.136	1.11E-161	Proximal tubule cells	OAT
2.42E-165	0.44802412	0.33	0.173	3.99E-161	Proximal tubule cells	LINC01510

1.34E-161	0.44334777	0.338	0.182	2.20E-157	Proximal tubule cells	TCEA3
1.65E-158	0.37830311	0.639	0.483	2.72E-154	Proximal tubule cells	GHITM
5.61E-157	0.42207458	0.45	0.28	9.23E-153	Proximal tubule cells	CMBL
7.44E-155	0.4005563	0.556	0.392	1.23E-150	Proximal tubule cells	ACAA1
1.80E-151	0.34850387	0.686	0.544	2.96E-147	Proximal tubule cells	HSPA8
9.97E-148	0.33870348	0.677	0.531	1.64E-143	Proximal tubule cells	MPC2
3.82E-147	0.4143729	0.427	0.264	6.30E-143	Proximal tubule cells	ALDH1A1
3.88E-145	0.42949931	0.288	0.15	6.38E-141	Proximal tubule cells	NAPRT
4.78E-144	0.40103353	0.468	0.306	7.88E-140	Proximal tubule cells	SDHB
4.61E-143	0.38504697	0.517	0.356	7.60E-139	Proximal tubule cells	AK4
1.52E-141	0.39535764	0.481	0.318	2.51E-137	Proximal tubule cells	ACOT13
1.54E-139	0.30554894	0.722	0.582	2.54E-135	Proximal tubule cells	PRDX2
6.40E-138	0.38966145	0.396	0.241	1.05E-133	Proximal tubule cells	HADHB
8.09E-138	0.36607298	0.573	0.416	1.33E-133	Proximal tubule cells	NQO2
1.39E-137	0.41301236	0.284	0.148	2.30E-133	Proximal tubule cells	FYN
5.23E-133	0.42227729	0.345	0.201	8.62E-129	Proximal tubule cells	ARID5B
6.06E-132	0.34864546	0.619	0.474	9.99E-128	Proximal tubule cells	TMEM256
2.27E-130	0.3834656	0.402	0.25	3.74E-126	Proximal tubule cells	CLIC4
3.55E-130	0.39427709	0.307	0.17	5.85E-126	Proximal tubule cells	NUDT8
2.82E-128	0.31951688	0.676	0.54	4.64E-124	Proximal tubule cells	ATP5F1A
5.14E-128	0.42055487	0.457	0.309	8.46E-124	Proximal tubule cells	SNHG8
3.21E-127	0.36670449	0.479	0.326	5.28E-123	Proximal tubule cells	C1QBP
1.41E-126	0.37956506	0.467	0.315	2.31E-122	Proximal tubule cells	HAGH
8.62E-126	0.51010724	0.453	0.309	1.42E-121	Proximal tubule cells	AZGP1
8.74E-125	0.30688816	0.668	0.537	1.44E-120	Proximal tubule cells	PRDX3
1.75E-124	0.37035897	0.517	0.367	2.88E-120	Proximal tubule cells	AKR1C3
5.35E-124	0.35177649	0.426	0.273	8.81E-120	Proximal tubule cells	SMIM32
3.82E-123	0.37530585	0.341	0.2	6.30E-119	Proximal tubule cells	AGMAT
1.06E-122	0.40377866	0.526	0.386	1.74E-118	Proximal tubule cells	SIGIRR
7.15E-121	0.36117528	0.434	0.287	1.18E-116	Proximal tubule cells	ETFA
4.17E-120	0.36959552	0.418	0.271	6.86E-116	Proximal tubule cells	GAMT
1.44E-119	0.3502848	0.483	0.333	2.36E-115	Proximal tubule cells	SMS
2.58E-118	0.36921098	0.288	0.159	4.24E-114	Proximal tubule cells	GALM
1.06E-117	0.3514933	0.426	0.277	1.74E-113	Proximal tubule cells	BHMT2
1.78E-117	0.32745122	0.256	0.133	2.93E-113	Proximal tubule cells	LINC00671
2.72E-117	0.37417512	0.256	0.135	4.47E-113	Proximal tubule cells	CRYM
6.28E-117	0.33853392	0.411	0.265	1.03E-112	Proximal tubule cells	HIBADH
2.13E-116	0.37420458	0.319	0.187	3.50E-112	Proximal tubule cells	EPHX2
8.08E-116	0.3869675	0.284	0.159	1.33E-111	Proximal tubule cells	ENOSF1
7.57E-114	0.33963162	0.506	0.362	1.25E-109	Proximal tubule cells	CRYZ
1.66E-113	0.37097301	0.362	0.226	2.73E-109	Proximal tubule cells	SCRN2
2.73E-110	0.28010284	0.714	0.609	4.49E-106	Proximal tubule cells	PRR13
8.20E-110	0.32938695	0.464	0.32	1.35E-105	Proximal tubule cells	AK2
1.45E-108	0.29277541	0.666	0.545	2.38E-104	Proximal tubule cells	ATP5F1B
7.91E-103	0.2872923	0.655	0.543	1.30E-98	Proximal tubule cells	BRK1
2.85E-101	0.36547869	0.362	0.234	4.69E-97	Proximal tubule cells	AIFM1
5.72E-99	0.33523584	0.337	0.21	9.42E-95	Proximal tubule cells	CLDN10
1.42E-98	0.31888338	0.511	0.377	2.34E-94	Proximal tubule cells	DHRS4L2
6.46E-98	0.30497761	0.493	0.358	1.06E-93	Proximal tubule cells	NDUFV1
4.96E-96	0.26501992	0.658	0.539	8.17E-92	Proximal tubule cells	SCP2
3.51E-95	0.33049539	0.331	0.208	5.79E-91	Proximal tubule cells	IDH3G
2.80E-94	0.29027958	0.379	0.248	4.62E-90	Proximal tubule cells	TPM1
8.95E-94	0.25559119	0.663	0.543	1.47E-89	Proximal tubule cells	ATP5F1C
1.48E-93	0.30860605	0.478	0.346	2.43E-89	Proximal tubule cells	MDH2
3.19E-93	0.29059892	0.535	0.406	5.26E-89	Proximal tubule cells	VDAC1
3.42E-93	0.26179507	0.605	0.478	5.63E-89	Proximal tubule cells	UQCRFS1
3.99E-93	0.33581882	0.321	0.201	6.58E-89	Proximal tubule cells	ATP6V1B2
4.20E-93	0.3220156	0.415	0.286	6.92E-89	Proximal tubule cells	MORN2

2.76E-92	0.2812104	0.581	0.449	4.55E-88	Proximal tubule cells	PLA2G16
7.65E-92	0.31674275	0.341	0.218	1.26E-87	Proximal tubule cells	PXMP2
3.38E-90	0.31722591	0.364	0.241	5.56E-86	Proximal tubule cells	NDUFA10
4.12E-90	0.32538672	0.389	0.264	6.79E-86	Proximal tubule cells	ACADVL
3.96E-89	0.30657271	0.307	0.19	6.53E-85	Proximal tubule cells	MRPS35
1.04E-88	0.33399726	0.365	0.244	1.72E-84	Proximal tubule cells	TUBB4B
3.20E-88	0.31167068	0.357	0.235	5.27E-84	Proximal tubule cells	SLC6A13
8.59E-88	0.31953995	0.397	0.272	1.41E-83	Proximal tubule cells	FTCD
9.55E-88	0.32184225	0.392	0.269	1.57E-83	Proximal tubule cells	HEBP2
6.78E-87	0.33357305	0.261	0.153	1.12E-82	Proximal tubule cells	DHRS4
2.10E-86	0.27181274	0.504	0.37	3.45E-82	Proximal tubule cells	KRT8
1.62E-85	0.25825881	0.614	0.493	2.67E-81	Proximal tubule cells	NDUFS7
6.43E-83	0.27835001	0.523	0.399	1.06E-78	Proximal tubule cells	ESD
1.23E-82	0.32142009	0.374	0.255	2.03E-78	Proximal tubule cells	ZC3H15
4.26E-82	0.30156549	0.366	0.247	7.02E-78	Proximal tubule cells	TPMT
6.45E-82	0.31829857	0.292	0.183	1.06E-77	Proximal tubule cells	RDX
7.98E-81	0.30397821	0.27	0.164	1.31E-76	Proximal tubule cells	GRPEL1
1.69E-79	0.30692883	0.315	0.203	2.78E-75	Proximal tubule cells	GIPC2
2.52E-79	0.30232664	0.295	0.185	4.15E-75	Proximal tubule cells	ANGPTL3
3.14E-79	0.29381785	0.421	0.304	5.17E-75	Proximal tubule cells	UQCRC1
2.66E-78	0.29560855	0.391	0.273	4.38E-74	Proximal tubule cells	NDUFS2
6.19E-78	0.2722901	0.469	0.347	1.02E-73	Proximal tubule cells	CYC1
8.86E-78	0.27112959	0.516	0.394	1.46E-73	Proximal tubule cells	MSRA
2.66E-77	0.26993414	0.415	0.295	4.38E-73	Proximal tubule cells	ANAPC13
9.47E-77	0.30650498	0.26	0.159	1.56E-72	Proximal tubule cells	SDHA
1.15E-76	0.31317132	0.529	0.421	1.90E-72	Proximal tubule cells	LDHA
1.88E-76	0.27980213	0.505	0.386	3.09E-72	Proximal tubule cells	GSTO1
3.93E-76	0.3074833	0.307	0.2	6.47E-72	Proximal tubule cells	BIN1
1.95E-75	0.27886662	0.452	0.334	3.21E-71	Proximal tubule cells	MSRB2
4.18E-75	0.26587271	0.458	0.338	6.88E-71	Proximal tubule cells	IMPA2
1.25E-74	0.2757327	0.325	0.213	2.06E-70	Proximal tubule cells	ABHD14B
4.36E-74	0.27546475	0.437	0.319	7.18E-70	Proximal tubule cells	SLC9A3R1
6.46E-74	0.2998321	0.259	0.16	1.06E-69	Proximal tubule cells	PCK2
3.00E-72	0.27383388	0.444	0.325	4.94E-68	Proximal tubule cells	MPST
3.82E-72	0.2684402	0.502	0.381	6.29E-68	Proximal tubule cells	QPRT
6.76E-72	0.27285851	0.33	0.222	1.11E-67	Proximal tubule cells	CMPK1
1.88E-71	0.27136009	0.502	0.387	3.10E-67	Proximal tubule cells	PPA1
6.33E-71	0.26978324	0.46	0.343	1.04E-66	Proximal tubule cells	TUFM
7.05E-71	0.28638578	0.365	0.255	1.16E-66	Proximal tubule cells	UQCRC2
9.78E-70	0.27426991	0.276	0.176	1.61E-65	Proximal tubule cells	HMGCL
2.18E-69	0.26401056	0.391	0.278	3.59E-65	Proximal tubule cells	SUCLG2
2.21E-68	0.27211805	0.361	0.253	3.64E-64	Proximal tubule cells	SCOC
1.00E-67	0.26933942	0.334	0.228	1.65E-63	Proximal tubule cells	ALDH7A1
1.30E-67	0.25893491	0.471	0.359	2.15E-63	Proximal tubule cells	DECR1
5.58E-67	0.27303126	0.28	0.182	9.18E-63	Proximal tubule cells	CHP1
1.36E-65	0.26356584	0.33	0.225	2.24E-61	Proximal tubule cells	CCNG1
1.69E-65	0.27266386	0.261	0.166	2.79E-61	Proximal tubule cells	RENBP
2.58E-64	0.26821964	0.309	0.209	4.25E-60	Proximal tubule cells	RBPMS
1.60E-61	0.25215589	0.541	0.436	2.63E-57	Proximal tubule cells	GSTK1
1.87E-61	0.25577678	0.34	0.237	3.08E-57	Proximal tubule cells	STOML2
3.17E-61	0.26273663	0.279	0.184	5.23E-57	Proximal tubule cells	SLC39A4
2.80E-60	0.25602702	0.517	0.418	4.61E-56	Proximal tubule cells	PGK1
3.15E-59	0.27257255	0.257	0.168	5.20E-55	Proximal tubule cells	BORCS7
4.43E-58	0.25062778	0.356	0.256	7.30E-54	Proximal tubule cells	ADH5
2.70E-57	0.25292252	0.263	0.173	4.45E-53	Proximal tubule cells	ECHDC3
7.76E-45	0.27772813	0.324	0.239	1.28E-40	Proximal tubule cells	FOLR1
0	3.37643719	0.997	0.078	0	Proximal straight tubule cells	NEAT1
0	3.06062468	0.999	0.262	0	Proximal straight tubule cells	MALAT1

0	1.36884986	0.561	0.031	0	Proximal straight tubule cells	POLR2J3.1
0	1.35063258	0.665	0.08	0	Proximal straight tubule cells	VMP1
0	1.29058568	0.445	0.006	0	Proximal straight tubule cells	KCNQ1OT1
0	1.2845718	0.36	0.004	0	Proximal straight tubule cells	ABHD14A-ACY1
0	1.22583586	0.663	0.108	0	Proximal straight tubule cells	N4BP2L2
0	1.22561503	0.593	0.066	0	Proximal straight tubule cells	SYNE2
0	1.18150883	0.567	0.124	0	Proximal straight tubule cells	ELF3
0	1.15920442	0.558	0.07	0	Proximal straight tubule cells	PNISR
0	1.15135488	0.476	0.089	0	Proximal straight tubule cells	RNF213
0	1.11256478	0.453	0.033	0	Proximal straight tubule cells	MUC20-OT1
0	1.09369446	0.491	0.071	0	Proximal straight tubule cells	KIF12
0	1.08541634	0.582	0.116	0	Proximal straight tubule cells	RASSF4
0	1.06740611	0.779	0.267	0	Proximal straight tubule cells	ACSM2A
0	1.03307328	0.669	0.173	0	Proximal straight tubule cells	ZBTB20
0	0.98854135	0.41	0.033	0	Proximal straight tubule cells	UNC5CL
0	0.98665983	0.82	0.326	0	Proximal straight tubule cells	ACSM2B
0	0.97296142	0.446	0.039	0	Proximal straight tubule cells	WSB1
0	0.95159446	0.53	0.123	0	Proximal straight tubule cells	LINC01320
0	0.93801607	0.551	0.122	0	Proximal straight tubule cells	PAX8
0	0.91453489	0.379	0.043	0	Proximal straight tubule cells	HOOK2
0	0.90879506	0.521	0.1	0	Proximal straight tubule cells	DDX17
0	0.88896263	0.433	0.083	0	Proximal straight tubule cells	SLC47A2
0	0.85499281	0.327	0.019	0	Proximal straight tubule cells	SORBS2
0	0.7945897	0.316	0.018	0	Proximal straight tubule cells	PKHD1
0	0.76986733	0.383	0.071	0	Proximal straight tubule cells	IL17RB
0	0.74765228	0.3	0.013	0	Proximal straight tubule cells	FTX
0	0.73203789	0.328	0.05	0	Proximal straight tubule cells	PPFIBP1
0	0.71525284	0.348	0.044	0	Proximal straight tubule cells	PABPN1
0	0.71151398	0.371	0.066	0	Proximal straight tubule cells	CHD9
0	0.707686	0.364	0.057	0	Proximal straight tubule cells	NRP1
0	0.69430189	0.283	0.028	0	Proximal straight tubule cells	TRPM3
0	0.68884937	0.272	0.021	0	Proximal straight tubule cells	RBM6
0	0.68628116	0.282	0.02	0	Proximal straight tubule cells	VPS13A
0	0.65161168	0.285	0.035	0	Proximal straight tubule cells	PATJ
0	0.63521798	0.263	0.026	0	Proximal straight tubule cells	PAX2
0	0.63485528	0.276	0.036	0	Proximal straight tubule cells	SLC25A37
0	0.63094674	0.288	0.036	0	Proximal straight tubule cells	CCNL1
0	0.62603541	0.278	0.032	0	Proximal straight tubule cells	ZKSCAN1
0	0.60547689	0.258	0.024	0	Proximal straight tubule cells	ARHGEF28
0	0.58740335	0.259	0.026	0	Proximal straight tubule cells	NKTR
0	0.57960842	0.256	0.029	0	Proximal straight tubule cells	RSRP1
7.32E-307	0.64683225	0.281	0.04	1.21E-302	Proximal straight tubule cells	GPR155
5.64E-296	0.86473263	0.407	0.085	9.29E-292	Proximal straight tubule cells	HNRNPH1
1.24E-294	0.62155416	0.297	0.046	2.04E-290	Proximal straight tubule cells	SLC17A1
2.44E-287	0.64826667	0.353	0.065	4.01E-283	Proximal straight tubule cells	LUC7L3
6.52E-271	0.70712731	0.465	0.115	1.07E-266	Proximal straight tubule cells	SRRM2
1.16E-269	0.71049372	0.371	0.076	1.90E-265	Proximal straight tubule cells	ARHGAP29
7.31E-267	0.62897966	0.32	0.057	1.20E-262	Proximal straight tubule cells	AKAP9
8.27E-267	0.62977687	0.289	0.048	1.36E-262	Proximal straight tubule cells	SLC47A1
1.03E-264	0.58104274	0.271	0.042	1.70E-260	Proximal straight tubule cells	OGA
8.06E-258	0.81242956	0.758	0.3	1.33E-253	Proximal straight tubule cells	DDX5
2.54E-256	0.78558495	0.636	0.212	4.19E-252	Proximal straight tubule cells	LRP2
1.59E-253	1.19359932	0.322	0.066	2.62E-249	Proximal straight tubule cells	SLC22A7
1.66E-246	0.48810989	0.251	0.038	2.74E-242	Proximal straight tubule cells	ASH1L
1.06E-239	0.74871965	0.392	0.093	1.74E-235	Proximal straight tubule cells	HSPA1A
6.30E-236	0.69568898	0.477	0.133	1.04E-231	Proximal straight tubule cells	FARP1
3.84E-235	0.88490479	0.636	0.233	6.32E-231	Proximal straight tubule cells	CUBN
5.08E-234	0.52663119	0.255	0.042	8.36E-230	Proximal straight tubule cells	FUT6

9.58E-228	0.55932183	0.286	0.053	1.58E-223	Proximal straight tubule cells	THUMPD3-AS1
2.83E-216	0.7752809	0.722	0.302	4.66E-212	Proximal straight tubule cells	BHMT2
8.43E-209	0.49237147	0.26	0.048	1.39E-204	Proximal straight tubule cells	RBM25
1.05E-208	0.60497772	0.387	0.099	1.73E-204	Proximal straight tubule cells	MYO6
8.81E-208	0.88615321	0.483	0.154	1.45E-203	Proximal straight tubule cells	LINC00671
5.77E-206	0.58496958	0.272	0.053	9.50E-202	Proximal straight tubule cells	CBX6
1.86E-200	0.58310363	0.375	0.096	3.06E-196	Proximal straight tubule cells	AAK1
3.74E-198	0.81741	0.373	0.098	6.17E-194	Proximal straight tubule cells	INSR
7.08E-197	0.52911788	0.297	0.064	1.17E-192	Proximal straight tubule cells	MIA2
2.51E-188	0.67228454	0.539	0.189	4.14E-184	Proximal straight tubule cells	SLC13A3
5.45E-186	0.48595201	0.257	0.051	8.98E-182	Proximal straight tubule cells	NUMB
9.03E-178	0.6193069	0.266	0.058	1.49E-173	Proximal straight tubule cells	MST1
7.23E-176	0.51687908	0.301	0.072	1.19E-171	Proximal straight tubule cells	SF1
1.01E-167	0.54297549	0.502	0.173	1.66E-163	Proximal straight tubule cells	RBM39
7.13E-160	0.46107103	0.263	0.06	1.17E-155	Proximal straight tubule cells	STAT3
1.07E-158	0.5518341	0.327	0.09	1.76E-154	Proximal straight tubule cells	SNRNP70
3.22E-158	0.51251446	0.292	0.073	5.30E-154	Proximal straight tubule cells	FNIP2
5.30E-158	0.50994191	0.303	0.078	8.72E-154	Proximal straight tubule cells	TRIM38
5.91E-157	0.5225433	0.299	0.076	9.73E-153	Proximal straight tubule cells	ITGB8
1.41E-156	0.52755159	0.336	0.094	2.32E-152	Proximal straight tubule cells	GDA
4.66E-156	0.4980288	0.371	0.11	7.67E-152	Proximal straight tubule cells	ACTN4
5.42E-153	0.66648839	0.714	0.361	8.92E-149	Proximal straight tubule cells	TNFSF10
1.10E-152	0.51441852	0.379	0.115	1.81E-148	Proximal straight tubule cells	KTN1
1.34E-152	0.46331086	0.281	0.069	2.21E-148	Proximal straight tubule cells	ZNF207
2.78E-150	0.44426631	0.254	0.059	4.57E-146	Proximal straight tubule cells	CTNND1
4.95E-150	0.46373035	0.287	0.073	8.15E-146	Proximal straight tubule cells	GOLGA4
2.62E-149	0.46891152	0.293	0.076	4.32E-145	Proximal straight tubule cells	ARGLU1
1.54E-148	0.48834233	0.292	0.076	2.54E-144	Proximal straight tubule cells	TNRC6B
7.79E-147	0.49474628	0.375	0.116	1.28E-142	Proximal straight tubule cells	GTF2I
2.45E-146	0.56085791	0.417	0.139	4.04E-142	Proximal straight tubule cells	BCAM
9.22E-146	0.49063187	0.352	0.105	1.52E-141	Proximal straight tubule cells	TAF1D
6.46E-145	0.47478956	0.291	0.077	1.06E-140	Proximal straight tubule cells	MPHOSPH8
8.11E-143	0.51333184	0.382	0.122	1.34E-138	Proximal straight tubule cells	SRSF11
7.16E-134	0.540465	0.987	0.838	1.18E-129	Proximal straight tubule cells	SPP1
5.31E-132	0.4865359	0.314	0.092	8.74E-128	Proximal straight tubule cells	KCNJ16
1.98E-128	0.46085901	0.292	0.084	3.26E-124	Proximal straight tubule cells	RAB3IP
1.42E-127	0.45399391	0.317	0.096	2.34E-123	Proximal straight tubule cells	FUS
2.78E-127	0.63250503	0.414	0.153	4.58E-123	Proximal straight tubule cells	SLC5A12
7.70E-126	0.42121596	0.256	0.068	1.27E-121	Proximal straight tubule cells	CPM
6.05E-123	0.54586829	0.421	0.158	9.96E-119	Proximal straight tubule cells	ZFP36L1
2.69E-122	0.50162557	0.401	0.144	4.42E-118	Proximal straight tubule cells	RETREG1
3.31E-122	0.50628533	0.288	0.085	5.46E-118	Proximal straight tubule cells	JUN
4.10E-119	0.42495309	0.276	0.08	6.76E-115	Proximal straight tubule cells	UGCG
4.44E-117	0.41435581	0.288	0.086	7.32E-113	Proximal straight tubule cells	LPP
1.63E-109	0.58663023	0.332	0.116	2.68E-105	Proximal straight tubule cells	KLF6
4.16E-109	0.38008961	0.289	0.09	6.86E-105	Proximal straight tubule cells	SF3B1
6.76E-108	0.4605991	0.395	0.15	1.11E-103	Proximal straight tubule cells	CDH6
3.84E-107	0.4699626	0.553	0.252	6.33E-103	Proximal straight tubule cells	AQP1
2.67E-106	0.4048312	0.268	0.081	4.39E-102	Proximal straight tubule cells	TINAGL1
2.61E-105	0.39735213	0.274	0.084	4.29E-101	Proximal straight tubule cells	NTN4
2.88E-103	0.61561809	0.271	0.089	4.74E-99	Proximal straight tubule cells	CYP4A11
5.09E-103	0.47808807	0.299	0.1	8.39E-99	Proximal straight tubule cells	ADAMTS9-AS1
6.45E-102	0.39819455	0.271	0.086	1.06E-97	Proximal straight tubule cells	QTRT1
1.05E-101	0.48416261	0.432	0.179	1.73E-97	Proximal straight tubule cells	HIF1A
2.15E-101	0.46877991	0.372	0.142	3.54E-97	Proximal straight tubule cells	MAT2A
8.62E-101	0.44244056	0.253	0.078	1.42E-96	Proximal straight tubule cells	SRSF6
1.94E-100	0.39690342	0.271	0.086	3.19E-96	Proximal straight tubule cells	CHDH
6.43E-98	0.4516875	0.487	0.212	1.06E-93	Proximal straight tubule cells	PIGR

1.77E-92	0.50819154	0.586	0.3	2.92E-88	Proximal straight tubule cells	CEBPD
4.65E-92	0.33627422	0.274	0.09	7.66E-88	Proximal straight tubule cells	ACLY
2.72E-91	0.39502488	0.378	0.15	4.48E-87	Proximal straight tubule cells	SRSF2
5.28E-91	0.38270361	0.331	0.123	8.70E-87	Proximal straight tubule cells	PLEKHA1
1.00E-90	0.42312521	0.417	0.175	1.65E-86	Proximal straight tubule cells	SLC22A2
1.20E-90	0.39299823	0.313	0.113	1.98E-86	Proximal straight tubule cells	ANK2
3.00E-90	0.39007138	0.321	0.119	4.94E-86	Proximal straight tubule cells	CFLAR
8.44E-90	0.44180212	0.461	0.205	1.39E-85	Proximal straight tubule cells	KCNJ15
2.59E-89	0.36877896	0.317	0.116	4.26E-85	Proximal straight tubule cells	PLEKHA5
1.81E-83	0.41132053	0.324	0.126	2.98E-79	Proximal straight tubule cells	FHIT
8.60E-83	0.50589189	0.359	0.149	1.42E-78	Proximal straight tubule cells	VCAM1
1.49E-82	0.36641113	0.325	0.125	2.46E-78	Proximal straight tubule cells	SLC5A2
6.18E-82	0.41394284	0.429	0.191	1.02E-77	Proximal straight tubule cells	ARHGAP24
8.15E-82	0.36471184	0.282	0.102	1.34E-77	Proximal straight tubule cells	SCAF11
1.15E-78	0.33404457	0.294	0.111	1.89E-74	Proximal straight tubule cells	EPS8L2
9.57E-78	0.36842937	0.274	0.101	1.58E-73	Proximal straight tubule cells	PARD6B
1.20E-76	0.35459601	0.353	0.146	1.97E-72	Proximal straight tubule cells	SDC4
8.82E-76	0.33003802	0.29	0.11	1.45E-71	Proximal straight tubule cells	PUM3
2.07E-75	0.36444377	0.415	0.188	3.42E-71	Proximal straight tubule cells	LEPROT
2.36E-75	0.37327669	0.397	0.176	3.89E-71	Proximal straight tubule cells	NFIB
4.54E-75	0.40204213	0.301	0.118	7.49E-71	Proximal straight tubule cells	SOX4
5.40E-75	0.27750211	0.263	0.094	8.90E-71	Proximal straight tubule cells	TGOLN2
2.35E-74	0.36924405	0.544	0.277	3.87E-70	Proximal straight tubule cells	CALD1
2.80E-74	0.43079796	0.331	0.138	4.61E-70	Proximal straight tubule cells	CLDN2
7.03E-73	0.37129811	0.395	0.178	1.16E-68	Proximal straight tubule cells	HPN
1.52E-72	0.35981833	0.538	0.272	2.50E-68	Proximal straight tubule cells	AMN
2.81E-72	0.34829566	0.36	0.155	4.63E-68	Proximal straight tubule cells	APP
5.45E-72	0.32490002	0.26	0.096	8.97E-68	Proximal straight tubule cells	ARL16
7.55E-72	0.36949849	0.361	0.156	1.24E-67	Proximal straight tubule cells	CTTN
5.62E-71	0.48544353	0.562	0.303	9.26E-67	Proximal straight tubule cells	SLC13A1
1.70E-70	0.40512067	0.343	0.149	2.79E-66	Proximal straight tubule cells	SLC39A5
2.11E-70	0.3766073	0.385	0.174	3.48E-66	Proximal straight tubule cells	EMX2
1.17E-69	0.39699565	0.514	0.264	1.92E-65	Proximal straight tubule cells	ATP1A1
1.49E-68	0.38884447	0.482	0.244	2.46E-64	Proximal straight tubule cells	SRSF5
1.51E-67	0.4336795	0.297	0.123	2.49E-63	Proximal straight tubule cells	SLC34A1
2.10E-66	0.31742286	0.416	0.194	3.45E-62	Proximal straight tubule cells	CFI
3.73E-61	0.34058176	0.501	0.264	6.14E-57	Proximal straight tubule cells	RHOB
1.40E-60	0.29691295	0.462	0.232	2.31E-56	Proximal straight tubule cells	SON
7.12E-60	0.29705807	0.389	0.185	1.17E-55	Proximal straight tubule cells	CCDC198
1.82E-59	0.38531606	0.405	0.2	2.99E-55	Proximal straight tubule cells	SLC16A9
2.45E-59	0.27834187	0.342	0.153	4.03E-55	Proximal straight tubule cells	CNPY2
7.92E-59	0.27022695	0.355	0.161	1.30E-54	Proximal straight tubule cells	ENPEP
5.43E-57	0.30237081	0.381	0.185	8.95E-53	Proximal straight tubule cells	RAB29
1.17E-56	0.31799102	0.597	0.347	1.93E-52	Proximal straight tubule cells	TSPAN1
5.68E-56	0.31982592	0.29	0.128	9.36E-52	Proximal straight tubule cells	SRSF7
6.32E-56	0.40196657	0.422	0.218	1.04E-51	Proximal straight tubule cells	EPHX2
6.57E-55	0.28545374	0.319	0.145	1.08E-50	Proximal straight tubule cells	RBM47
3.03E-54	0.25220066	0.253	0.104	4.99E-50	Proximal straight tubule cells	TNFRSF1A
1.44E-53	0.30923168	0.932	0.726	2.37E-49	Proximal straight tubule cells	NAT8
1.76E-53	0.2754569	0.32	0.147	2.90E-49	Proximal straight tubule cells	AHCYL1
2.49E-53	0.27617377	0.342	0.161	4.10E-49	Proximal straight tubule cells	HNRNPU
4.84E-51	0.27197702	0.437	0.229	7.97E-47	Proximal straight tubule cells	RBPMS
9.02E-51	0.26059091	0.476	0.254	1.49E-46	Proximal straight tubule cells	PTH1R
1.83E-50	0.29500099	0.334	0.161	3.02E-46	Proximal straight tubule cells	NME3
2.46E-50	0.32837811	0.74	0.476	4.05E-46	Proximal straight tubule cells	ATP1B1
8.36E-50	0.36262623	0.524	0.31	1.38E-45	Proximal straight tubule cells	HIST1H4C
1.98E-46	0.28689443	0.343	0.172	3.26E-42	Proximal straight tubule cells	SLC4A4
2.30E-46	0.27772283	0.303	0.143	3.79E-42	Proximal straight tubule cells	BICC1

4.07E-46	0.27305549	0.436	0.236	6.70E-42	Proximal straight tubule cells	YBX3
3.74E-45	0.27488823	0.562	0.334	6.16E-41	Proximal straight tubule cells	ANPEP
1.44E-42	0.28616006	0.308	0.154	2.36E-38	Proximal straight tubule cells	SLC22A12
5.25E-42	0.26822022	0.423	0.233	8.65E-38	Proximal straight tubule cells	LRPAP1
2.98E-39	0.25625493	0.276	0.136	4.91E-35	Proximal straight tubule cells	RCAN1
1.88E-36	0.2920304	0.692	0.465	3.09E-32	Proximal straight tubule cells	BHMT
4.36E-36	0.25863143	0.251	0.123	7.17E-32	Proximal straight tubule cells	SGK1
2.04E-34	0.25084078	0.787	0.556	3.36E-30	Proximal straight tubule cells	HMGN3
8.22E-29	0.72169725	0.327	0.214	1.35E-24	Proximal straight tubule cells	RBP4
0	3.5157495	0.468	0.005	0	NK-T cells	GNLY
0	3.36972348	0.613	0.007	0	NK-T cells	NKG7
0	3.30640076	0.67	0.004	0	NK-T cells	CCL5
0	2.86050164	0.892	0.03	0	NK-T cells	CD52
0	2.67747292	1	0.368	0	NK-T cells	TMSB4X
0	2.50083872	0.59	0.002	0	NK-T cells	GZMA
0	2.45843576	0.966	0.208	0	NK-T cells	HLA-B
0	2.43664269	0.446	0.002	0	NK-T cells	GZMB
0	2.42841461	0.747	0.002	0	NK-T cells	CD3D
0	2.39992872	0.944	0.248	0	NK-T cells	HLA-C
0	2.3911967	0.493	0.002	0	NK-T cells	GZMH
0	2.3415803	0.667	0.004	0	NK-T cells	TRAC
0	2.33723777	1	0.816	0	NK-T cells	B2M
0	2.23623213	0.931	0.285	0	NK-T cells	HLA-A
0	2.23613902	0.76	0.024	0	NK-T cells	HCST
0	2.2088401	0.553	0.002	0	NK-T cells	CST7
0	2.19027678	0.855	0.048	0	NK-T cells	S100A4
0	2.18378415	0.802	0.034	0	NK-T cells	ARHGDIB
0	2.13511263	0.758	0.024	0	NK-T cells	CORO1A
0	2.1095504	0.431	0.002	0	NK-T cells	CCL4
0	2.08564176	0.465	0.021	0	NK-T cells	LTB
0	2.04639884	0.547	0.001	0	NK-T cells	TRBC1
0	1.99943052	0.574	0.003	0	NK-T cells	TRBC2
0	1.98199449	0.999	0.274	0	NK-T cells	MALAT1
0	1.97401957	0.711	0.021	0	NK-T cells	PTPRC
0	1.95864408	0.838	0.113	0	NK-T cells	HLA-E
0	1.92496891	0.599	0.075	0	NK-T cells	JUN
0	1.91963729	0.636	0.001	0	NK-T cells	CD3E
0	1.9160712	0.39	0.001	0	NK-T cells	FGFBP2
0	1.91076198	0.85	0.187	0	NK-T cells	BTG1
0	1.9047613	0.442	0.001	0	NK-T cells	PRF1
0	1.86438272	0.321	0.001	0	NK-T cells	KLRB1
0	1.80754519	0.552	0.001	0	NK-T cells	CD2
0	1.78596002	0.679	0.035	0	NK-T cells	EMP3
0	1.76610309	0.257	0.001	0	NK-T cells	TRDC
0	1.71848685	0.547	0.018	0	NK-T cells	ANXA1
0	1.71234317	0.586	0.117	0	NK-T cells	JUNB
0	1.67223182	0.466	0.013	0	NK-T cells	CXCR4
0	1.63761698	0.558	0.013	0	NK-T cells	RAC2
0	1.63571252	0.585	0.025	0	NK-T cells	FXYD5
0	1.60281438	0.424	0.004	0	NK-T cells	CD69
0	1.60046388	0.337	0.001	0	NK-T cells	KLRD1
0	1.59758658	0.658	0.081	0	NK-T cells	IFITM2
0	1.58723	0.618	0.028	0	NK-T cells	GMFG
0	1.56183995	0.336	0	0	NK-T cells	CTSW
0	1.55585624	0.425	0.001	0	NK-T cells	CD7
0	1.54223705	0.633	0.033	0	NK-T cells	SRGN
0	1.53244787	0.492	0.007	0	NK-T cells	GIMAP7
0	1.50860197	0.512	0.011	0	NK-T cells	GIMAP4

0	1.49282201	0.469	0.004	0	NK-T cells	IL2RG
0	1.48399213	0.529	0.026	0	NK-T cells	EVL
0	1.4710169	0.985	0.816	0	NK-T cells	ACTB
0	1.4633549	0.49	0.024	0	NK-T cells	ITGB2
0	1.45236793	0.283	0	0	NK-T cells	TRGC2
0	1.43102595	0.423	0.001	0	NK-T cells	CD247
0	1.43027526	0.709	0.198	0	NK-T cells	TXNIP
0	1.40439393	0.479	0.02	0	NK-T cells	LIMD2
0	1.4021802	0.475	0.016	0	NK-T cells	CD53
0	1.40204927	0.573	0.082	0	NK-T cells	CD99
0	1.39254367	0.341	0.001	0	NK-T cells	HOPX
0	1.38805381	0.417	0.001	0	NK-T cells	CD3G
0	1.37921922	0.509	0.027	0	NK-T cells	LAPTM5
0	1.37689872	0.435	0.001	0	NK-T cells	LCK
0	1.37673286	0.391	0.001	0	NK-T cells	GZMM
0	1.37223378	0.411	0.003	0	NK-T cells	IFITM1
0	1.36441866	0.486	0.033	0	NK-T cells	LSP1
0	1.36402383	0.998	0.846	0	NK-T cells	RPS3
0	1.3543109	0.41	0.012	0	NK-T cells	KLF2
0	1.3482837	0.488	0.023	0	NK-T cells	CD37
0	1.33053164	0.877	0.432	0	NK-T cells	SH3BGRL3
0	1.29253107	0.285	0.001	0	NK-T cells	IL7R
0	1.29202859	0.459	0.019	0	NK-T cells	GPSM3
0	1.27475878	0.999	0.862	0	NK-T cells	RPS2
0	1.27044283	0.427	0.02	0	NK-T cells	CD48
0	1.2673967	0.5	0.067	0	NK-T cells	PSMB9
0	1.25799506	0.454	0.068	0	NK-T cells	DUSP1
0	1.24610152	0.382	0.007	0	NK-T cells	BIN2
0	1.23471615	0.447	0.072	0	NK-T cells	IER2
0	1.23339206	0.996	0.919	0	NK-T cells	RPS19
0	1.20940989	0.325	0.002	0	NK-T cells	ITM2A
0	1.19677287	0.394	0.061	0	NK-T cells	LGALS1
0	1.18953692	0.364	0.016	0	NK-T cells	PLAC8
0	1.17896403	0.251	0.004	0	NK-T cells	DUSP2
0	1.17290421	0.366	0.007	0	NK-T cells	ACAP1
0	1.15328966	0.344	0.014	0	NK-T cells	ISG20
0	1.13596541	0.508	0.103	0	NK-T cells	ARL6IP5
0	1.11735495	0.366	0.019	0	NK-T cells	TRAF3IP3
0	1.11688309	0.302	0.004	0	NK-T cells	KLRG1
0	1.10343783	0.35	0.017	0	NK-T cells	LCP1
0	1.09108255	0.291	0.011	0	NK-T cells	LYAR
0	1.08892118	0.386	0.047	0	NK-T cells	POLR2J3.1
0	1.08566178	1	0.969	0	NK-T cells	RPS27
0	1.08504404	0.343	0.004	0	NK-T cells	TBC1D10C
0	1.06919673	0.291	0.007	0	NK-T cells	ALOX5AP
0	1.0683458	0.316	0.002	0	NK-T cells	LAT
0	1.06741845	0.314	0.005	0	NK-T cells	CLEC2D
0	1.06400188	0.319	0.007	0	NK-T cells	IKZF1
0	1.04388246	0.32	0.017	0	NK-T cells	UCP2
0	1.01865836	0.319	0.012	0	NK-T cells	HLA-F
0	1.01109203	0.344	0.026	0	NK-T cells	STK4
0	0.99514503	0.315	0.016	0	NK-T cells	GYPC
0	0.99085852	0.286	0.003	0	NK-T cells	ARL4C
0	0.99046036	0.316	0.038	0	NK-T cells	IRF1
0	0.98634653	0.293	0.007	0	NK-T cells	CYTIP
0	0.98441899	0.261	0.005	0	NK-T cells	APOBEC3G
0	0.97771877	0.251	0.001	0	NK-T cells	LINC00861
0	0.96522839	0.319	0.017	0	NK-T cells	CD44

0	0.95494925	0.285	0.014	0	NK-T cells	MYO1F
0	0.94201403	0.279	0.012	0	NK-T cells	CRIP1
0	0.93885989	0.307	0.029	0	NK-T cells	CD47
0	0.93239423	0.342	0.045	0	NK-T cells	FOS
0	0.91800014	0.296	0.029	0	NK-T cells	CCND3
0	0.90475186	0.264	0.01	0	NK-T cells	PPP1R18
0	0.89114897	0.253	0.007	0	NK-T cells	CLEC2B
0	0.88687607	0.995	0.929	0	NK-T cells	RPL27A
0	0.88133735	0.268	0.016	0	NK-T cells	IFI16
0	0.86008651	0.257	0.02	0	NK-T cells	STK17A
0	0.85986685	0.252	0.011	0	NK-T cells	STK17B
0	0.85341266	0.999	0.98	0	NK-T cells	RPS18
0	0.83997473	0.252	0.019	0	NK-T cells	PCSK7
0	0.83983805	0.999	0.955	0	NK-T cells	RPLP2
0	0.78250554	0.81	0.16	0	NK-T cells	S100A6
0	0.74286239	1	0.991	0	NK-T cells	RPL10
2.00E-307	0.75636151	0.999	0.977	3.29E-303	NK-T cells	RPS14
6.71E-307	1.24837893	0.498	0.107	1.11E-302	NK-T cells	ZFP36L2
2.02E-301	0.80738081	0.996	0.923	3.33E-297	NK-T cells	RPL28
6.95E-295	0.80430041	0.999	0.958	1.14E-290	NK-T cells	RPS15A
2.15E-291	0.95830573	0.343	0.051	3.54E-287	NK-T cells	FYB1
3.51E-291	1.27521093	0.395	0.07	5.79E-287	NK-T cells	TSC22D3
5.20E-289	0.68612659	1	0.989	8.57E-285	NK-T cells	RPL13A
2.74E-287	0.76510066	0.993	0.936	4.51E-283	NK-T cells	RPL23A
2.47E-283	1.06567137	0.467	0.099	4.07E-279	NK-T cells	AES
3.15E-281	0.90624113	0.997	0.705	5.19E-277	NK-T cells	TMSB10
2.74E-274	1.07289369	0.359	0.06	4.52E-270	NK-T cells	HSPA1B
6.85E-267	0.85066825	0.988	0.875	1.13E-262	NK-T cells	RPL30
3.88E-263	0.76639775	0.997	0.932	6.39E-259	NK-T cells	RPL18A
8.22E-254	0.67326793	1	0.989	1.35E-249	NK-T cells	RPL13
1.18E-251	0.75921806	0.259	0.033	1.95E-247	NK-T cells	RSRP1
3.90E-246	0.63846564	0.997	0.979	6.43E-242	NK-T cells	RPS27A
4.89E-246	1.04731391	0.309	0.049	8.05E-242	NK-T cells	H1FX
6.36E-240	1.08077407	0.749	0.307	1.05E-235	NK-T cells	DDX5
6.35E-236	0.90332499	0.995	0.891	1.05E-231	NK-T cells	RPS29
3.22E-230	0.9942042	0.381	0.079	5.31E-226	NK-T cells	PPP2R5C
1.40E-216	1.05973073	0.371	0.079	2.30E-212	NK-T cells	DNAJB1
9.38E-215	0.84341141	0.321	0.059	1.54E-210	NK-T cells	ANXA6
4.91E-212	1.02601212	0.863	0.532	8.09E-208	NK-T cells	MYL12A
3.67E-204	0.88057332	0.251	0.038	6.05E-200	NK-T cells	CYTOR
5.73E-204	0.659854	0.998	0.972	9.44E-200	NK-T cells	RPS12
6.09E-204	0.81051314	0.329	0.064	1.00E-199	NK-T cells	1-Sep
2.11E-202	0.76340662	0.967	0.816	3.48E-198	NK-T cells	RPL18
8.67E-202	0.94212132	0.284	0.05	1.43E-197	NK-T cells	EFHD2
1.49E-197	0.84664077	0.377	0.085	2.46E-193	NK-T cells	PNISR
4.97E-192	0.80965273	0.289	0.053	8.19E-188	NK-T cells	FAM49B
3.55E-190	0.56801067	0.993	0.971	5.85E-186	NK-T cells	RPL3
3.30E-188	0.70147169	0.998	0.955	5.44E-184	NK-T cells	RPL21
1.51E-186	0.7898513	0.978	0.854	2.49E-182	NK-T cells	RPL39
1.06E-180	1.06151352	0.781	0.41	1.74E-176	NK-T cells	ACTG1
1.44E-180	0.84694643	0.943	0.772	2.37E-176	NK-T cells	PFN1
1.45E-180	0.78521641	0.317	0.066	2.39E-176	NK-T cells	ICAM3
4.02E-178	0.75762452	0.38	0.093	6.62E-174	NK-T cells	HLA-DPB1
2.00E-177	0.85698963	0.442	0.126	3.30E-173	NK-T cells	N4BP2L2
1.63E-172	0.76757957	0.918	0.697	2.68E-168	NK-T cells	RPSA
1.66E-167	0.54837419	1	0.986	2.74E-163	NK-T cells	RPL32
1.91E-163	0.58860654	0.992	0.94	3.15E-159	NK-T cells	RPS4X
3.87E-162	0.97453107	0.694	0.344	6.37E-158	NK-T cells	ARPC2

4.27E-159	0.90501304	0.478	0.16	7.03E-155	NK-T cells	7-Sep
5.88E-146	0.48337171	0.99	0.94	9.68E-142	NK-T cells	RPL15
3.29E-145	0.90413766	0.405	0.126	5.42E-141	NK-T cells	SRSF7
2.43E-144	0.79006842	0.355	0.098	4.01E-140	NK-T cells	FUS
9.02E-144	0.86604237	0.418	0.133	1.49E-139	NK-T cells	LITAF
1.43E-136	1.05425188	0.347	0.1	2.35E-132	NK-T cells	HSPA1A
1.43E-136	0.74161713	0.481	0.164	2.36E-132	NK-T cells	VIM
1.58E-134	0.83830628	0.296	0.074	2.61E-130	NK-T cells	HMGB2
2.51E-132	0.41770942	0.997	0.982	4.14E-128	NK-T cells	RPS15
6.06E-131	0.47712295	0.998	0.984	9.98E-127	NK-T cells	RPS6
1.08E-130	0.49125396	0.979	0.902	1.78E-126	NK-T cells	FAU
3.43E-130	0.46951503	0.996	0.976	5.66E-126	NK-T cells	RPL11
1.03E-129	0.46472603	0.992	0.962	1.70E-125	NK-T cells	RPS3A
1.48E-129	0.79795459	0.331	0.093	2.43E-125	NK-T cells	XBP1
1.68E-129	0.86690079	0.319	0.088	2.77E-125	NK-T cells	ITGB1
1.71E-128	0.49148356	0.989	0.953	2.82E-124	NK-T cells	RPL35A
9.51E-124	0.63148631	0.254	0.059	1.57E-119	NK-T cells	PRRC2C
5.24E-121	0.4879445	0.987	0.895	8.64E-117	NK-T cells	PTMA
1.32E-119	0.51789373	0.974	0.884	2.18E-115	NK-T cells	RPL10A
2.43E-117	0.63190305	0.251	0.06	4.00E-113	NK-T cells	DHRS7
1.52E-115	0.45374943	0.995	0.962	2.50E-111	NK-T cells	RPS25
9.05E-113	0.68076712	0.845	0.602	1.49E-108	NK-T cells	H3F3B
4.09E-112	0.80748403	0.361	0.119	6.74E-108	NK-T cells	KLF6
1.15E-111	0.69268572	0.836	0.559	1.89E-107	NK-T cells	CYBA
8.72E-111	0.72009221	0.33	0.103	1.44E-106	NK-T cells	PAXX
1.28E-110	0.43072045	1	0.994	2.11E-106	NK-T cells	RPL34
3.38E-109	0.35828486	1	0.996	5.56E-105	NK-T cells	RPL41
2.68E-107	0.74617002	0.4	0.145	4.41E-103	NK-T cells	ARPC1B
6.81E-106	0.58463432	0.273	0.073	1.12E-101	NK-T cells	HLA-DPA1
3.92E-105	0.69276037	0.327	0.104	6.46E-101	NK-T cells	PSMB8
4.90E-101	0.55056173	0.901	0.705	8.08E-97	NK-T cells	RPS21
8.20E-101	0.70068406	0.284	0.085	1.35E-96	NK-T cells	OSTF1
4.04E-100	1.01050825	0.514	0.252	6.65E-96	NK-T cells	ID2
4.44E-100	0.63590836	0.879	0.704	7.32E-96	NK-T cells	CFL1
5.45E-100	0.65637478	0.827	0.591	8.97E-96	NK-T cells	RPS10
9.65E-99	0.72886422	0.541	0.263	1.59E-94	NK-T cells	SSR2
1.60E-98	0.47034814	0.984	0.935	2.63E-94	NK-T cells	RPL36
6.28E-98	0.3851101	0.991	0.977	1.03E-93	NK-T cells	RPL19
2.19E-95	0.50015276	0.917	0.762	3.61E-91	NK-T cells	EEF1D
4.10E-94	0.70117549	0.56	0.281	6.75E-90	NK-T cells	SARAF
1.47E-88	0.70073567	0.608	0.351	2.42E-84	NK-T cells	NOP53
1.71E-87	0.46195379	0.989	0.955	2.82E-83	NK-T cells	RPS8
3.52E-86	0.37523907	0.989	0.958	5.80E-82	NK-T cells	RPL31
2.39E-85	0.3601311	0.988	0.945	3.94E-81	NK-T cells	RPL35
3.93E-85	0.66817293	0.413	0.174	6.48E-81	NK-T cells	CAP1
7.11E-82	0.69552862	0.39	0.163	1.17E-77	NK-T cells	ACTR3
4.48E-81	0.64777763	0.462	0.218	7.38E-77	NK-T cells	VAMP2
1.98E-77	0.60381242	0.266	0.087	3.27E-73	NK-T cells	ZFP36
5.35E-77	0.59326021	0.288	0.099	8.81E-73	NK-T cells	LEPROTL1
3.36E-75	0.37170714	0.973	0.92	5.53E-71	NK-T cells	RPS9
1.69E-73	0.33985575	0.999	0.99	2.78E-69	NK-T cells	RPLP1
1.91E-72	0.33252929	0.993	0.98	3.15E-68	NK-T cells	RPS28
2.10E-70	0.5494831	0.27	0.095	3.46E-66	NK-T cells	TERF2IP
2.38E-68	0.37517516	0.951	0.868	3.92E-64	NK-T cells	RPL27
6.27E-66	0.64483028	0.583	0.365	1.03E-61	NK-T cells	PABPC1
5.17E-65	0.64300498	0.279	0.105	8.52E-61	NK-T cells	CKLF
5.32E-64	0.58770031	0.399	0.187	8.76E-60	NK-T cells	ARPC5
1.77E-63	0.33915134	0.975	0.93	2.91E-59	NK-T cells	RPL6

1.02E-62	0.61988185	0.416	0.206	1.68E-58	NK-T cells	RAP1B
5.58E-60	0.57742638	0.398	0.195	9.18E-56	NK-T cells	ARF6
2.01E-59	0.3344222	0.976	0.93	3.31E-55	NK-T cells	RPS16
3.58E-57	0.3347816	0.988	0.941	5.90E-53	NK-T cells	RPS13
7.34E-57	0.55553594	0.38	0.183	1.21E-52	NK-T cells	RBM39
9.15E-56	0.27775539	0.992	0.969	1.51E-51	NK-T cells	RPL7
3.88E-55	0.38736036	0.944	0.877	6.39E-51	NK-T cells	RPS5
4.84E-55	0.50044952	0.32	0.141	7.97E-51	NK-T cells	TMEM50A
7.64E-52	0.56075505	0.324	0.148	1.26E-47	NK-T cells	CDC42SE2
3.44E-50	0.50095932	0.471	0.269	5.66E-46	NK-T cells	CAPZB
6.20E-50	0.55232295	0.268	0.113	1.02E-45	NK-T cells	CAPZA1
8.79E-50	0.48618974	0.783	0.623	1.45E-45	NK-T cells	UBC
3.83E-47	0.53778643	0.378	0.2	6.31E-43	NK-T cells	PTP4A2
6.37E-47	0.5132205	0.368	0.189	1.05E-42	NK-T cells	YWHAZ
1.60E-46	0.50994514	0.43	0.237	2.63E-42	NK-T cells	SON
2.03E-46	0.48033487	0.261	0.111	3.34E-42	NK-T cells	YPEL3
7.00E-46	0.51884215	0.312	0.148	1.15E-41	NK-T cells	PDIA3
3.14E-44	0.41745842	0.251	0.105	5.17E-40	NK-T cells	RNF213
6.93E-44	0.49034258	0.44	0.253	1.14E-39	NK-T cells	CNBP
1.10E-43	0.39029666	0.253	0.106	1.81E-39	NK-T cells	VMP1
1.12E-43	0.51594506	0.281	0.131	1.85E-39	NK-T cells	SRSF11
1.99E-43	0.37241683	0.876	0.761	3.29E-39	NK-T cells	RPL4
3.15E-42	0.48238064	0.388	0.211	5.18E-38	NK-T cells	LY6E
4.40E-42	0.26674425	0.958	0.898	7.24E-38	NK-T cells	UBA52
6.25E-42	0.52253091	0.383	0.211	1.03E-37	NK-T cells	TPM3
1.41E-41	0.27263804	0.972	0.923	2.33E-37	NK-T cells	RPS20
3.15E-41	0.46783883	0.353	0.187	5.19E-37	NK-T cells	HNRNPA3
3.72E-41	0.28251477	0.976	0.911	6.12E-37	NK-T cells	RPL37
8.79E-41	0.43812239	0.554	0.357	1.45E-36	NK-T cells	SSR4
9.42E-41	0.39332701	0.725	0.555	1.55E-36	NK-T cells	HNRNPA1
2.38E-38	0.46375959	0.252	0.116	3.91E-34	NK-T cells	ARPC5L
3.27E-37	0.44444899	0.354	0.193	5.39E-33	NK-T cells	RBM3
9.12E-35	0.27780462	0.973	0.936	1.50E-30	NK-T cells	RPL9
6.91E-32	0.26325172	0.932	0.868	1.14E-27	NK-T cells	RPL5
2.55E-31	0.37019853	0.682	0.523	4.19E-27	NK-T cells	HSP90AA1
8.93E-31	0.41074172	0.452	0.293	1.47E-26	NK-T cells	EIF3F
1.12E-30	0.25393013	0.912	0.817	1.84E-26	NK-T cells	RPS11
1.40E-30	0.42428523	0.315	0.175	2.30E-26	NK-T cells	HNRNPA0
2.42E-29	0.39678338	0.309	0.173	3.99E-25	NK-T cells	SELENOF
2.64E-29	0.43508753	0.4	0.251	4.35E-25	NK-T cells	SRSF5
1.87E-28	0.41585447	0.459	0.307	3.07E-24	NK-T cells	PNRC1
4.92E-28	0.44225493	0.316	0.182	8.10E-24	NK-T cells	CTSC
1.03E-27	0.41741019	0.289	0.161	1.70E-23	NK-T cells	ARPC4
2.58E-26	0.28455309	0.572	0.399	4.24E-22	NK-T cells	S100A10
4.85E-26	0.37641284	0.448	0.306	7.99E-22	NK-T cells	HNRNPDL
7.21E-26	0.32592388	0.425	0.274	1.19E-21	NK-T cells	PPIB
1.67E-25	0.37833291	0.573	0.436	2.75E-21	NK-T cells	HNRNPA2B1
4.76E-25	0.38886129	0.293	0.171	7.84E-21	NK-T cells	SNRPB
7.63E-24	0.34559733	0.374	0.237	1.26E-19	NK-T cells	DDX24
7.98E-24	0.39395667	0.586	0.467	1.31E-19	NK-T cells	CLIC1
1.75E-23	0.38560131	0.315	0.189	2.88E-19	NK-T cells	CALR
5.63E-23	0.33462596	0.609	0.464	9.27E-19	NK-T cells	RPL36A
2.13E-22	0.34324468	0.254	0.144	3.51E-18	NK-T cells	MSN
1.47E-21	0.32409465	0.347	0.217	2.42E-17	NK-T cells	TAGLN2
3.64E-21	0.29881216	0.368	0.238	6.00E-17	NK-T cells	C9orf16
9.00E-21	0.31777457	0.34	0.216	1.48E-16	NK-T cells	GTF3A
1.19E-20	0.32803086	0.268	0.159	1.96E-16	NK-T cells	SRSF2
8.69E-20	0.2947975	0.358	0.234	1.43E-15	NK-T cells	TMBIM4

3.14E-19	0.32741992	0.271	0.164	5.18E-15	NK-T cells	XRCC5
4.81E-19	0.34584089	0.398	0.281	7.93E-15	NK-T cells	NAP1L1
2.34E-18	0.33309816	0.271	0.167	3.86E-14	NK-T cells	HNRNPU
1.33E-16	0.28513195	0.605	0.475	2.19E-12	NK-T cells	RPL17
1.55E-16	0.26092846	0.39	0.275	2.56E-12	NK-T cells	ERP29
1.84E-16	0.28970768	0.359	0.249	3.04E-12	NK-T cells	HNRNPF
3.29E-16	0.29683356	0.293	0.191	5.41E-12	NK-T cells	KRTCAP2
4.56E-16	0.31970415	0.291	0.193	7.51E-12	NK-T cells	ARF1
7.47E-16	0.32060525	0.406	0.299	1.23E-11	NK-T cells	PPP1CA
1.48E-15	0.27903529	0.563	0.441	2.44E-11	NK-T cells	CDC42
1.53E-15	0.26854579	0.683	0.568	2.53E-11	NK-T cells	HMGB1
8.44E-15	0.26296512	0.63	0.519	1.39E-10	NK-T cells	PSME1
4.73E-14	0.31593973	0.281	0.19	7.80E-10	NK-T cells	TCF25
5.16E-14	0.25226442	0.385	0.275	8.50E-10	NK-T cells	SPCS2
2.34E-13	0.27532486	0.272	0.183	3.86E-09	NK-T cells	CHMP4A
1.87E-12	0.26239239	0.267	0.181	3.08E-08	NK-T cells	EIF3D
2.43E-12	0.27932203	0.413	0.314	4.01E-08	NK-T cells	HMGN2
4.74E-09	0.27820142	0.281	0.21	7.80E-05	NK-T cells	SRRM1
2.44E-05	0.33238771	0.298	0.248	0.4021621	NK-T cells	TUBA1B
0	4.5005927	0.906	0.01	0	Monocytes	S100A9
0	4.42503665	0.785	0.009	0	Monocytes	S100A8
0	3.68504873	0.848	0.005	0	Monocytes	LYZ
0	3.65481973	0.982	0.014	0	Monocytes	TYROBP
0	3.30330233	0.961	0.051	0	Monocytes	S100A4
0	3.29208179	0.914	0.006	0	Monocytes	AIF1
0	3.28096078	0.924	0.007	0	Monocytes	FCER1G
0	3.11784506	0.899	0.004	0	Monocytes	LST1
0	2.92218629	0.791	0.033	0	Monocytes	FOS
0	2.83500559	0.916	0.047	0	Monocytes	LGALS1
0	2.78093536	0.681	0.022	0	Monocytes	TIMP1
0	2.71942722	0.881	0.03	0	Monocytes	SRGN
0	2.64558197	0.84	0.015	0	Monocytes	CTSS
0	2.64436136	0.997	0.374	0	Monocytes	TMSB4X
0	2.61198214	0.796	0.002	0	Monocytes	FCN1
0	2.50675597	0.623	0.002	0	Monocytes	AC020656.1
0	2.38958606	0.977	0.16	0	Monocytes	S100A6
0	2.34042966	0.833	0.276	0	Monocytes	HLA-DRA
0	2.33955293	0.818	0.06	0	Monocytes	DUSP1
0	2.2651574	0.392	0	0	Monocytes	S100A12
0	2.12650162	0.718	0.084	0	Monocytes	IFITM2
0	2.08743748	0.648	0.007	0	Monocytes	IFI30
0	2.07542391	0.601	0.088	0	Monocytes	HLA-DPB1
0	2.06432266	0.615	0.064	0	Monocytes	HLA-DPA1
0	2.04232354	0.753	0.218	0	Monocytes	HLA-DRB1
0	2.01521404	0.615	0.004	0	Monocytes	CFD
0	1.98573849	0.901	0.279	0	Monocytes	PSAP
0	1.98005755	0.713	0.04	0	Monocytes	EMP3
0	1.9356827	0.954	0.433	0	Monocytes	SH3BGRL3
0	1.86797725	0.428	0.001	0	Monocytes	VCAN
0	1.856046	0.499	0.001	0	Monocytes	SMIM25
0	1.83220337	0.684	0.031	0	Monocytes	GMFG
0	1.81919185	0.482	0.008	0	Monocytes	CD14
0	1.80593774	0.548	0.001	0	Monocytes	MNDA
0	1.80273436	0.972	0.591	0	Monocytes	CST3
0	1.76963416	0.511	0.011	0	Monocytes	RGS2
0	1.75558	0.735	0.112	0	Monocytes	TYMP
0	1.74374334	0.593	0.004	0	Monocytes	SPI1
0	1.7282951	0.657	0.092	0	Monocytes	CEBPB

0	1.71659301	0.903	0.216	0	Monocytes	HLA-B
0	1.71308296	0.952	0.102	0	Monocytes	NEAT1
0	1.69813684	0.609	0.024	0	Monocytes	ITGB2
0	1.69051597	0.588	0.012	0	Monocytes	PYCARD
0	1.67871642	0.566	0.007	0	Monocytes	C1orf162
0	1.66081154	0.587	0.048	0	Monocytes	CD52
0	1.6577134	0.642	0.026	0	Monocytes	LAPTM5
0	1.65434984	0.681	0.118	0	Monocytes	JUNB
0	1.64317256	0.557	0.061	0	Monocytes	NAMPT
0	1.60925829	0.994	0.817	0	Monocytes	ACTB
0	1.58800028	0.997	0.707	0	Monocytes	TMSB10
0	1.5791626	0.523	0.016	0	Monocytes	AP1S2
0	1.57530902	0.421	0.03	0	Monocytes	HLA-DQB1
0	1.54068383	0.522	0.011	0	Monocytes	CARD16
0	1.53875015	0.456	0.001	0	Monocytes	MS4A7
0	1.53645407	0.684	0.16	0	Monocytes	VIM
0	1.53578948	0.43	0.001	0	Monocytes	MS4A6A
0	1.53541258	0.493	0	0	Monocytes	CSTA
0	1.50716926	1	0.818	0	Monocytes	B2M
0	1.50240896	0.572	0.092	0	Monocytes	STXBP2
0	1.48512185	0.599	0.036	0	Monocytes	HCST
0	1.47145344	0.504	0.073	0	Monocytes	IER2
0	1.46994647	0.532	0.035	0	Monocytes	RNASET2
0	1.46336501	0.568	0.078	0	Monocytes	ZFP36
0	1.43363495	0.562	0.03	0	Monocytes	FXYD5
0	1.40633955	0.511	0.053	0	Monocytes	CD68
0	1.40498316	0.74	0.203	0	Monocytes	TSPO
0	1.39770165	0.584	0.048	0	Monocytes	ARHGDIB
0	1.3953564	0.45	0.015	0	Monocytes	CD44
0	1.39097701	0.384	0.018	0	Monocytes	PLAC8
0	1.3840533	0.834	0.257	0	Monocytes	HLA-C
0	1.38230358	0.453	0.01	0	Monocytes	FGL2
0	1.38093299	0.554	0.037	0	Monocytes	CORO1A
0	1.36691396	0.396	0.004	0	Monocytes	PLAUR
0	1.35500156	0.529	0.033	0	Monocytes	PTPRC
0	1.35249364	0.329	0.01	0	Monocytes	FCGR3A
0	1.34184606	0.51	0.026	0	Monocytes	CD37
0	1.34037161	0.401	0.001	0	Monocytes	IGSF6
0	1.33404375	0.561	0.097	0	Monocytes	VMP1
0	1.32799545	0.526	0.071	0	Monocytes	ANXA2
0	1.28894785	0.438	0.023	0	Monocytes	VSIR
0	1.27158113	0.394	0.001	0	Monocytes	CFP
0	1.26129305	0.436	0.061	0	Monocytes	HLA-DMA
0	1.25273304	0.456	0.016	0	Monocytes	LCP1
0	1.24118407	0.351	0.002	0	Monocytes	MAFB
0	1.2405106	0.41	0.003	0	Monocytes	LY96
0	1.23127215	0.457	0.023	0	Monocytes	GPSM3
0	1.22925661	0.421	0.018	0	Monocytes	C4orf48
0	1.22255879	0.989	0.281	0	Monocytes	MALAT1
0	1.21951315	0.374	0.001	0	Monocytes	CYBB
0	1.21563533	0.436	0.039	0	Monocytes	LSP1
0	1.20454899	0.36	0.004	0	Monocytes	MEF2C
0	1.19799653	0.343	0	0	Monocytes	FPR1
0	1.1913533	0.475	0.071	0	Monocytes	PSMB9
0	1.18932833	0.409	0.027	0	Monocytes	ANXA1
0	1.18248375	0.349	0.004	0	Monocytes	JAML
0	1.17404115	0.442	0.023	0	Monocytes	CD48
0	1.16189785	0.369	0.015	0	Monocytes	NUP214

0	1.16171858	0.606	0.127	0	Monocytes	HLA-E
0	1.14130965	0.465	0.046	0	Monocytes	EFHD2
0	1.13301166	0.355	0	0	Monocytes	HCK
0	1.12874043	0.365	0.002	0	Monocytes	TNFSF13B
0	1.12455817	0.398	0.02	0	Monocytes	ZEB2
0	1.12450718	0.323	0.003	0	Monocytes	BCL2A1
0	1.12381276	0.349	0.005	0	Monocytes	FGR
0	1.12098391	0.257	0.01	0	Monocytes	SLC2A3
0	1.09513786	0.343	0.006	0	Monocytes	TNFRSF1B
0	1.08672312	0.322	0.001	0	Monocytes	MPEG1
0	1.08154412	0.341	0.007	0	Monocytes	SAMHD1
0	1.08047671	0.341	0.036	0	Monocytes	DUSP6
0	1.07287795	0.435	0.05	0	Monocytes	FYB1
0	1.06473205	0.376	0.01	0	Monocytes	GLIPR1
0	1.06015786	0.297	0	0	Monocytes	CLEC12A
0	1.05707623	0.374	0.027	0	Monocytes	LIMD2
0	1.04737488	0.407	0.039	0	Monocytes	GNAI2
0	1.04371461	0.326	0.002	0	Monocytes	CLEC7A
0	1.03923134	0.307	0.011	0	Monocytes	MARCKS
0	1.03801597	0.365	0.012	0	Monocytes	EVI2B
0	1.03497307	0.396	0.039	0	Monocytes	RHOG
0	1.03292885	0.374	0.023	0	Monocytes	CD53
0	1.01756565	0.282	0.007	0	Monocytes	PTPRE
0	1.01327347	0.282	0	0	Monocytes	LILRA5
0	0.99857303	0.308	0.01	0	Monocytes	BST2
0	0.99017747	0.285	0.003	0	Monocytes	POU2F2
0	0.97613811	0.311	0.01	0	Monocytes	CASP1
0	0.96906064	0.26	0.013	0	Monocytes	FOSB
0	0.9680746	0.351	0.029	0	Monocytes	CNPY3
0	0.9450514	0.329	0.019	0	Monocytes	UCP2
0	0.93620003	0.319	0.026	0	Monocytes	PTPN6
0	0.92865809	0.293	0	0	Monocytes	LRRC25
0	0.91410317	0.325	0.015	0	Monocytes	MYO1F
0	0.91309729	0.26	0.015	0	Monocytes	CRIP1
0	0.91275519	0.262	0.004	0	Monocytes	LYN
0	0.91089909	0.293	0.011	0	Monocytes	PLEK
0	0.90881371	0.264	0.002	0	Monocytes	LY86
0	0.90434259	0.285	0.013	0	Monocytes	SLC11A1
0	0.90390115	0.323	0.012	0	Monocytes	HCLS1
0	0.90350426	0.287	0.002	0	Monocytes	PILRA
0	0.90197657	0.262	0	0	Monocytes	LILRB2
0	0.88775228	0.254	0.008	0	Monocytes	ASGR1
0	0.882385	0.28	0	0	Monocytes	NCF2
0	0.87839912	0.311	0.027	0	Monocytes	PPT1
0	0.87220639	0.28	0.022	0	Monocytes	LIPA
0	0.84811447	0.272	0.013	0	Monocytes	FLNA
0	0.83517623	0.291	0.015	0	Monocytes	RGS10
0	0.83230389	0.287	0.022	0	Monocytes	GIMAP4
0	0.8280855	0.258	0.003	0	Monocytes	LGALS9
0	0.82775264	0.271	0.017	0	Monocytes	SMAP2
0	0.82675469	0.279	0.01	0	Monocytes	DOK2
0	0.81334011	0.257	0.02	0	Monocytes	KLF2
0	0.81231901	0.253	0	0	Monocytes	CSF1R
0	0.80237677	0.289	0.026	0	Monocytes	ARRB2
0	0.7900583	0.286	0.022	0	Monocytes	TGFB1
6.17E-307	1.54733303	0.802	0.288	1.02E-302	Monocytes	COTL1
1.22E-298	1.31141472	0.956	0.557	2.00E-294	Monocytes	CYBA
1.78E-294	1.21111109	0.616	0.14	2.93E-290	Monocytes	ARPC1B

3.20E-293	0.97502496	0.327	0.038	5.27E-289	Monocytes	CPVL
1.38E-291	1.32870576	0.953	0.526	2.27E-287	Monocytes	S100A11
3.15E-288	0.9767352	0.376	0.051	5.20E-284	Monocytes	WSB1
6.38E-287	0.84837488	0.267	0.025	1.05E-282	Monocytes	WARS
4.82E-286	0.71575771	0.276	0.026	7.95E-282	Monocytes	RAC2
3.93E-275	0.79877512	0.282	0.03	6.48E-271	Monocytes	ZYX
4.18E-260	0.89433045	0.311	0.038	6.89E-256	Monocytes	RNF149
1.17E-259	1.14053983	0.381	0.059	1.93E-255	Monocytes	GLUL
4.24E-252	1.81502431	0.872	0.488	6.98E-248	Monocytes	CD74
3.84E-250	0.81212365	0.28	0.032	6.32E-246	Monocytes	IQGAP1
3.92E-249	0.83548967	0.996	0.924	6.45E-245	Monocytes	RPL28
9.16E-245	0.86891697	0.355	0.053	1.51E-240	Monocytes	FAM49B
1.96E-237	0.89784541	0.983	0.864	3.24E-233	Monocytes	RPS2
1.13E-230	0.71793805	0.272	0.033	1.86E-226	Monocytes	VASP
1.09E-229	1.21716155	0.974	0.776	1.80E-225	Monocytes	SAT1
6.53E-226	0.93718263	0.417	0.078	1.08E-221	Monocytes	CMTM6
2.94E-224	0.77466767	0.989	0.92	4.84E-220	Monocytes	RPS9
3.58E-221	1.00179424	0.993	0.92	5.90E-217	Monocytes	RPS19
1.78E-218	0.75081184	0.282	0.037	2.94E-214	Monocytes	BID
6.71E-218	0.89924802	0.395	0.073	1.11E-213	Monocytes	LRRFIP1
4.34E-216	1.10181232	0.517	0.128	7.15E-212	Monocytes	GRN
3.07E-215	1.17236925	0.471	0.108	5.05E-211	Monocytes	VAMP5
8.00E-214	0.8889196	0.296	0.042	1.32E-209	Monocytes	CCNL1
1.58E-205	0.86055366	0.95	0.762	2.60E-201	Monocytes	OAZ1
1.70E-204	1.14128725	0.286	0.041	2.80E-200	Monocytes	ISG15
1.39E-193	0.99801631	0.738	0.297	2.29E-189	Monocytes	HLA-A
3.02E-185	0.79853612	0.99	0.854	4.97E-181	Monocytes	RPL39
4.68E-185	1.02941226	0.409	0.091	7.71E-181	Monocytes	MCL1
1.45E-173	1.0248857	0.301	0.053	2.38E-169	Monocytes	HLA-DRB5
7.06E-173	1.01259711	0.568	0.183	1.16E-168	Monocytes	ARPC5
2.01E-170	1.22895674	0.414	0.1	3.31E-166	Monocytes	HSPA1A
1.65E-163	1.01768565	0.59	0.201	2.72E-159	Monocytes	BTG1
1.02E-162	0.5632531	0.994	0.955	1.67E-158	Monocytes	RPLP2
1.04E-159	0.96175613	0.409	0.101	1.72E-155	Monocytes	CTSZ
5.59E-158	1.00912531	0.319	0.064	9.20E-154	Monocytes	HSPA1B
6.47E-158	1.0498736	0.591	0.206	1.07E-153	Monocytes	TXNIP
1.23E-156	0.92950053	0.901	0.602	2.03E-152	Monocytes	H3F3B
1.93E-152	0.90195363	0.36	0.082	3.18E-148	Monocytes	JUND
5.51E-151	0.69548449	0.308	0.06	9.08E-147	Monocytes	RNF130
1.38E-150	0.71532158	0.253	0.042	2.28E-146	Monocytes	IRF1
1.94E-146	1.12334938	0.506	0.162	3.19E-142	Monocytes	ZFP36L1
4.40E-145	0.55516043	0.985	0.933	7.24E-141	Monocytes	RPL18A
1.14E-140	0.97926457	0.698	0.347	1.88E-136	Monocytes	ARPC2
3.27E-140	0.66681225	0.967	0.813	5.38E-136	Monocytes	H3F3A
2.56E-139	0.90708013	0.851	0.578	4.21E-135	Monocytes	NPC2
1.56E-137	0.7259715	0.932	0.774	2.57E-133	Monocytes	PFN1
3.07E-137	0.5691321	0.982	0.897	5.05E-133	Monocytes	UBA52
1.21E-136	0.85232068	0.354	0.085	1.99E-132	Monocytes	GCA
2.58E-134	0.48359608	0.999	0.977	4.25E-130	Monocytes	RPS14
1.01E-132	0.46586131	0.999	0.99	1.66E-128	Monocytes	RPLP1
5.30E-132	0.55992194	0.997	0.97	8.73E-128	Monocytes	RPS27
8.03E-128	0.79002409	0.416	0.12	1.32E-123	Monocytes	H2AFY
8.38E-128	0.44927999	0.999	0.991	1.38E-123	Monocytes	RPL10
4.08E-127	0.79878163	0.366	0.095	6.71E-123	Monocytes	LGALS3
7.12E-126	0.53916053	0.978	0.903	1.17E-121	Monocytes	FAU
2.66E-123	0.80557804	0.406	0.117	4.38E-119	Monocytes	ACTR2
1.93E-122	0.57269401	0.258	0.051	3.18E-118	Monocytes	SYNGR2
3.20E-117	0.88199105	0.402	0.12	5.28E-113	Monocytes	KLF6

3.99E-117	0.61621059	0.268	0.057	6.58E-113	Monocytes	ATP1B3
1.83E-116	0.78127731	0.494	0.174	3.02E-112	Monocytes	CAP1
3.78E-116	0.80106181	0.37	0.104	6.22E-112	Monocytes	CKLF
7.45E-116	0.50504921	0.971	0.931	1.23E-111	Monocytes	RPL27A
1.66E-113	0.63816286	0.273	0.061	2.73E-109	Monocytes	TPP1
1.56E-112	0.82037586	0.642	0.294	2.57E-108	Monocytes	SERP1
7.47E-111	0.60638381	0.272	0.061	1.23E-106	Monocytes	IFNGR2
5.96E-109	0.54709753	0.965	0.843	9.82E-105	Monocytes	PFDN5
1.58E-108	0.46771515	0.994	0.958	2.60E-104	Monocytes	RPS15A
9.82E-107	1.23402034	0.684	0.386	1.62E-102	Monocytes	IFITM3
1.14E-105	0.7504771	0.329	0.088	1.88E-101	Monocytes	JUN
6.97E-105	0.87018096	0.731	0.395	1.15E-100	Monocytes	S100A10
3.68E-100	0.80292312	0.539	0.226	6.07E-96	Monocytes	SDCBP
3.84E-97	0.80518971	0.526	0.22	6.33E-93	Monocytes	TKT
7.61E-97	0.61122146	0.873	0.705	1.25E-92	Monocytes	CFL1
2.49E-96	0.55111981	0.258	0.061	4.11E-92	Monocytes	CASP4
8.46E-96	0.42914312	0.985	0.936	1.39E-91	Monocytes	RPS24
2.15E-95	0.6385519	0.363	0.111	3.55E-91	Monocytes	ARL6IP5
2.81E-94	0.68264466	0.349	0.107	4.63E-90	Monocytes	MTPN
3.22E-93	0.75345655	0.67	0.364	5.30E-89	Monocytes	PABPC1
2.05E-90	0.62068306	0.356	0.111	3.38E-86	Monocytes	CAPZA1
3.91E-90	0.71072323	0.64	0.315	6.45E-86	Monocytes	DDX5
1.08E-86	0.62867192	0.28	0.076	1.78E-82	Monocytes	TSC22D3
2.11E-85	0.73861379	0.514	0.225	3.47E-81	Monocytes	ATP6V0B
4.62E-85	0.63940571	0.326	0.1	7.61E-81	Monocytes	SERPINB1
9.02E-85	0.67184792	0.76	0.458	1.49E-80	Monocytes	LAMTOR4
1.18E-81	0.64561658	0.254	0.068	1.95E-77	Monocytes	UBE2D1
2.44E-80	0.39394681	0.949	0.848	4.02E-76	Monocytes	RPS3
3.03E-78	0.62617289	0.773	0.56	4.98E-74	Monocytes	ARPC3
4.33E-78	0.50889411	0.682	0.359	7.13E-74	Monocytes	SERPINA1
3.28E-77	0.57879481	0.287	0.086	5.40E-73	Monocytes	TRA2B
8.33E-77	0.57629983	0.265	0.075	1.37E-72	Monocytes	TUBA1A
6.14E-75	0.62944539	0.457	0.187	1.01E-70	Monocytes	YWHAZ
2.29E-74	0.64618303	0.604	0.31	3.77E-70	Monocytes	FKBP1A
3.73E-74	0.6922626	0.337	0.116	6.15E-70	Monocytes	ZFP36L2
9.07E-74	0.68675819	0.688	0.416	1.49E-69	Monocytes	ACTG1
1.01E-73	0.61790816	0.338	0.116	1.66E-69	Monocytes	MTDH
1.47E-72	0.53723922	0.265	0.078	2.43E-68	Monocytes	SMCO4
6.10E-72	0.67583934	0.474	0.21	1.00E-67	Monocytes	TPM3
5.54E-70	0.68683715	0.605	0.337	9.12E-66	Monocytes	RHOA
1.26E-68	0.44812973	0.932	0.818	2.08E-64	Monocytes	RPL18
5.57E-68	0.71759659	0.54	0.277	9.17E-64	Monocytes	NAP1L1
2.53E-67	0.29336413	0.999	0.989	4.17E-63	Monocytes	RPL13
3.16E-67	0.54332443	0.311	0.106	5.20E-63	Monocytes	PSMB8
3.47E-67	0.65853988	0.468	0.215	5.71E-63	Monocytes	ANXA5
3.92E-67	0.57094866	0.407	0.165	6.46E-63	Monocytes	ACTR3
5.75E-66	0.5972245	0.395	0.159	9.47E-62	Monocytes	ARPC4
6.10E-66	0.42867764	0.971	0.892	1.00E-61	Monocytes	RPS29
9.34E-66	0.29837686	0.996	0.982	1.54E-61	Monocytes	RPS15
2.67E-64	0.80518032	0.552	0.308	4.39E-60	Monocytes	CEBPD
4.89E-64	0.28833727	0.999	0.98	8.06E-60	Monocytes	RPS18
1.42E-63	0.39809386	0.953	0.897	2.34E-59	Monocytes	PTMA
4.81E-63	0.36019451	0.974	0.93	7.92E-59	Monocytes	RPS16
9.20E-63	0.28453514	0.997	0.986	1.51E-58	Monocytes	RPL32
2.31E-61	0.44716888	0.269	0.087	3.80E-57	Monocytes	OSTF1
1.80E-60	0.59717838	0.535	0.27	2.96E-56	Monocytes	FCGRT
4.44E-60	0.50024274	0.273	0.091	7.31E-56	Monocytes	PSMB10
1.32E-59	0.58551506	0.355	0.141	2.18E-55	Monocytes	MSN

2.23E-57	0.35210829	0.983	0.955	3.68E-53	Monocytes	RPS8
3.42E-56	0.51035682	0.814	0.634	5.63E-52	Monocytes	CALM2
3.51E-56	0.30332036	0.997	0.98	5.77E-52	Monocytes	RPS28
5.71E-53	0.32639807	0.988	0.955	9.41E-49	Monocytes	RPL21
1.85E-52	0.28134951	0.989	0.973	3.04E-48	Monocytes	RPS12
3.34E-52	0.26748292	0.996	0.976	5.50E-48	Monocytes	RPL11
3.99E-52	0.3478637	0.957	0.923	6.58E-48	Monocytes	EIF1
8.57E-50	0.38122034	0.932	0.817	1.41E-45	Monocytes	RPS11
5.25E-49	0.56856011	0.503	0.277	8.65E-45	Monocytes	ASAH1
9.46E-49	0.52412252	0.456	0.23	1.56E-44	Monocytes	SH3BGRL
9.82E-49	0.4162135	1	0.997	1.62E-44	Monocytes	FTH1
2.52E-48	0.5437472	0.609	0.375	4.15E-44	Monocytes	AP2S1
3.34E-47	0.4542628	0.322	0.132	5.50E-43	Monocytes	N4BP2L2
7.46E-47	0.4707388	0.334	0.142	1.23E-42	Monocytes	TMEM50A
6.19E-46	0.45925125	0.307	0.124	1.02E-41	Monocytes	IFI27L2
8.81E-45	0.40324966	0.913	0.763	1.45E-40	Monocytes	EEF1D
9.59E-45	0.50309913	0.275	0.109	1.58E-40	Monocytes	DDX3X
1.43E-43	0.51994136	0.359	0.165	2.35E-39	Monocytes	HNRNPU
4.98E-43	0.51196346	0.395	0.193	8.20E-39	Monocytes	RBM3
5.28E-43	0.50357886	0.445	0.232	8.70E-39	Monocytes	TMBIM4
6.73E-43	0.29647346	0.993	0.942	1.11E-38	Monocytes	ATP5F1E
7.29E-43	0.51432822	0.664	0.439	1.20E-38	Monocytes	CDC42
1.06E-42	0.50056357	0.518	0.295	1.74E-38	Monocytes	DAZAP2
1.54E-42	0.29859508	0.978	0.942	2.54E-38	Monocytes	RPS13
2.26E-41	0.85991621	0.485	0.277	3.72E-37	Monocytes	CTSD
2.63E-40	0.36663711	0.872	0.707	4.34E-36	Monocytes	RPS21
5.28E-39	0.52580726	0.39	0.201	8.70E-35	Monocytes	PTP4A2
1.67E-38	0.29682967	0.938	0.877	2.75E-34	Monocytes	RPL30
7.71E-38	0.48706941	0.546	0.327	1.27E-33	Monocytes	ZFAS1
2.67E-37	0.38238363	0.256	0.104	4.40E-33	Monocytes	ATG3
1.06E-36	0.57500169	0.359	0.182	1.75E-32	Monocytes	CTSC
1.10E-36	0.39821153	0.293	0.13	1.81E-32	Monocytes	GNB2
3.46E-36	0.44275976	0.291	0.13	5.71E-32	Monocytes	LSM8
1.86E-35	0.25579859	0.981	0.936	3.07E-31	Monocytes	RPL36
3.29E-34	0.39021727	0.254	0.108	5.42E-30	Monocytes	SCAF11
2.45E-32	0.42927368	0.282	0.132	4.04E-28	Monocytes	LSM6
8.40E-32	0.26965724	0.977	0.911	1.38E-27	Monocytes	RPL37
2.85E-31	0.69765198	0.32	0.162	4.69E-27	Monocytes	HMOX1
9.93E-31	0.39306258	0.358	0.185	1.63E-26	Monocytes	RBM39
1.63E-30	0.44473445	0.298	0.145	2.68E-26	Monocytes	ZFAND5
5.44E-30	0.42799557	0.616	0.436	8.96E-26	Monocytes	PRELID1
6.07E-30	0.38467615	0.32	0.161	1.00E-25	Monocytes	SFT2D1
6.22E-30	0.39141967	0.645	0.466	1.03E-25	Monocytes	CLIC1
4.64E-29	0.36622464	0.412	0.23	7.64E-25	Monocytes	PKM
2.21E-28	0.25940887	0.927	0.87	3.63E-24	Monocytes	RPL27
3.17E-28	0.52967662	0.269	0.132	5.23E-24	Monocytes	NFKBIA
6.09E-28	0.6460742	0.453	0.285	1.00E-23	Monocytes	CTSB
1.50E-27	0.4107892	0.36	0.198	2.48E-23	Monocytes	ARF6
2.16E-27	0.39798746	0.489	0.307	3.56E-23	Monocytes	PNRC1
4.12E-27	0.54043002	0.568	0.401	6.79E-23	Monocytes	CSTB
3.60E-26	0.33287359	0.728	0.558	5.94E-22	Monocytes	RAC1
6.88E-26	0.39048405	0.435	0.265	1.13E-21	Monocytes	DRAP1
1.22E-25	0.35521831	0.405	0.235	2.01E-21	Monocytes	APLP2
6.33E-25	0.35037503	0.262	0.129	1.04E-20	Monocytes	ANP32B
1.13E-24	0.32761693	0.449	0.272	1.86E-20	Monocytes	CAPZB
2.08E-24	0.32640191	0.261	0.129	3.43E-20	Monocytes	ATP6AP2
3.58E-24	0.37116939	0.273	0.14	5.90E-20	Monocytes	LITAF
1.16E-23	0.35758394	0.37	0.216	1.91E-19	Monocytes	PGLS

2.25E-23	0.32741004	0.36	0.204	3.71E-19	Monocytes	GDI2
4.32E-23	0.42274344	0.374	0.224	7.12E-19	Monocytes	TMEM167A
1.09E-22	0.39316786	0.273	0.144	1.79E-18	Monocytes	DDX21
6.51E-22	0.33161743	0.296	0.159	1.07E-17	Monocytes	RAP1A
1.48E-21	0.30834294	0.482	0.312	2.45E-17	Monocytes	HMGN2
1.50E-21	0.46487523	0.417	0.272	2.46E-17	Monocytes	RHOB
2.48E-21	0.40237144	0.398	0.246	4.09E-17	Monocytes	TUBA1B
4.80E-21	0.2883175	0.286	0.154	7.91E-17	Monocytes	CAPNS1
5.79E-21	0.41492093	0.503	0.35	9.53E-17	Monocytes	PSME2
1.48E-20	0.42930866	0.351	0.214	2.43E-16	Monocytes	LY6E
2.90E-20	0.26735065	0.744	0.596	4.77E-16	Monocytes	RPS10
3.81E-20	0.33190252	0.352	0.21	6.28E-16	Monocytes	RAP1B
3.10E-19	0.29207492	0.26	0.138	5.11E-15	Monocytes	WDR1
1.21E-18	0.39185694	0.351	0.218	1.99E-14	Monocytes	TAGLN2
2.37E-18	0.29099161	0.42	0.269	3.90E-14	Monocytes	SSR2
4.41E-18	0.33714792	0.612	0.482	7.27E-14	Monocytes	BRI3
8.82E-18	0.26864007	0.633	0.464	1.45E-13	Monocytes	RPL36A
1.48E-17	0.30856091	0.482	0.331	2.44E-13	Monocytes	SEC11A
2.32E-17	0.25661993	0.358	0.22	3.82E-13	Monocytes	LSM7
4.17E-17	0.39428092	0.633	0.511	6.87E-13	Monocytes	CD63
4.34E-17	0.31187978	0.644	0.502	7.15E-13	Monocytes	NOP10
4.73E-17	0.29319019	0.293	0.171	7.80E-13	Monocytes	CAST
2.06E-16	0.25415311	0.262	0.148	3.39E-12	Monocytes	DEK
6.90E-16	0.29256671	0.311	0.19	1.14E-11	Monocytes	HNRNPA3
7.49E-16	0.27515454	0.309	0.187	1.23E-11	Monocytes	MOB1A
4.51E-15	0.32099168	0.301	0.187	7.43E-11	Monocytes	POLE4
5.33E-13	0.2712894	0.394	0.268	8.77E-09	Monocytes	PET100
4.41E-12	0.29051048	0.326	0.218	7.27E-08	Monocytes	PPP1CB
1.12E-11	0.25449362	0.367	0.254	1.84E-07	Monocytes	SRSF5
1.17E-10	0.35362749	0.26	0.172	1.93E-06	Monocytes	CAPG
2.49E-282	1.32009592	0.686	0.162	4.10E-278	Glomerular parietal epithelial cells	VIM
1.46E-255	1.26397832	0.567	0.116	2.41E-251	Glomerular parietal epithelial cells	SOX4
7.17E-225	1.12243197	0.971	0.527	1.18E-220	Glomerular parietal epithelial cells	S100A11
3.97E-222	1.09295803	0.668	0.172	6.54E-218	Glomerular parietal epithelial cells	S100A6
1.64E-202	1.05723602	0.992	0.708	2.70E-198	Glomerular parietal epithelial cells	TMSB10
6.41E-202	0.87152816	0.419	0.076	1.06E-197	Glomerular parietal epithelial cells	ANXA2
2.23E-192	0.41866668	0.913	0.379	3.67E-188	Glomerular parietal epithelial cells	TMSB4X
8.76E-190	1.18475738	0.754	0.277	1.44E-185	Glomerular parietal epithelial cells	TPM1
3.24E-187	0.98756938	0.992	0.785	5.34E-183	Glomerular parietal epithelial cells	IL32
1.56E-165	1.00183392	0.841	0.393	2.56E-161	Glomerular parietal epithelial cells	S100A10
7.67E-153	1.12331745	0.933	0.617	1.26E-148	Glomerular parietal epithelial cells	GSTP1
1.59E-146	0.81857261	0.275	0.045	2.61E-142	Glomerular parietal epithelial cells	CCL2
5.30E-142	0.67764631	0.986	0.871	8.73E-138	Glomerular parietal epithelial cells	MYL6
1.72E-141	0.92207233	0.832	0.413	2.84E-137	Glomerular parietal epithelial cells	ACTG1
2.69E-136	0.81894264	0.619	0.212	4.43E-132	Glomerular parietal epithelial cells	TAGLN2
7.83E-128	0.92272546	0.81	0.402	1.29E-123	Glomerular parietal epithelial cells	KRT8
1.34E-124	0.63444297	0.305	0.061	2.20E-120	Glomerular parietal epithelial cells	AL353751.1
8.92E-121	0.74807373	0.405	0.106	1.47E-116	Glomerular parietal epithelial cells	PDLIM1
2.19E-119	0.82061183	0.905	0.592	3.61E-115	Glomerular parietal epithelial cells	ENO1
1.39E-118	0.89886644	0.813	0.431	2.28E-114	Glomerular parietal epithelial cells	KRT18
1.71E-118	1.06969671	0.7	0.322	2.81E-114	Glomerular parietal epithelial cells	SOD2
1.00E-114	0.79895437	0.716	0.315	1.65E-110	Glomerular parietal epithelial cells	ANXA4
2.36E-112	0.57131514	0.965	0.774	3.89E-108	Glomerular parietal epithelial cells	PFN1
1.59E-111	0.69433354	0.348	0.085	2.62E-107	Glomerular parietal epithelial cells	AKAP12
1.16E-109	0.69385293	0.537	0.182	1.91E-105	Glomerular parietal epithelial cells	TAX1BP3
1.03E-106	0.73842058	0.343	0.085	1.70E-102	Glomerular parietal epithelial cells	WFDC2
1.90E-105	0.7756237	0.737	0.353	3.12E-101	Glomerular parietal epithelial cells	CARHSP1
3.37E-105	0.52037023	0.989	0.896	5.54E-101	Glomerular parietal epithelial cells	PTMA

4.02E-101	0.70462661	0.79	0.384	6.61E-97	Glomerular parietal epithelial cells	IFITM3
6.58E-100	0.6644981	0.348	0.091	1.08E-95	Glomerular parietal epithelial cells	CITED4
2.60E-99	0.68216245	0.829	0.478	4.28E-95	Glomerular parietal epithelial cells	C12orf75
3.25E-99	0.39808968	0.997	0.972	5.35E-95	Glomerular parietal epithelial cells	RPL3
6.86E-99	0.59778349	0.341	0.088	1.13E-94	Glomerular parietal epithelial cells	PLSCR1
3.53E-96	0.37224683	1	0.991	5.81E-92	Glomerular parietal epithelial cells	RPL10
5.17E-94	0.61737376	0.44	0.142	8.51E-90	Glomerular parietal epithelial cells	YWHAH
7.90E-90	0.74705018	0.543	0.212	1.30E-85	Glomerular parietal epithelial cells	STMN1
4.94E-89	0.34164814	0.997	0.989	8.14E-85	Glomerular parietal epithelial cells	RPL13A
6.40E-88	0.65703352	0.533	0.205	1.05E-83	Glomerular parietal epithelial cells	JPT1
9.34E-88	0.61376915	0.857	0.537	1.54E-83	Glomerular parietal epithelial cells	MYL12A
1.83E-85	0.61282793	0.292	0.075	3.02E-81	Glomerular parietal epithelial cells	TUBA1A
5.66E-85	0.46115282	0.257	0.06	9.33E-81	Glomerular parietal epithelial cells	DCDC2
7.68E-85	0.6169269	0.621	0.264	1.27E-80	Glomerular parietal epithelial cells	TCEAL9
2.27E-84	0.55957727	0.286	0.072	3.74E-80	Glomerular parietal epithelial cells	AKR1B1
1.59E-82	0.37222218	1	0.996	2.62E-78	Glomerular parietal epithelial cells	EEF1A1
1.48E-80	0.62722881	0.746	0.387	2.44E-76	Glomerular parietal epithelial cells	CD24
1.53E-80	0.63819812	0.735	0.379	2.52E-76	Glomerular parietal epithelial cells	S100A13
2.18E-79	0.52160571	0.911	0.727	3.59E-75	Glomerular parietal epithelial cells	MYL12B
3.67E-79	0.38396527	0.987	0.933	6.04E-75	Glomerular parietal epithelial cells	RPL18A
1.10E-78	0.39146199	0.987	0.94	1.82E-74	Glomerular parietal epithelial cells	RPL15
7.68E-78	0.49031911	0.96	0.818	1.27E-73	Glomerular parietal epithelial cells	RPLP0
3.43E-77	0.32382481	0.995	0.981	5.64E-73	Glomerular parietal epithelial cells	RPS18
1.18E-76	0.41199692	0.992	0.955	1.94E-72	Glomerular parietal epithelial cells	RPS8
3.40E-76	0.48892782	0.268	0.069	5.60E-72	Glomerular parietal epithelial cells	NMT2
2.10E-75	0.62123979	0.817	0.554	3.45E-71	Glomerular parietal epithelial cells	HNRNPA1
3.22E-74	0.52809723	0.998	0.899	5.31E-70	Glomerular parietal epithelial cells	CRYAB
1.14E-73	0.56762541	0.878	0.633	1.88E-69	Glomerular parietal epithelial cells	CALM2
2.96E-73	0.4347988	0.96	0.819	4.88E-69	Glomerular parietal epithelial cells	ACTB
4.62E-72	0.65263856	0.744	0.43	7.60E-68	Glomerular parietal epithelial cells	FABP3
9.45E-72	0.5589985	0.421	0.148	1.56E-67	Glomerular parietal epithelial cells	SELENOM
2.67E-71	0.34089515	0.99	0.951	4.40E-67	Glomerular parietal epithelial cells	RPL8
1.45E-70	0.45270639	0.251	0.065	2.39E-66	Glomerular parietal epithelial cells	AKR1C1
1.49E-70	0.56541463	0.541	0.227	2.46E-66	Glomerular parietal epithelial cells	SDCBP
2.53E-70	0.63563516	0.695	0.38	4.16E-66	Glomerular parietal epithelial cells	CNN3
9.37E-70	0.63222346	0.687	0.37	1.54E-65	Glomerular parietal epithelial cells	RAN
1.62E-69	0.45747837	0.935	0.758	2.66E-65	Glomerular parietal epithelial cells	PPIA
1.14E-68	0.40522303	0.983	0.885	1.87E-64	Glomerular parietal epithelial cells	RPL10A
3.34E-68	0.56857344	0.589	0.273	5.51E-64	Glomerular parietal epithelial cells	YWHAQ
6.91E-68	0.49454842	0.505	0.203	1.14E-63	Glomerular parietal epithelial cells	SRI
6.33E-67	0.4850625	0.303	0.092	1.04E-62	Glomerular parietal epithelial cells	TMEM54
9.08E-67	0.52181251	0.478	0.192	1.50E-62	Glomerular parietal epithelial cells	NME1
1.13E-66	0.47134697	0.413	0.148	1.86E-62	Glomerular parietal epithelial cells	ARPC1B
2.58E-64	0.5093622	0.36	0.124	4.25E-60	Glomerular parietal epithelial cells	IFI27L2
1.33E-63	0.4081556	0.994	0.963	2.19E-59	Glomerular parietal epithelial cells	RPL12
5.64E-63	0.54804964	0.502	0.216	9.29E-59	Glomerular parietal epithelial cells	ANXA5
8.93E-62	0.55437372	0.414	0.161	1.47E-57	Glomerular parietal epithelial cells	TSC22D1
4.53E-61	0.55738575	0.621	0.311	7.47E-57	Glomerular parietal epithelial cells	FKBP1A
5.08E-61	0.52663379	0.825	0.583	8.37E-57	Glomerular parietal epithelial cells	NPM1
2.86E-60	0.47253166	0.349	0.122	4.72E-56	Glomerular parietal epithelial cells	NIPSNAP2
4.32E-60	0.32400293	0.998	0.969	7.11E-56	Glomerular parietal epithelial cells	RPL7
2.92E-59	0.25762032	0.998	0.989	4.82E-55	Glomerular parietal epithelial cells	RPL13
7.23E-59	0.5627012	0.76	0.463	1.19E-54	Glomerular parietal epithelial cells	CLIC1
7.51E-59	0.63654116	0.517	0.243	1.24E-54	Glomerular parietal epithelial cells	TUBB
1.23E-58	0.3510894	0.971	0.865	2.02E-54	Glomerular parietal epithelial cells	RPS2
7.32E-58	0.49654234	0.411	0.162	1.21E-53	Glomerular parietal epithelial cells	S100A16
4.69E-57	0.41561342	0.351	0.125	7.73E-53	Glomerular parietal epithelial cells	TYMP
8.39E-57	0.44845592	0.944	0.805	1.38E-52	Glomerular parietal epithelial cells	HSPB1

3.32E-56	0.32832011	0.992	0.964	5.47E-52	Glomerular parietal epithelial cells	RPL26
5.01E-56	0.37002216	0.978	0.889	8.25E-52	Glomerular parietal epithelial cells	RPL7A
8.10E-56	0.53491626	0.735	0.441	1.33E-51	Glomerular parietal epithelial cells	HSBP1
2.62E-55	0.31407589	0.981	0.921	4.32E-51	Glomerular parietal epithelial cells	RPS9
7.19E-55	0.30999075	0.962	0.848	1.18E-50	Glomerular parietal epithelial cells	RPS3
8.76E-55	0.39992343	0.333	0.118	1.44E-50	Glomerular parietal epithelial cells	RRAS2
3.18E-54	0.26126891	1	0.99	5.23E-50	Glomerular parietal epithelial cells	RPLP1
1.03E-53	0.52742346	0.69	0.414	1.70E-49	Glomerular parietal epithelial cells	EIF6
1.48E-53	0.36326667	0.968	0.87	2.44E-49	Glomerular parietal epithelial cells	RACK1
1.63E-53	0.29503635	0.995	0.973	2.68E-49	Glomerular parietal epithelial cells	RPS12
2.60E-53	0.5036913	0.851	0.623	4.28E-49	Glomerular parietal epithelial cells	UBC
1.00E-52	0.48764952	0.627	0.324	1.65E-48	Glomerular parietal epithelial cells	HCFC1R1
4.58E-52	0.50957315	0.698	0.398	7.54E-48	Glomerular parietal epithelial cells	CSTB
9.25E-52	0.51669065	0.706	0.416	1.52E-47	Glomerular parietal epithelial cells	PPA1
4.59E-50	0.32763366	0.971	0.867	7.55E-46	Glomerular parietal epithelial cells	RPL5
8.09E-50	0.29598477	0.979	0.939	1.33E-45	Glomerular parietal epithelial cells	RPL29
1.13E-49	0.45264079	0.406	0.169	1.86E-45	Glomerular parietal epithelial cells	CAPG
4.47E-49	0.46331345	0.824	0.606	7.36E-45	Glomerular parietal epithelial cells	H3F3B
4.69E-49	0.3931507	0.897	0.705	7.72E-45	Glomerular parietal epithelial cells	CFL1
1.39E-48	0.30462149	0.983	0.936	2.29E-44	Glomerular parietal epithelial cells	RPS24
1.74E-48	0.27043098	0.968	0.921	2.87E-44	Glomerular parietal epithelial cells	RPS19
1.40E-47	0.43444796	0.838	0.594	2.30E-43	Glomerular parietal epithelial cells	RPS10
2.32E-47	0.41472132	0.456	0.203	3.81E-43	Glomerular parietal epithelial cells	RAB13
2.93E-47	0.29476764	0.979	0.924	4.82E-43	Glomerular parietal epithelial cells	RPL28
3.82E-47	0.39780757	0.344	0.134	6.29E-43	Glomerular parietal epithelial cells	LMNA
6.07E-46	0.42846725	0.424	0.187	1.00E-41	Glomerular parietal epithelial cells	HIF1A
6.54E-46	0.41293065	0.486	0.229	1.08E-41	Glomerular parietal epithelial cells	PKM
7.85E-46	0.45439145	0.494	0.24	1.29E-41	Glomerular parietal epithelial cells	YBX3
5.60E-45	0.43708326	0.462	0.216	9.23E-41	Glomerular parietal epithelial cells	EIF5B
9.27E-45	0.27865152	0.987	0.946	1.53E-40	Glomerular parietal epithelial cells	RPL35
1.15E-44	0.45209003	0.448	0.209	1.90E-40	Glomerular parietal epithelial cells	AC245595.1
1.49E-44	0.39873661	0.314	0.12	2.46E-40	Glomerular parietal epithelial cells	TNFRSF12A
1.59E-44	0.44373108	0.424	0.193	2.63E-40	Glomerular parietal epithelial cells	RBM3
2.29E-44	0.44738352	0.754	0.497	3.77E-40	Glomerular parietal epithelial cells	RHOC
1.38E-42	0.42669713	0.697	0.421	2.28E-38	Glomerular parietal epithelial cells	CAPZA2
2.94E-42	0.44774924	0.549	0.288	4.84E-38	Glomerular parietal epithelial cells	CDK2AP2
3.75E-42	0.41666975	0.81	0.556	6.18E-38	Glomerular parietal epithelial cells	RAC1
5.11E-42	0.28198648	0.976	0.934	8.42E-38	Glomerular parietal epithelial cells	RPL14
6.27E-42	0.37286425	0.278	0.103	1.03E-37	Glomerular parietal epithelial cells	IMPDH2
8.33E-42	0.41186864	0.275	0.102	1.37E-37	Glomerular parietal epithelial cells	JAK1
8.77E-42	0.38109293	0.921	0.729	1.44E-37	Glomerular parietal epithelial cells	PPDPF
2.03E-41	0.40590803	0.622	0.348	3.34E-37	Glomerular parietal epithelial cells	PSME2
3.53E-41	0.37661508	0.876	0.701	5.81E-37	Glomerular parietal epithelial cells	RPSA
3.63E-41	0.34502086	0.324	0.13	5.98E-37	Glomerular parietal epithelial cells	MAP1LC3A
4.41E-41	0.27711357	0.989	0.956	7.27E-37	Glomerular parietal epithelial cells	RPL21
3.45E-40	0.39605877	0.814	0.584	5.68E-36	Glomerular parietal epithelial cells	SUMO2
8.37E-40	0.2748158	0.99	0.958	1.38E-35	Glomerular parietal epithelial cells	RPL31
1.32E-39	0.37676544	0.451	0.214	2.18E-35	Glomerular parietal epithelial cells	TSPO
2.21E-39	0.42482952	0.514	0.268	3.63E-35	Glomerular parietal epithelial cells	MORF4L2
2.31E-39	0.39505505	0.429	0.202	3.80E-35	Glomerular parietal epithelial cells	AIF1L
4.72E-39	0.40119126	0.422	0.199	7.78E-35	Glomerular parietal epithelial cells	MEAF6
4.84E-39	0.4201135	0.717	0.463	7.96E-35	Glomerular parietal epithelial cells	RPL36A
5.83E-39	0.30079947	0.941	0.815	9.60E-35	Glomerular parietal epithelial cells	H3F3A
1.43E-38	0.34440924	0.281	0.108	2.36E-34	Glomerular parietal epithelial cells	PSMB8
3.39E-38	0.25183463	0.994	0.962	5.58E-34	Glomerular parietal epithelial cells	RPS3A
8.08E-38	0.37835271	0.744	0.479	1.33E-33	Glomerular parietal epithelial cells	DYNLT1
6.07E-37	0.46166982	0.47	0.244	1.00E-32	Glomerular parietal epithelial cells	TUBA1B
1.22E-36	0.38898298	0.486	0.246	2.00E-32	Glomerular parietal epithelial cells	DYNC1I2

3.56E-36	0.40488697	0.51	0.27	5.87E-32	Glomerular parietal epithelial cells	RHOB
3.59E-36	0.40852018	0.546	0.3	5.91E-32	Glomerular parietal epithelial cells	SQSTM1
4.70E-36	0.37222851	0.511	0.269	7.75E-32	Glomerular parietal epithelial cells	RAB11A
8.12E-36	0.28691288	0.978	0.851	1.34E-31	Glomerular parietal epithelial cells	SRP14
1.01E-35	0.44227856	0.548	0.31	1.67E-31	Glomerular parietal epithelial cells	ALDH1A1
2.64E-35	0.39200851	0.721	0.476	4.35E-31	Glomerular parietal epithelial cells	HMGN1
4.57E-35	0.34170872	0.387	0.181	7.52E-31	Glomerular parietal epithelial cells	APEX1
1.15E-34	0.38380287	0.525	0.287	1.90E-30	Glomerular parietal epithelial cells	RHEB
1.87E-34	0.37479215	0.76	0.517	3.09E-30	Glomerular parietal epithelial cells	PSME1
2.17E-34	0.35081234	0.852	0.663	3.57E-30	Glomerular parietal epithelial cells	YBX1
1.44E-33	0.2910613	0.954	0.856	2.37E-29	Glomerular parietal epithelial cells	RPL39
1.78E-33	0.29976375	0.305	0.13	2.93E-29	Glomerular parietal epithelial cells	DCTN2
2.83E-33	0.34643194	0.476	0.249	4.67E-29	Glomerular parietal epithelial cells	MRFAP1
2.84E-33	0.35618447	0.397	0.193	4.68E-29	Glomerular parietal epithelial cells	CHMP4B
3.27E-33	0.39748455	0.305	0.132	5.38E-29	Glomerular parietal epithelial cells	NFKBIA
3.91E-33	0.30723073	0.327	0.143	6.44E-29	Glomerular parietal epithelial cells	ELF3
3.96E-33	0.35079935	0.359	0.167	6.53E-29	Glomerular parietal epithelial cells	ACTR3
5.69E-33	0.30503512	0.321	0.14	9.38E-29	Glomerular parietal epithelial cells	NUTF2
6.79E-33	0.3699693	0.676	0.438	1.12E-28	Glomerular parietal epithelial cells	TRAPPC1
8.15E-33	0.26848312	0.941	0.797	1.34E-28	Glomerular parietal epithelial cells	MIF
1.96E-32	0.29444413	0.322	0.141	3.22E-28	Glomerular parietal epithelial cells	SNX6
2.12E-32	0.36744053	0.368	0.175	3.49E-28	Glomerular parietal epithelial cells	DDAH2
2.40E-32	0.36852234	0.622	0.375	3.96E-28	Glomerular parietal epithelial cells	SNRPE
2.42E-32	0.30136164	0.316	0.139	3.98E-28	Glomerular parietal epithelial cells	TRIP6
3.42E-32	0.38432493	0.532	0.297	5.63E-28	Glomerular parietal epithelial cells	NHP2
4.20E-32	0.28553495	0.327	0.145	6.92E-28	Glomerular parietal epithelial cells	COMTD1
4.37E-32	0.25169999	0.984	0.936	7.19E-28	Glomerular parietal epithelial cells	RPL36
1.06E-31	0.32446621	0.395	0.192	1.74E-27	Glomerular parietal epithelial cells	EEF1E1
2.04E-31	0.33670526	0.286	0.123	3.35E-27	Glomerular parietal epithelial cells	TGIF1
2.66E-31	0.33027384	0.348	0.163	4.39E-27	Glomerular parietal epithelial cells	XRCC5
5.82E-31	0.2972033	0.335	0.153	9.59E-27	Glomerular parietal epithelial cells	2-Sep
2.21E-30	0.32541053	0.295	0.13	3.64E-26	Glomerular parietal epithelial cells	GNB2
2.56E-30	0.31113237	0.441	0.227	4.22E-26	Glomerular parietal epithelial cells	RANBP1
2.82E-30	0.29232613	0.9	0.747	4.64E-26	Glomerular parietal epithelial cells	SKP1
5.06E-30	0.31765015	0.576	0.335	8.33E-26	Glomerular parietal epithelial cells	PSMA4
5.70E-30	0.34290504	0.405	0.205	9.39E-26	Glomerular parietal epithelial cells	SSB
6.08E-30	0.31105669	0.3	0.134	1.00E-25	Glomerular parietal epithelial cells	CITED2
6.60E-30	0.32174687	0.833	0.607	1.09E-25	Glomerular parietal epithelial cells	SUB1
7.39E-30	0.35028808	0.363	0.178	1.22E-25	Glomerular parietal epithelial cells	ANXA11
7.43E-30	0.35393765	0.306	0.14	1.22E-25	Glomerular parietal epithelial cells	TUBA1C
1.20E-29	0.30051843	0.359	0.171	1.98E-25	Glomerular parietal epithelial cells	SNRPB
1.25E-29	0.26464471	0.965	0.877	2.05E-25	Glomerular parietal epithelial cells	RPS5
3.97E-29	0.35631194	0.376	0.189	6.54E-25	Glomerular parietal epithelial cells	SPX
4.35E-29	0.29383608	0.257	0.109	7.17E-25	Glomerular parietal epithelial cells	PSMD9
5.15E-29	0.37452256	0.751	0.521	8.48E-25	Glomerular parietal epithelial cells	RARRES3
5.84E-29	0.35215549	0.529	0.309	9.62E-25	Glomerular parietal epithelial cells	SERBP1
1.77E-28	0.32386729	0.595	0.359	2.92E-24	Glomerular parietal epithelial cells	VPS29
3.24E-28	0.28314374	0.306	0.141	5.34E-24	Glomerular parietal epithelial cells	UBE2A
5.97E-28	0.33315609	0.313	0.147	9.84E-24	Glomerular parietal epithelial cells	BICC1
6.14E-28	0.31925095	0.489	0.27	1.01E-23	Glomerular parietal epithelial cells	NCL
6.49E-28	0.33604071	0.776	0.548	1.07E-23	Glomerular parietal epithelial cells	MORF4L1
6.71E-28	0.34421241	0.552	0.327	1.10E-23	Glomerular parietal epithelial cells	ZFAS1
7.04E-28	0.35176445	0.46	0.254	1.16E-23	Glomerular parietal epithelial cells	C6orf48
1.26E-27	0.32800693	0.783	0.567	2.08E-23	Glomerular parietal epithelial cells	HMGB1
1.42E-27	0.27734288	0.268	0.117	2.34E-23	Glomerular parietal epithelial cells	CCT5
1.62E-27	0.26907742	0.324	0.153	2.66E-23	Glomerular parietal epithelial cells	MCRIP1
2.12E-27	0.32172562	0.51	0.285	3.50E-23	Glomerular parietal epithelial cells	CALD1
2.83E-27	0.3421858	0.716	0.49	4.66E-23	Glomerular parietal epithelial cells	HSP90AB1

3.41E-27	0.27973959	0.335	0.161	5.62E-23	Glomerular parietal epithelial cells	PAIP1
3.44E-27	0.31813549	0.492	0.28	5.66E-23	Glomerular parietal epithelial cells	AP2M1
5.11E-27	0.32419013	0.565	0.338	8.42E-23	Glomerular parietal epithelial cells	C11orf58
5.20E-27	0.31114023	0.851	0.696	8.56E-23	Glomerular parietal epithelial cells	BTF3
5.62E-27	0.30883232	0.711	0.487	9.25E-23	Glomerular parietal epithelial cells	CLTA
9.07E-27	0.32426793	0.53	0.313	1.49E-22	Glomerular parietal epithelial cells	SET
9.76E-27	0.26694912	0.91	0.761	1.61E-22	Glomerular parietal epithelial cells	RPL4
1.31E-26	0.26597086	0.883	0.699	2.17E-22	Glomerular parietal epithelial cells	DSTN
1.70E-26	0.31182307	0.421	0.226	2.80E-22	Glomerular parietal epithelial cells	SNRPD1
2.23E-26	0.26985347	0.33	0.159	3.68E-22	Glomerular parietal epithelial cells	MAGED2
3.59E-26	0.26170261	0.341	0.166	5.92E-22	Glomerular parietal epithelial cells	HNRNPU
3.76E-26	0.30899353	0.703	0.474	6.19E-22	Glomerular parietal epithelial cells	RPL17
7.99E-26	0.30939991	0.37	0.19	1.32E-21	Glomerular parietal epithelial cells	EIF4A1
1.08E-25	0.30137755	0.414	0.223	1.77E-21	Glomerular parietal epithelial cells	EIF3I
1.15E-25	0.28515962	0.279	0.129	1.89E-21	Glomerular parietal epithelial cells	HNRNPM
1.24E-25	0.26474324	0.376	0.191	2.04E-21	Glomerular parietal epithelial cells	YWHAZ
1.32E-25	0.27201809	0.286	0.131	2.17E-21	Glomerular parietal epithelial cells	EIF5A
2.07E-25	0.32092817	0.779	0.563	3.41E-21	Glomerular parietal epithelial cells	HMGN3
2.37E-25	0.27737396	0.379	0.195	3.91E-21	Glomerular parietal epithelial cells	ZBTB20
3.25E-25	0.31818412	0.763	0.554	5.35E-21	Glomerular parietal epithelial cells	EIF3E
6.18E-25	0.26672581	0.441	0.24	1.02E-20	Glomerular parietal epithelial cells	EIF3J
6.87E-25	0.29634323	0.632	0.401	1.13E-20	Glomerular parietal epithelial cells	ARL2
1.10E-24	0.27860862	0.463	0.259	1.81E-20	Glomerular parietal epithelial cells	BANF1
2.26E-24	0.35295719	0.711	0.502	3.72E-20	Glomerular parietal epithelial cells	DUSP23
2.29E-24	0.29244632	0.805	0.6	3.77E-20	Glomerular parietal epithelial cells	RPS17
3.30E-24	0.29052213	0.329	0.166	5.44E-20	Glomerular parietal epithelial cells	POLR2G
3.55E-24	0.27281004	0.283	0.133	5.84E-20	Glomerular parietal epithelial cells	IK
3.85E-24	0.26935493	0.376	0.196	6.35E-20	Glomerular parietal epithelial cells	BEX4
4.52E-24	0.28417417	0.454	0.254	7.45E-20	Glomerular parietal epithelial cells	MZT2A
5.40E-24	0.28852178	0.478	0.274	8.89E-20	Glomerular parietal epithelial cells	PSMA2
5.52E-24	0.27744898	0.405	0.217	9.09E-20	Glomerular parietal epithelial cells	COPZ1
8.74E-24	0.28415885	0.398	0.215	1.44E-19	Glomerular parietal epithelial cells	CCT3
8.74E-24	0.25200871	0.278	0.132	1.44E-19	Glomerular parietal epithelial cells	PNRC2
1.17E-23	0.34464307	0.429	0.243	1.93E-19	Glomerular parietal epithelial cells	PLIN2
1.63E-23	0.27517886	0.705	0.479	2.69E-19	Glomerular parietal epithelial cells	CYCS
1.81E-23	0.28220803	0.597	0.374	2.99E-19	Glomerular parietal epithelial cells	TNFSF10
1.89E-23	0.26919647	0.386	0.207	3.11E-19	Glomerular parietal epithelial cells	TBCB
2.07E-23	0.27933592	0.281	0.134	3.41E-19	Glomerular parietal epithelial cells	PUF60
2.50E-23	0.28256956	0.554	0.34	4.11E-19	Glomerular parietal epithelial cells	RHOA
3.26E-23	0.25243815	0.278	0.132	5.37E-19	Glomerular parietal epithelial cells	TXNDC9
3.81E-23	0.28461247	0.775	0.56	6.27E-19	Glomerular parietal epithelial cells	SNRPD2
5.56E-23	0.25025272	0.846	0.668	9.15E-19	Glomerular parietal epithelial cells	RPS26
7.77E-23	0.31192186	0.481	0.284	1.28E-18	Glomerular parietal epithelial cells	LACTB2
1.04E-22	0.25647797	0.483	0.279	1.72E-18	Glomerular parietal epithelial cells	HIGD1A
1.10E-22	0.31107821	0.665	0.467	1.80E-18	Glomerular parietal epithelial cells	CCNI
1.36E-22	0.27158544	0.529	0.314	2.25E-18	Glomerular parietal epithelial cells	EIF4G2
1.92E-22	0.2626792	0.484	0.28	3.16E-18	Glomerular parietal epithelial cells	NAP1L1
1.97E-22	0.26500926	0.456	0.264	3.24E-18	Glomerular parietal epithelial cells	TCEAL8
2.00E-22	0.27305361	0.292	0.145	3.30E-18	Glomerular parietal epithelial cells	KHDRBS1
2.07E-22	0.28596222	0.629	0.409	3.41E-18	Glomerular parietal epithelial cells	SPATS2L
3.50E-22	0.27004564	0.537	0.328	5.77E-18	Glomerular parietal epithelial cells	PTGES3
6.85E-22	0.25728942	0.324	0.167	1.13E-17	Glomerular parietal epithelial cells	MCTS1
8.63E-22	0.27616707	0.263	0.127	1.42E-17	Glomerular parietal epithelial cells	ACTR10
9.55E-22	0.28265207	0.343	0.182	1.57E-17	Glomerular parietal epithelial cells	ARHGDIA
1.37E-21	0.26158245	0.648	0.43	2.26E-17	Glomerular parietal epithelial cells	ERH
1.50E-21	0.25847747	0.516	0.306	2.47E-17	Glomerular parietal epithelial cells	HNRNPDL
2.05E-21	0.25568217	0.384	0.213	3.38E-17	Glomerular parietal epithelial cells	TPM3
2.13E-21	0.2591121	0.443	0.255	3.51E-17	Glomerular parietal epithelial cells	DUT

2.24E-21	0.26922054	0.514	0.311	3.69E-17	Glomerular parietal epithelial cells	LYPLAL1
2.33E-21	0.25406372	0.597	0.375	3.84E-17	Glomerular parietal epithelial cells	SMS
2.46E-21	0.30242814	0.571	0.37	4.05E-17	Glomerular parietal epithelial cells	SF3B6
2.75E-21	0.28627903	0.541	0.343	4.52E-17	Glomerular parietal epithelial cells	YWHAB
3.52E-21	0.27665801	0.794	0.615	5.79E-17	Glomerular parietal epithelial cells	SLC25A3
4.71E-21	0.29869448	0.616	0.41	7.75E-17	Glomerular parietal epithelial cells	AKR1C3
1.42E-20	0.28565345	0.411	0.236	2.33E-16	Glomerular parietal epithelial cells	RBPMS
1.47E-20	0.29990804	0.287	0.147	2.42E-16	Glomerular parietal epithelial cells	SH3GLB1
1.65E-20	0.25760877	0.608	0.397	2.72E-16	Glomerular parietal epithelial cells	SNU13
2.52E-20	0.26313555	0.763	0.562	4.15E-16	Glomerular parietal epithelial cells	ARPC3
4.01E-20	0.26690253	0.551	0.347	6.61E-16	Glomerular parietal epithelial cells	HNRNPC
4.79E-20	0.27329833	0.276	0.14	7.90E-16	Glomerular parietal epithelial cells	RCAN1
5.20E-20	0.27410079	0.59	0.383	8.57E-16	Glomerular parietal epithelial cells	SSBP1
1.15E-19	0.25098586	0.437	0.257	1.90E-15	Glomerular parietal epithelial cells	NARS
1.72E-19	0.25572615	0.7	0.494	2.83E-15	Glomerular parietal epithelial cells	PSMB3
4.06E-19	0.26242039	0.719	0.504	6.68E-15	Glomerular parietal epithelial cells	MRPS21
1.02E-18	0.27333154	0.519	0.325	1.68E-14	Glomerular parietal epithelial cells	RSL24D1
1.45E-18	0.25136158	0.708	0.502	2.38E-14	Glomerular parietal epithelial cells	YWHAE
1.82E-18	0.28042062	0.454	0.28	2.99E-14	Glomerular parietal epithelial cells	SRSF3
2.50E-18	0.30134076	0.635	0.44	4.11E-14	Glomerular parietal epithelial cells	CDC42
5.32E-18	0.26181448	0.665	0.471	8.76E-14	Glomerular parietal epithelial cells	BEX3
5.38E-18	0.26198688	0.348	0.196	8.87E-14	Glomerular parietal epithelial cells	LEPROT
5.61E-18	0.26254525	0.7	0.501	9.23E-14	Glomerular parietal epithelial cells	SEC61G
2.22E-17	0.27546597	0.443	0.275	3.65E-13	Glomerular parietal epithelial cells	UGP2
9.57E-17	0.25907922	0.687	0.502	1.58E-12	Glomerular parietal epithelial cells	NOP10
2.83E-16	0.25504725	0.362	0.214	4.66E-12	Glomerular parietal epithelial cells	PDHA1
6.55E-15	0.34611291	0.332	0.198	1.08E-10	Glomerular parietal epithelial cells	NUPR1
2.61E-14	0.25467619	0.64	0.481	4.29E-10	Glomerular parietal epithelial cells	RPS4Y1
0	3.88905318	0.896	0.129	0	Distal tubule cells	DEFB1
0	2.72404192	0.949	0.171	0	Distal tubule cells	S100A6
0	2.57337558	0.446	0.01	0	Distal tubule cells	S100A2
0	2.28936932	0.714	0.116	0	Distal tubule cells	CKB
0	2.15372927	0.324	0.002	0	Distal tubule cells	UMOD
0	2.01650145	0.317	0.002	0	Distal tubule cells	TMEM52B
0	1.89698966	0.475	0.003	0	Distal tubule cells	DUSP9
0	1.89319044	0.38	0.003	0	Distal tubule cells	PVALB
0	1.85007703	0.523	0.047	0	Distal tubule cells	GSTM3
0	1.80114082	0.346	0.003	0	Distal tubule cells	KNG1
0	1.51768067	0.397	0.017	0	Distal tubule cells	FOLR3
0	1.47712158	0.303	0.002	0	Distal tubule cells	TUBB2B
0	1.25470927	0.252	0.001	0	Distal tubule cells	LINC02121
3.19E-293	1.20200283	0.291	0.019	5.25E-289	Distal tubule cells	UCHL1
1.19E-218	1.41780842	0.39	0.048	1.95E-214	Distal tubule cells	NDRG2
9.48E-213	1.94720664	0.615	0.134	1.56E-208	Distal tubule cells	PPP1R1A
2.50E-139	1.20432817	0.366	0.064	4.13E-135	Distal tubule cells	NUDT4
3.80E-105	1.4508963	0.533	0.181	6.25E-101	Distal tubule cells	SLC25A4
2.34E-100	1.33376503	0.525	0.178	3.85E-96	Distal tubule cells	MRPS6
1.58E-99	0.99962971	0.908	0.668	2.60E-95	Distal tubule cells	CYSTM1
9.44E-98	1.42554687	0.557	0.208	1.55E-93	Distal tubule cells	IDH2
2.04E-95	1.13172558	0.782	0.471	3.35E-91	Distal tubule cells	BEX3
3.22E-94	0.92406452	0.908	0.695	5.31E-90	Distal tubule cells	CHCHD10
2.63E-92	0.78064172	0.959	0.843	4.33E-88	Distal tubule cells	COX6B1
9.74E-88	0.81397321	0.93	0.73	1.60E-83	Distal tubule cells	PPDPF
3.47E-87	1.23345157	0.709	0.379	5.72E-83	Distal tubule cells	MPC1
1.30E-83	1.13271559	0.729	0.4	2.14E-79	Distal tubule cells	S100A10
2.18E-83	1.11256449	0.763	0.48	3.58E-79	Distal tubule cells	CYCS
2.65E-82	1.20766071	0.467	0.154	4.37E-78	Distal tubule cells	COX7A1
2.96E-80	0.77117932	0.942	0.878	4.88E-76	Distal tubule cells	COX5B

3.44E-80	0.95549675	0.852	0.636	5.66E-76	Distal tubule cells	CALM2
1.23E-79	1.12024838	0.712	0.391	2.03E-75	Distal tubule cells	CD24
1.10E-75	1.06828194	0.37	0.106	1.81E-71	Distal tubule cells	ITGB1BP1
8.41E-73	0.78705863	0.901	0.759	1.38E-68	Distal tubule cells	COX8A
6.91E-69	0.74406339	0.889	0.737	1.14E-64	Distal tubule cells	UQCRH
3.13E-68	0.57854691	0.971	0.897	5.15E-64	Distal tubule cells	PTMA
1.23E-65	0.84427017	0.806	0.578	2.03E-61	Distal tubule cells	ATP5MC1
2.78E-65	1.14803426	0.521	0.231	4.58E-61	Distal tubule cells	PKM
1.01E-64	1.11840311	0.356	0.111	1.67E-60	Distal tubule cells	NFE2L2
8.85E-63	0.57768901	0.973	0.923	1.46E-58	Distal tubule cells	EIF1
3.50E-59	0.56246609	0.939	0.878	5.76E-55	Distal tubule cells	ATP5MG
5.65E-58	0.5775523	0.973	0.941	9.31E-54	Distal tubule cells	RPS4X
1.23E-56	0.64231794	0.92	0.808	2.03E-52	Distal tubule cells	ATP5MC3
1.94E-56	0.98865356	0.363	0.126	3.19E-52	Distal tubule cells	EFHD1
3.71E-56	0.66273466	0.913	0.814	6.11E-52	Distal tubule cells	COX7A2
6.89E-56	0.59481625	0.956	0.904	1.13E-51	Distal tubule cells	NDUFA4
2.87E-55	0.44043959	0.99	0.972	4.73E-51	Distal tubule cells	RPL3
1.22E-54	0.6622379	0.886	0.785	2.01E-50	Distal tubule cells	ATP5PF
1.69E-53	0.54814439	0.937	0.853	2.79E-49	Distal tubule cells	SRP14
2.02E-52	0.97640089	0.3	0.092	3.34E-48	Distal tubule cells	COMT
1.15E-49	0.75577866	0.317	0.1	1.90E-45	Distal tubule cells	IFITM2
2.56E-47	0.68992643	0.809	0.678	4.21E-43	Distal tubule cells	ATP5PO
5.38E-47	0.63934171	0.889	0.772	8.86E-43	Distal tubule cells	COX7B
8.28E-47	0.82259771	0.603	0.346	1.36E-42	Distal tubule cells	MRPL33
4.89E-46	0.64675137	0.872	0.774	8.05E-42	Distal tubule cells	NDUFA1
7.66E-46	0.72620338	0.758	0.58	1.26E-41	Distal tubule cells	COX5A
6.37E-45	0.8952631	0.479	0.241	1.05E-40	Distal tubule cells	NDUFA8
5.08E-43	0.62640281	0.889	0.783	8.36E-39	Distal tubule cells	UQCR10
2.62E-41	0.8477685	0.479	0.245	4.32E-37	Distal tubule cells	DMAC1
3.40E-41	0.59668951	0.864	0.779	5.60E-37	Distal tubule cells	COX6C
5.30E-41	0.74250259	0.683	0.492	8.74E-37	Distal tubule cells	NDUFA5
1.46E-40	0.85620711	0.443	0.217	2.40E-36	Distal tubule cells	DNAJC19
6.94E-39	0.75887516	0.654	0.458	1.14E-34	Distal tubule cells	NDUFA6
7.43E-39	0.97234282	0.409	0.194	1.22E-34	Distal tubule cells	BEX2
2.98E-38	0.37628363	0.983	0.952	4.92E-34	Distal tubule cells	RPL8
6.74E-38	0.5927026	0.801	0.666	1.11E-33	Distal tubule cells	YBX1
9.22E-37	0.88820093	0.38	0.175	1.52E-32	Distal tubule cells	GNAS
9.29E-37	0.43386376	0.923	0.887	1.53E-32	Distal tubule cells	RPL10A
2.32E-36	0.36781988	0.966	0.941	3.82E-32	Distal tubule cells	RPL15
4.39E-36	0.79088862	0.257	0.089	7.22E-32	Distal tubule cells	NME4
7.87E-36	0.9661007	0.492	0.292	1.30E-31	Distal tubule cells	CBR1
3.45E-35	0.75356226	0.576	0.383	5.68E-31	Distal tubule cells	CYC1
1.21E-34	0.55100291	0.84	0.701	2.00E-30	Distal tubule cells	DSTN
2.19E-34	0.53659167	0.823	0.754	3.60E-30	Distal tubule cells	CHCHD2
1.90E-33	0.57409941	0.857	0.748	3.12E-29	Distal tubule cells	TMA7
2.19E-33	0.6482572	0.692	0.543	3.60E-29	Distal tubule cells	NDUFB3
1.17E-32	0.50822905	0.879	0.816	1.93E-28	Distal tubule cells	UQCRB
1.37E-32	0.45143305	0.915	0.874	2.25E-28	Distal tubule cells	LDHB
1.50E-32	0.32211998	0.99	0.969	2.47E-28	Distal tubule cells	RPL7
3.49E-32	0.59998603	0.751	0.624	5.74E-28	Distal tubule cells	PRDX2
4.37E-32	0.71044878	0.649	0.484	7.20E-28	Distal tubule cells	MRPL41
6.94E-32	0.59736978	0.765	0.64	1.14E-27	Distal tubule cells	PRR13
1.22E-31	0.52026935	0.797	0.718	2.02E-27	Distal tubule cells	SLC25A6
1.75E-31	0.66183419	0.792	0.719	2.88E-27	Distal tubule cells	SLC25A5
3.39E-31	0.35612506	0.964	0.931	5.58E-27	Distal tubule cells	RPL6
4.40E-31	0.76729484	0.479	0.281	7.25E-27	Distal tubule cells	HIGD1A
4.53E-31	0.75218008	0.404	0.208	7.46E-27	Distal tubule cells	SRI
1.69E-30	0.86941889	0.453	0.262	2.79E-26	Distal tubule cells	MOCS2

2.69E-30	0.79876952	0.487	0.303	4.42E-26	Distal tubule cells	VDAC2
4.09E-30	0.51953763	0.748	0.609	6.73E-26	Distal tubule cells	H3F3B
8.09E-29	0.34099567	0.988	0.97	1.33E-24	Distal tubule cells	COX7C
1.16E-28	0.7738209	0.639	0.438	1.91E-24	Distal tubule cells	ADIRF
1.21E-28	0.33251181	0.942	0.911	2.00E-24	Distal tubule cells	COX4I1
1.40E-27	0.48441182	0.816	0.73	2.30E-23	Distal tubule cells	PRDX5
1.75E-27	0.54642692	0.729	0.611	2.88E-23	Distal tubule cells	SUB1
4.66E-27	0.40778859	0.843	0.786	7.68E-23	Distal tubule cells	ATP5F1D
8.24E-27	0.52877319	0.702	0.576	1.36E-22	Distal tubule cells	MPC2
1.09E-26	0.68465271	0.404	0.215	1.80E-22	Distal tubule cells	TXNIP
3.88E-26	0.64407578	0.634	0.517	6.40E-22	Distal tubule cells	UQCRFS1
6.52E-26	0.7128315	0.489	0.319	1.07E-21	Distal tubule cells	NDUFA12
6.83E-26	0.62729072	0.688	0.575	1.13E-21	Distal tubule cells	NDUFS6
9.39E-26	0.74364612	0.441	0.267	1.55E-21	Distal tubule cells	GLRX5
2.06E-25	0.7986466	0.433	0.259	3.39E-21	Distal tubule cells	ID2
1.11E-24	0.53416322	0.835	0.74	1.82E-20	Distal tubule cells	UQCR11
1.77E-24	0.50404386	0.826	0.749	2.91E-20	Distal tubule cells	SKP1
7.97E-24	0.74033293	0.383	0.213	1.31E-19	Distal tubule cells	SVIP
3.57E-23	0.59474116	0.591	0.448	5.87E-19	Distal tubule cells	C19orf70
3.90E-23	0.47443811	0.751	0.664	6.43E-19	Distal tubule cells	NDUFB4
4.62E-23	0.26974218	0.935	0.867	7.61E-19	Distal tubule cells	RPS2
5.71E-23	0.66302303	0.61	0.498	9.41E-19	Distal tubule cells	CIRBP
5.81E-23	0.60050492	0.535	0.379	9.58E-19	Distal tubule cells	SMS
1.15E-22	0.64275527	0.448	0.284	1.89E-18	Distal tubule cells	SNRPN
1.29E-22	0.58611904	0.581	0.433	2.13E-18	Distal tubule cells	ERH
3.17E-22	0.68288013	0.363	0.2	5.22E-18	Distal tubule cells	PFN2
4.14E-22	0.51363286	0.717	0.637	6.81E-18	Distal tubule cells	NDUFB7
1.31E-21	0.66044445	0.458	0.298	2.16E-17	Distal tubule cells	PNKD
3.84E-21	0.52647917	0.617	0.481	6.32E-17	Distal tubule cells	HMGN1
4.70E-21	0.3423198	0.947	0.898	7.74E-17	Distal tubule cells	PEBP1
8.17E-21	0.47468237	0.68	0.589	1.35E-16	Distal tubule cells	SUMO2
1.19E-20	0.54681631	0.579	0.422	1.97E-16	Distal tubule cells	ACTG1
1.87E-19	0.51753243	0.678	0.582	3.08E-15	Distal tubule cells	ATP5F1A
5.78E-19	0.38966391	0.843	0.77	9.53E-15	Distal tubule cells	UBL5
6.56E-19	0.52932746	0.576	0.47	1.08E-14	Distal tubule cells	CCNI
1.52E-18	0.52604585	0.649	0.574	2.50E-14	Distal tubule cells	NDUFB10
1.64E-18	0.56356569	0.569	0.444	2.70E-14	Distal tubule cells	GABARAPL2
2.48E-18	0.62665464	0.487	0.355	4.08E-14	Distal tubule cells	NDUFS4
2.72E-18	0.64235815	0.366	0.225	4.48E-14	Distal tubule cells	VAMP2
3.10E-18	0.43758631	0.734	0.675	5.11E-14	Distal tubule cells	ISCU
3.57E-18	0.71513406	0.288	0.153	5.88E-14	Distal tubule cells	SELENOM
5.95E-18	0.67808886	0.363	0.222	9.80E-14	Distal tubule cells	FAM200B
8.81E-18	0.6647251	0.3	0.166	1.45E-13	Distal tubule cells	C7orf50
2.31E-17	0.60238451	0.501	0.374	3.81E-13	Distal tubule cells	TMEM141
2.34E-17	0.32839494	0.884	0.835	3.86E-13	Distal tubule cells	ATP5MC2
6.83E-17	0.62558282	0.523	0.4	1.13E-12	Distal tubule cells	SNU13
8.78E-17	0.36437385	0.809	0.765	1.45E-12	Distal tubule cells	RPL4
9.05E-17	0.55957524	0.562	0.447	1.49E-12	Distal tubule cells	ANAPC16
9.55E-17	0.26100938	0.915	0.874	1.57E-12	Distal tubule cells	MYL6
1.76E-16	0.46457024	0.634	0.548	2.89E-12	Distal tubule cells	NDUFB9
2.10E-16	0.60910522	0.349	0.216	3.47E-12	Distal tubule cells	PDHA1
3.31E-16	0.48945454	0.654	0.578	5.44E-12	Distal tubule cells	PRDX3
3.92E-16	0.58984392	0.257	0.133	6.46E-12	Distal tubule cells	MRPL16
8.44E-16	0.45136245	0.685	0.608	1.39E-11	Distal tubule cells	COMMD6
9.21E-16	0.5327046	0.535	0.421	1.52E-11	Distal tubule cells	COX7A2L
1.17E-15	0.4552314	0.622	0.53	1.93E-11	Distal tubule cells	NDUFS7
1.49E-15	0.41642106	0.702	0.624	2.46E-11	Distal tubule cells	EIF3K
1.73E-15	0.38839483	0.683	0.598	2.85E-11	Distal tubule cells	ENO1

3.53E-15	0.64492362	0.373	0.247	5.81E-11	Distal tubule cells	C12orf57
4.40E-15	0.3373918	0.799	0.753	7.25E-11	Distal tubule cells	TPI1
5.95E-15	0.5070298	0.654	0.583	9.81E-11	Distal tubule cells	ATP5F1B
6.72E-15	0.35203201	0.828	0.8	1.11E-10	Distal tubule cells	MIF
6.88E-15	0.50443631	0.574	0.479	1.13E-10	Distal tubule cells	TSTD1
7.66E-15	0.41957871	0.646	0.55	1.26E-10	Distal tubule cells	PRDX6
9.74E-15	0.49804154	0.547	0.449	1.60E-10	Distal tubule cells	NDUFAB1
1.28E-14	0.69695437	0.317	0.2	2.12E-10	Distal tubule cells	MRPL36
2.26E-14	0.53940295	0.453	0.339	3.72E-10	Distal tubule cells	TMEM230
3.47E-14	0.52991689	0.259	0.142	5.72E-10	Distal tubule cells	LITAF
3.49E-14	0.79948154	0.283	0.166	5.74E-10	Distal tubule cells	TSC22D1
3.50E-14	0.45915082	0.688	0.635	5.77E-10	Distal tubule cells	ATP5PD
7.67E-14	0.36135561	0.818	0.792	1.26E-09	Distal tubule cells	ATP5MPL
7.85E-14	0.25900014	0.971	0.956	1.29E-09	Distal tubule cells	RPS8
7.85E-14	0.55100914	0.412	0.289	1.29E-09	Distal tubule cells	HADHB
1.19E-13	0.30869046	0.828	0.777	1.97E-09	Distal tubule cells	COX6A1
1.61E-13	0.61476406	0.368	0.247	2.65E-09	Distal tubule cells	UQCC2
2.68E-13	0.42550637	0.584	0.494	4.42E-09	Distal tubule cells	HSP90AB1
2.95E-13	0.57230258	0.419	0.31	4.85E-09	Distal tubule cells	HIBADH
3.23E-13	0.54158169	0.378	0.26	5.32E-09	Distal tubule cells	NARS
3.96E-13	0.46247882	0.542	0.447	6.52E-09	Distal tubule cells	HSBP1
5.22E-13	0.54566063	0.317	0.2	8.60E-09	Distal tubule cells	CXXC5
6.44E-13	0.51731763	0.402	0.289	1.06E-08	Distal tubule cells	UQCRC2
6.87E-13	0.28407077	0.884	0.845	1.13E-08	Distal tubule cells	NDUFB2
1.88E-12	0.53613458	0.484	0.388	3.10E-08	Distal tubule cells	EID1
1.91E-12	0.60606681	0.291	0.181	3.14E-08	Distal tubule cells	ANXA11
2.09E-12	0.30186194	0.797	0.756	3.45E-08	Distal tubule cells	ATP5MF
2.45E-12	0.36093427	0.753	0.73	4.03E-08	Distal tubule cells	TOMM7
3.39E-12	0.44252558	0.579	0.513	5.58E-08	Distal tubule cells	GNG5
5.49E-12	0.59873039	0.38	0.271	9.05E-08	Distal tubule cells	TCEAL4
7.12E-12	0.33322624	0.697	0.653	1.17E-07	Distal tubule cells	MGST3
7.26E-12	0.6084915	0.429	0.332	1.20E-07	Distal tubule cells	SELENOW
1.39E-11	0.27817205	0.833	0.821	2.30E-07	Distal tubule cells	RPLP0
1.51E-11	0.54750944	0.31	0.205	2.48E-07	Distal tubule cells	LYPLA1
1.59E-11	0.47945943	0.462	0.363	2.62E-07	Distal tubule cells	SRSF9
1.81E-11	0.55264807	0.38	0.271	2.98E-07	Distal tubule cells	TCEAL9
2.12E-11	0.44999835	0.492	0.388	3.49E-07	Distal tubule cells	NAA38
2.34E-11	0.40299808	0.603	0.547	3.86E-07	Distal tubule cells	NDUFV2
3.22E-11	0.53676189	0.38	0.279	5.31E-07	Distal tubule cells	BAG1
3.51E-11	0.55458179	0.264	0.162	5.78E-07	Distal tubule cells	MAGED2
3.53E-11	0.49017035	0.433	0.332	5.81E-07	Distal tubule cells	ANAPC13
3.78E-11	0.49795642	0.308	0.201	6.23E-07	Distal tubule cells	SNX10
4.20E-11	0.49686326	0.293	0.188	6.92E-07	Distal tubule cells	ETFRF1
1.00E-10	0.40146932	0.525	0.437	1.65E-06	Distal tubule cells	SRP9
1.14E-10	0.35984435	0.639	0.581	1.87E-06	Distal tubule cells	ATP5F1C
1.22E-10	0.3207796	0.69	0.632	2.01E-06	Distal tubule cells	ATP6V1G1
1.24E-10	0.2923506	0.801	0.762	2.05E-06	Distal tubule cells	PPIA
1.48E-10	0.31007541	0.755	0.729	2.44E-06	Distal tubule cells	OST4
1.58E-10	0.41806904	0.521	0.446	2.60E-06	Distal tubule cells	VDAC1
1.82E-10	0.29036075	0.729	0.695	3.00E-06	Distal tubule cells	RPL36AL
2.01E-10	0.51160865	0.351	0.252	3.31E-06	Distal tubule cells	HDDC2
3.29E-10	0.34811648	0.484	0.349	5.41E-06	Distal tubule cells	PCP4
3.45E-10	0.35763678	0.63	0.583	5.68E-06	Distal tubule cells	NDUFB8
4.91E-10	0.39879176	0.559	0.483	8.09E-06	Distal tubule cells	CISD1
5.73E-10	0.48266204	0.3	0.199	9.43E-06	Distal tubule cells	BEX4
7.77E-10	0.52452292	0.332	0.235	1.28E-05	Distal tubule cells	SH3BGRL
9.91E-10	0.52421188	0.32	0.223	1.63E-05	Distal tubule cells	MRPS12
1.01E-09	0.49484484	0.305	0.206	1.66E-05	Distal tubule cells	AIF1L

1.08E-09	0.41481657	0.496	0.418	1.77E-05	Distal tubule cells	NDUFB6
1.23E-09	0.43451896	0.385	0.283	2.02E-05	Distal tubule cells	SRSF3
1.34E-09	0.47847566	0.317	0.218	2.21E-05	Distal tubule cells	TSPO
1.43E-09	0.33777107	0.673	0.63	2.35E-05	Distal tubule cells	EEF2
2.16E-09	0.35129337	0.678	0.641	3.56E-05	Distal tubule cells	DYNLL1
2.60E-09	0.54827864	0.286	0.195	4.28E-05	Distal tubule cells	VDAC3
2.95E-09	0.40986194	0.605	0.559	4.86E-05	Distal tubule cells	MINOS1
4.27E-09	0.2952109	0.639	0.571	7.03E-05	Distal tubule cells	HMGB1
4.46E-09	0.34092047	0.642	0.608	7.35E-05	Distal tubule cells	PARK7
4.60E-09	0.42658491	0.496	0.435	7.58E-05	Distal tubule cells	HINT2
4.67E-09	0.5194191	0.329	0.242	7.69E-05	Distal tubule cells	SPAG7
6.52E-09	0.49287604	0.375	0.292	0.0001074	Distal tubule cells	RHEB
7.91E-09	0.48740019	0.46	0.386	0.0001303	Distal tubule cells	FAM162A
8.76E-09	0.49971481	0.419	0.343	0.0001442	Distal tubule cells	C11orf58
1.07E-08	0.35192867	0.63	0.588	0.0001763	Distal tubule cells	NDUFS8
1.10E-08	0.47089527	0.305	0.218	0.0001809	Distal tubule cells	PDCD6
1.46E-08	0.44404144	0.354	0.263	0.0002406	Distal tubule cells	BANF1
1.59E-08	0.49407499	0.414	0.34	0.0002621	Distal tubule cells	MMP24OS
1.60E-08	0.48905629	0.305	0.22	0.0002637	Distal tubule cells	TXN2
2.01E-08	0.44667834	0.368	0.283	0.0003303	Distal tubule cells	NDUFS3
2.03E-08	0.36578808	0.581	0.539	0.0003339	Distal tubule cells	PHPT1
2.09E-08	0.36067389	0.564	0.506	0.0003447	Distal tubule cells	YWHAE
2.68E-08	0.42792302	0.254	0.166	0.0004418	Distal tubule cells	GOT1
2.86E-08	0.49414302	0.257	0.171	0.0004705	Distal tubule cells	OTUD6B-AS1
2.98E-08	0.27994363	0.738	0.698	0.0004904	Distal tubule cells	NDUFC1
3.10E-08	0.50829369	0.351	0.267	0.0005104	Distal tubule cells	TXNL4A
3.12E-08	0.43104123	0.317	0.232	0.000514	Distal tubule cells	RANBP1
4.88E-08	0.50584806	0.329	0.252	0.0008045	Distal tubule cells	NBDY
5.71E-08	0.50975041	0.322	0.246	0.0009401	Distal tubule cells	KRT10
7.73E-08	0.47532984	0.288	0.203	0.0012737	Distal tubule cells	EIF4E
1.08E-07	0.50997197	0.3	0.222	0.0017711	Distal tubule cells	ANXA5
1.13E-07	0.4380324	0.373	0.296	0.0018538	Distal tubule cells	PPCS
1.13E-07	0.45097998	0.402	0.332	0.0018604	Distal tubule cells	RAB2A
1.25E-07	0.41341602	0.424	0.351	0.0020596	Distal tubule cells	MRPL34
1.28E-07	0.45066904	0.356	0.28	0.0021056	Distal tubule cells	YWHAQ
1.29E-07	0.33348285	0.579	0.544	0.0021279	Distal tubule cells	AURKAIP1
1.54E-07	0.49083828	0.322	0.245	0.0025394	Distal tubule cells	BOLA3
2.33E-07	0.63186718	0.288	0.212	0.0038437	Distal tubule cells	CHP1
3.38E-07	0.46118535	0.329	0.259	0.0055695	Distal tubule cells	CNBP
4.34E-07	0.37622204	0.484	0.434	0.007156	Distal tubule cells	ANAPC11
5.65E-07	0.44585363	0.346	0.276	0.0093118	Distal tubule cells	AP1S1
5.68E-07	0.35476622	0.438	0.374	0.0093637	Distal tubule cells	SF3B6
7.69E-07	0.32204482	0.506	0.467	0.0126608	Distal tubule cells	LAMTOR4
8.23E-07	0.33226652	0.341	0.253	0.0135506	Distal tubule cells	MCCD1
8.73E-07	0.41063637	0.324	0.249	0.0143711	Distal tubule cells	TUBB
9.45E-07	0.40799513	0.293	0.218	0.0155653	Distal tubule cells	MRPS18C
9.80E-07	0.37551883	0.446	0.381	0.0161438	Distal tubule cells	SNRPE
1.02E-06	0.29026252	0.533	0.479	0.0167274	Distal tubule cells	SMDT1
1.06E-06	0.26798329	0.678	0.646	0.0175168	Distal tubule cells	NDUFA11
1.17E-06	0.39618421	0.409	0.35	0.0192312	Distal tubule cells	POLR2K
1.24E-06	0.4350864	0.407	0.347	0.0204782	Distal tubule cells	UFC1
1.55E-06	0.42494917	0.361	0.293	0.0254551	Distal tubule cells	TMEM14B
1.69E-06	0.38642395	0.414	0.346	0.0278413	Distal tubule cells	SMIM30
2.02E-06	0.37943973	0.448	0.392	0.0332291	Distal tubule cells	NDUFB5
2.12E-06	0.44017621	0.259	0.189	0.034994	Distal tubule cells	AK3
2.34E-06	0.39898877	0.291	0.221	0.0385778	Distal tubule cells	COPZ1
2.49E-06	0.39835943	0.426	0.376	0.0409618	Distal tubule cells	SDHD
2.52E-06	0.40133644	0.383	0.318	0.0414425	Distal tubule cells	SET

2.58E-06	0.35257928	0.298	0.221	0.0424181	Distal tubule cells	MRPS7
3.04E-06	0.42607541	0.366	0.308	0.0500422	Distal tubule cells	TAX1BP1
3.93E-06	0.2592331	0.472	0.394	0.0647461	Distal tubule cells	IFITM3
4.09E-06	0.37643023	0.462	0.428	0.0673422	Distal tubule cells	CAPZA2
4.09E-06	0.44774491	0.254	0.186	0.0673863	Distal tubule cells	UBE2E3
4.14E-06	0.30244486	0.581	0.554	0.0682462	Distal tubule cells	MORF4L1
4.39E-06	0.3836968	0.332	0.264	0.0722657	Distal tubule cells	MAP3K13
4.47E-06	0.40722245	0.327	0.254	0.0736237	Distal tubule cells	MRPL21
4.76E-06	0.27903449	0.724	0.711	0.0784398	Distal tubule cells	NDUFB1
4.95E-06	0.28754461	0.571	0.547	0.081522	Distal tubule cells	GUK1
5.19E-06	0.35515223	0.528	0.506	0.0854408	Distal tubule cells	SEC61G
5.71E-06	0.41128403	0.271	0.202	0.0941164	Distal tubule cells	PCNP
7.96E-06	0.39614934	0.303	0.236	0.131065	Distal tubule cells	MIEN1
8.16E-06	0.38942949	0.32	0.249	0.1343697	Distal tubule cells	TUBA1B
9.14E-06	0.31714669	0.446	0.395	0.1505161	Distal tubule cells	EIF3H
9.55E-06	0.33993287	0.472	0.44	0.1573409	Distal tubule cells	SUMO1
1.15E-05	0.33681041	0.511	0.471	0.1893576	Distal tubule cells	CLIC1
1.16E-05	0.32473394	0.55	0.526	0.1906265	Distal tubule cells	COX17
1.16E-05	0.38459464	0.363	0.302	0.1906582	Distal tubule cells	NHP2
1.49E-05	0.36848803	0.305	0.242	0.245359	Distal tubule cells	MRPL40
1.51E-05	0.38590096	0.286	0.219	0.2493904	Distal tubule cells	FKBP3
1.65E-05	0.26426829	0.632	0.625	0.2709597	Distal tubule cells	NDUFA2
1.83E-05	0.38137963	0.308	0.246	0.3017332	Distal tubule cells	PYURF
2.42E-05	0.41020405	0.332	0.276	0.3984609	Distal tubule cells	PGAM1
2.53E-05	0.26177486	0.806	0.812	0.4169676	Distal tubule cells	ATP5ME
4.16E-05	0.32825076	0.443	0.383	0.685986	Distal tubule cells	RWDD1
6.12E-05	0.40586398	0.327	0.274	1	Distal tubule cells	RAB11A
6.33E-05	0.37660501	0.361	0.317	1	Distal tubule cells	EIF3L
6.80E-05	0.44740446	0.334	0.284	1	Distal tubule cells	AP2M1
6.92E-05	0.2539121	0.574	0.565	1	Distal tubule cells	SNRPD2
7.94E-05	0.33965611	0.264	0.204	1	Distal tubule cells	MEAF6
8.80E-05	0.40624972	0.305	0.245	1	Distal tubule cells	MIR4458HG
0.000105	0.31163476	0.45	0.405	1	Distal tubule cells	CSTB
0.000107	0.26976512	0.501	0.476	1	Distal tubule cells	RBX1
0.000124	0.33007569	0.395	0.354	1	Distal tubule cells	MRPL27
0.00013	0.36885878	0.298	0.243	1	Distal tubule cells	MAP1LC3B
0.000142	0.34280481	0.424	0.392	1	Distal tubule cells	UBE2D3
0.000195	0.33891467	0.262	0.207	1	Distal tubule cells	MRPS25
0.000246	0.4252549	0.291	0.241	1	Distal tubule cells	DDX24
0.000265	0.29722343	0.368	0.321	1	Distal tubule cells	HIST1H4C
0.000299	0.34693267	0.351	0.304	1	Distal tubule cells	SERP1
0.000329	0.29674488	0.339	0.287	1	Distal tubule cells	SCOC
0.000355	0.2684821	0.271	0.212	1	Distal tubule cells	BTG1
0.000363	0.37575711	0.264	0.211	1	Distal tubule cells	DNAJC8
0.000418	0.39611288	0.259	0.211	1	Distal tubule cells	MRPL47
0.000429	0.37946203	0.518	0.49	1	Distal tubule cells	ATP1B1
0.000466	0.32822973	0.252	0.201	1	Distal tubule cells	POLDIP2
0.000543	0.33500001	0.283	0.235	1	Distal tubule cells	SDCBP
0.000551	0.32546742	0.303	0.256	1	Distal tubule cells	MRPL13
0.000561	0.35095692	0.324	0.283	1	Distal tubule cells	PFDN2
0.000578	0.43582369	0.346	0.317	1	Distal tubule cells	HMGN2
0.000584	0.28416502	0.373	0.328	1	Distal tubule cells	MRPS33
0.000629	0.3671431	0.257	0.207	1	Distal tubule cells	MRPL22
0.000634	0.31185092	0.305	0.259	1	Distal tubule cells	C6orf48
0.000647	0.37495992	0.257	0.212	1	Distal tubule cells	NUDC
0.000676	0.29065734	0.254	0.202	1	Distal tubule cells	FUNDC2
0.00076	0.40203103	0.269	0.221	1	Distal tubule cells	PPP1CB
0.000866	0.2676561	0.455	0.44	1	Distal tubule cells	MRPL51

0.001043	0.30386558	0.45	0.435	1	Distal tubule cells	MRPL20
0.001059	0.28608269	0.327	0.286	1	Distal tubule cells	SLC25A39
0.001062	0.26502661	0.402	0.371	1	Distal tubule cells	MRPL54
0.001114	0.26194505	0.424	0.401	1	Distal tubule cells	NDUFV1
0.001206	0.30054184	0.467	0.45	1	Distal tubule cells	HIGD2A
0.001246	0.25673658	0.521	0.521	1	Distal tubule cells	SNX3
0.001388	0.35526354	0.305	0.268	1	Distal tubule cells	TCEAL8
0.001496	0.28224523	0.351	0.31	1	Distal tubule cells	NDUFS2
0.001607	0.28176102	0.378	0.342	1	Distal tubule cells	NUCKS1
0.001783	0.26013263	0.414	0.388	1	Distal tubule cells	MDH2
0.001851	0.32904439	0.395	0.369	1	Distal tubule cells	COX20
0.002138	0.37934573	0.312	0.277	1	Distal tubule cells	ATP1A1
0.002199	0.30540464	0.295	0.257	1	Distal tubule cells	ECI1
0.002301	0.28180903	0.262	0.214	1	Distal tubule cells	G0S2
0.002376	0.32925116	0.329	0.296	1	Distal tubule cells	SNRPD3
0.002579	0.32923809	0.295	0.259	1	Distal tubule cells	C1orf122
0.002959	0.27361018	0.436	0.417	1	Distal tubule cells	COPE
0.002975	0.29642389	0.259	0.22	1	Distal tubule cells	RAB18
0.003379	0.35827242	0.291	0.257	1	Distal tubule cells	SRSF5
0.003511	0.31460532	0.322	0.295	1	Distal tubule cells	MLF2
0.003624	0.31415095	0.269	0.234	1	Distal tubule cells	STRAP
0.00365	0.30745222	0.334	0.308	1	Distal tubule cells	HEBP2
0.003997	0.29266908	0.358	0.333	1	Distal tubule cells	PTGES3
0.004332	0.29213721	0.276	0.238	1	Distal tubule cells	MRPS16
0.004373	0.36974227	0.278	0.246	1	Distal tubule cells	POLR2J
0.00444	0.29291661	0.303	0.271	1	Distal tubule cells	SMIM37
0.004831	0.29032921	0.385	0.361	1	Distal tubule cells	VAPA
0.005034	0.26392982	0.337	0.303	1	Distal tubule cells	STUB1
0.005374	0.32263606	0.312	0.286	1	Distal tubule cells	EIF1B
0.006017	0.32252187	0.281	0.243	1	Distal tubule cells	THOC7
0.007402	0.27893208	0.262	0.227	1	Distal tubule cells	MRPS35
0.00745	0.28472226	0.271	0.239	1	Distal tubule cells	PMVK
0.007612	0.3466576	0.303	0.277	1	Distal tubule cells	CAPZB
0.009643	0.25511875	0.392	0.38	1	Distal tubule cells	ATP5PB
0.009926	0.27463863	0.397	0.387	1	Distal tubule cells	HNRNPK
0	3.87697066	0.592	0.004	0	Collecting duct principal cells	FXYD4
0	3.69594487	0.579	0.003	0	Collecting duct principal cells	AQP2
0	3.31942253	0.559	0.003	0	Collecting duct principal cells	SLPI
0	2.42306319	0.645	0.003	0	Collecting duct principal cells	TACSTD2
0	2.31981043	0.651	0.026	0	Collecting duct principal cells	CD9
0	2.06607041	0.559	0.018	0	Collecting duct principal cells	KRT19
0	1.73735939	0.467	0.002	0	Collecting duct principal cells	MUC1
0	1.61002783	0.329	0.008	0	Collecting duct principal cells	ID1
0	1.53461698	0.467	0.006	0	Collecting duct principal cells	SCIN
0	1.4728089	0.467	0.01	0	Collecting duct principal cells	MAL
0	1.47127678	0.421	0.01	0	Collecting duct principal cells	KRT7
0	1.33937377	0.296	0.002	0	Collecting duct principal cells	TFPI2
0	1.24217243	0.342	0.002	0	Collecting duct principal cells	IGFBP2
0	1.22854042	0.257	0.002	0	Collecting duct principal cells	IGFBP3
0	1.21262641	0.355	0.003	0	Collecting duct principal cells	ST6GAL1
0	1.17418034	0.355	0	0	Collecting duct principal cells	CLDN8
0	1.11615291	0.362	0.006	0	Collecting duct principal cells	SLC38A1
0	1.0430704	0.329	0.005	0	Collecting duct principal cells	PFKFB3
0	1.03484237	0.303	0.007	0	Collecting duct principal cells	CD82
0	0.99155356	0.296	0.004	0	Collecting duct principal cells	GATA3
0	0.97578495	0.283	0.005	0	Collecting duct principal cells	CDH1
0	0.93942922	0.283	0.001	0	Collecting duct principal cells	KSR2
0	0.87902705	0.303	0.006	0	Collecting duct principal cells	RHOBTB3

6.75E-307	1.04287575	0.388	0.013	1.11E-302	Collecting duct principal cells	DMKN
2.46E-301	0.94857747	0.276	0.006	4.06E-297	Collecting duct principal cells	TCIM
7.23E-284	2.58652588	0.586	0.035	1.19E-279	Collecting duct principal cells	AQP3
1.31E-283	1.40478565	0.513	0.026	2.15E-279	Collecting duct principal cells	MAL2
3.08E-275	0.79833856	0.283	0.007	5.07E-271	Collecting duct principal cells	SCNN1A
1.31E-253	0.88372827	0.289	0.009	2.15E-249	Collecting duct principal cells	MPZL2
1.71E-191	1.08988791	0.336	0.016	2.82E-187	Collecting duct principal cells	GNG11
3.75E-191	1.06044648	0.375	0.02	6.17E-187	Collecting duct principal cells	AHNAK
2.18E-157	0.94778936	0.329	0.019	3.59E-153	Collecting duct principal cells	SLC25A29
8.86E-157	2.71447977	0.921	0.18	1.46E-152	Collecting duct principal cells	S100A6
1.40E-146	1.04753664	0.368	0.026	2.31E-142	Collecting duct principal cells	RDH10
2.24E-140	1.03534461	0.362	0.026	3.69E-136	Collecting duct principal cells	CLDN4
4.29E-140	1.14349448	0.493	0.048	7.07E-136	Collecting duct principal cells	TPM4
6.24E-140	1.08139274	0.362	0.026	1.03E-135	Collecting duct principal cells	CRIP2
2.63E-138	0.91473066	0.276	0.015	4.33E-134	Collecting duct principal cells	SLC5A3
1.09E-137	0.96442751	0.322	0.021	1.80E-133	Collecting duct principal cells	AGRN
2.01E-134	2.22677504	0.645	0.088	3.31E-130	Collecting duct principal cells	WFDC2
5.70E-132	1.03862684	0.303	0.019	9.39E-128	Collecting duct principal cells	HSD11B2
2.52E-131	0.95291257	0.263	0.014	4.14E-127	Collecting duct principal cells	RASD1
9.71E-131	1.31617193	0.796	0.124	1.60E-126	Collecting duct principal cells	NEAT1
5.81E-119	1.04683311	0.382	0.034	9.57E-115	Collecting duct principal cells	COBLL1
2.32E-116	0.8731679	0.349	0.028	3.82E-112	Collecting duct principal cells	C4orf48
1.30E-115	1.77672957	0.382	0.035	2.15E-111	Collecting duct principal cells	MMP7
9.88E-111	0.80555867	0.309	0.024	1.63E-106	Collecting duct principal cells	NFAT5
7.77E-105	1.50114592	0.553	0.082	1.28E-100	Collecting duct principal cells	ANXA2
5.03E-101	0.94973094	0.388	0.04	8.28E-97	Collecting duct principal cells	CD81
3.40E-95	0.79216865	0.296	0.025	5.61E-91	Collecting duct principal cells	ERBB2
1.99E-90	2.23068914	0.895	0.299	3.28E-86	Collecting duct principal cells	MALAT1
1.18E-86	1.62296983	0.487	0.075	1.94E-82	Collecting duct principal cells	AKR1B1
2.03E-84	1.45982625	0.632	0.128	3.35E-80	Collecting duct principal cells	CDH16
1.21E-76	0.8265168	0.329	0.038	1.99E-72	Collecting duct principal cells	PAX2
3.28E-74	1.03283386	0.408	0.059	5.40E-70	Collecting duct principal cells	WSB1
1.14E-72	1.18430029	0.684	0.163	1.87E-68	Collecting duct principal cells	APP
1.77E-72	2.37195291	0.796	0.28	2.91E-68	Collecting duct principal cells	CLU
6.46E-68	0.68478108	0.27	0.028	1.06E-63	Collecting duct principal cells	PLS3
2.91E-67	0.58706289	0.257	0.025	4.80E-63	Collecting duct principal cells	RGS2
1.02E-66	1.09283344	0.461	0.082	1.67E-62	Collecting duct principal cells	CLDN7
7.97E-65	1.20008623	0.572	0.126	1.31E-60	Collecting duct principal cells	KTN1
1.01E-63	0.95971116	0.599	0.139	1.66E-59	Collecting duct principal cells	DEFB1
6.72E-62	0.85793493	0.27	0.031	1.11E-57	Collecting duct principal cells	BTG2
1.19E-61	1.12682848	0.921	0.39	1.97E-57	Collecting duct principal cells	TMSB4X
1.51E-59	0.69311061	0.27	0.032	2.49E-55	Collecting duct principal cells	SPTAN1
3.99E-57	0.87693146	0.322	0.046	6.57E-53	Collecting duct principal cells	CRIM1
1.95E-55	0.87184324	0.349	0.056	3.21E-51	Collecting duct principal cells	SYNGR2
2.11E-54	0.66263919	0.263	0.033	3.47E-50	Collecting duct principal cells	ARID1B
1.02E-52	1.20370771	0.533	0.132	1.68E-48	Collecting duct principal cells	RALBP1
2.13E-52	1.33564384	0.684	0.233	3.51E-48	Collecting duct principal cells	PKM
3.69E-52	1.09227642	0.579	0.151	6.07E-48	Collecting duct principal cells	BCAM
6.17E-51	0.76542931	0.296	0.043	1.02E-46	Collecting duct principal cells	JUP
7.83E-50	0.86330954	0.329	0.054	1.29E-45	Collecting duct principal cells	GSTM3
1.83E-49	1.20925727	0.375	0.072	3.02E-45	Collecting duct principal cells	PDK4
4.52E-49	0.95162692	0.507	0.12	7.45E-45	Collecting duct principal cells	DDX17
1.58E-47	0.93179193	0.421	0.088	2.60E-43	Collecting duct principal cells	JUND
1.62E-47	0.67114064	0.329	0.055	2.67E-43	Collecting duct principal cells	SPTBN1
3.10E-46	0.59943599	0.263	0.038	5.11E-42	Collecting duct principal cells	SCARB2
1.42E-45	0.78354164	0.289	0.046	2.34E-41	Collecting duct principal cells	PLXNB2
3.62E-45	0.58039283	0.257	0.036	5.96E-41	Collecting duct principal cells	HOXB7
1.17E-44	1.0826664	0.618	0.202	1.92E-40	Collecting duct principal cells	SPINT2

3.21E-44	0.9070812	0.638	0.197	5.28E-40	Collecting duct principal cells	ZBTB20
3.55E-44	1.01958602	0.461	0.109	5.84E-40	Collecting duct principal cells	VMP1
9.42E-44	0.97893796	0.362	0.073	1.55E-39	Collecting duct principal cells	RTN3
2.62E-43	1.17209309	0.882	0.537	4.31E-39	Collecting duct principal cells	S100A11
2.78E-43	0.86179497	0.296	0.05	4.58E-39	Collecting duct principal cells	FOSL2
4.03E-43	0.55826344	0.276	0.043	6.63E-39	Collecting duct principal cells	PTBP3
4.07E-43	1.36642386	0.816	0.386	6.70E-39	Collecting duct principal cells	IGFBP7
1.91E-42	0.82318603	0.329	0.061	3.14E-38	Collecting duct principal cells	ATP1B3
2.50E-42	1.11736611	0.796	0.394	4.12E-38	Collecting duct principal cells	CD24
3.44E-42	0.72666161	0.355	0.07	5.66E-38	Collecting duct principal cells	AKAP9
3.89E-42	0.66246965	0.355	0.069	6.40E-38	Collecting duct principal cells	CD46
3.32E-41	0.76858486	0.27	0.044	5.48E-37	Collecting duct principal cells	DUSP6
8.81E-41	1.05002966	0.849	0.422	1.45E-36	Collecting duct principal cells	ACTG1
1.58E-40	0.9268435	0.553	0.166	2.61E-36	Collecting duct principal cells	TSC22D1
3.40E-40	0.7504637	0.414	0.095	5.60E-36	Collecting duct principal cells	ITGB1
8.31E-40	0.75104525	0.375	0.081	1.37E-35	Collecting duct principal cells	LRRFIP1
4.53E-39	0.58128902	0.27	0.045	7.47E-35	Collecting duct principal cells	EFCAB14
6.68E-38	0.71454978	0.362	0.079	1.10E-33	Collecting duct principal cells	SCCPDH
1.10E-37	0.85857523	0.493	0.136	1.81E-33	Collecting duct principal cells	N4BP2L2
1.83E-37	0.66063971	0.27	0.047	3.01E-33	Collecting duct principal cells	GSN
1.97E-37	0.85821685	0.329	0.068	3.24E-33	Collecting duct principal cells	B4GALT1
3.52E-36	0.68049868	0.322	0.066	5.81E-32	Collecting duct principal cells	HACD3
4.02E-36	0.67569282	0.329	0.068	6.62E-32	Collecting duct principal cells	CTNND1
4.18E-36	0.99994334	0.776	0.359	6.88E-32	Collecting duct principal cells	SEC62
1.47E-35	0.66697213	0.276	0.051	2.42E-31	Collecting duct principal cells	SPINT1
7.39E-35	0.64871421	0.283	0.054	1.22E-30	Collecting duct principal cells	MUC20-OT1
8.44E-35	0.6678239	0.368	0.086	1.39E-30	Collecting duct principal cells	PTTG1IP
6.01E-34	1.23766696	0.651	0.275	9.90E-30	Collecting duct principal cells	ATP1A1
1.14E-33	0.71771267	0.382	0.093	1.89E-29	Collecting duct principal cells	NTN4
2.30E-33	0.5342913	0.355	0.077	3.79E-29	Collecting duct principal cells	S100A4
3.42E-32	0.6715682	0.329	0.074	5.64E-28	Collecting duct principal cells	TM9SF3
9.73E-32	0.98882689	0.776	0.403	1.60E-27	Collecting duct principal cells	S100A10
9.93E-32	0.73962441	0.388	0.101	1.64E-27	Collecting duct principal cells	TGOLN2
2.30E-31	0.80402238	0.388	0.103	3.79E-27	Collecting duct principal cells	KCNJ16
7.22E-31	0.6511018	0.342	0.082	1.19E-26	Collecting duct principal cells	CREM
9.86E-31	0.60782341	0.276	0.056	1.62E-26	Collecting duct principal cells	SERPING1
1.20E-30	0.71332906	1	0.902	1.98E-26	Collecting duct principal cells	MT-CO2
2.11E-30	0.8096523	0.592	0.22	3.48E-26	Collecting duct principal cells	EIF5B
3.02E-30	0.79152273	0.336	0.081	4.98E-26	Collecting duct principal cells	SMIM22
2.99E-29	0.55904905	0.388	0.102	4.92E-25	Collecting duct principal cells	IFITM2
6.22E-28	0.87934597	0.401	0.121	1.02E-23	Collecting duct principal cells	AOC1
1.03E-27	0.89926216	0.329	0.084	1.69E-23	Collecting duct principal cells	GDF15
1.09E-27	0.84569629	0.546	0.206	1.79E-23	Collecting duct principal cells	AIF1L
4.30E-27	0.51868332	0.283	0.064	7.09E-23	Collecting duct principal cells	CCND1
4.63E-27	0.87543152	0.48	0.171	7.63E-23	Collecting duct principal cells	ZFP36L1
2.64E-26	0.62806818	0.336	0.089	4.35E-22	Collecting duct principal cells	SELENOS
4.43E-26	0.59258164	0.322	0.081	7.29E-22	Collecting duct principal cells	DUSP1
5.05E-26	0.82135179	0.342	0.093	8.32E-22	Collecting duct principal cells	SYNE2
5.25E-26	0.53761138	0.257	0.056	8.65E-22	Collecting duct principal cells	SUMF2
1.34E-25	0.76384787	0.638	0.281	2.21E-21	Collecting duct principal cells	CTSD
1.47E-25	0.85592182	0.566	0.238	2.41E-21	Collecting duct principal cells	APLP2
2.04E-25	0.64268613	1	0.853	3.36E-21	Collecting duct principal cells	MT-ND4
4.02E-25	0.75836583	0.48	0.173	6.62E-21	Collecting duct principal cells	CAPG
8.53E-25	0.71108413	0.257	0.06	1.40E-20	Collecting duct principal cells	PAPPA
8.54E-25	0.80757622	0.428	0.144	1.41E-20	Collecting duct principal cells	PAX8
1.05E-24	0.61713827	0.257	0.059	1.73E-20	Collecting duct principal cells	PABPN1
1.08E-24	0.82595906	0.329	0.092	1.78E-20	Collecting duct principal cells	NDRG1
1.78E-24	0.65619661	0.414	0.132	2.93E-20	Collecting duct principal cells	SRRM2

3.38E-24	0.75237968	0.447	0.154	5.56E-20	Collecting duct principal cells	SELENOM
5.47E-24	0.65946394	0.283	0.072	9.02E-20	Collecting duct principal cells	CYFIP2
8.54E-24	0.50991496	0.283	0.07	1.41E-19	Collecting duct principal cells	STAT3
1.01E-23	0.58506617	0.316	0.086	1.66E-19	Collecting duct principal cells	SSR1
4.04E-23	0.55443659	0.336	0.095	6.66E-19	Collecting duct principal cells	PNISR
7.88E-23	0.57232161	0.303	0.082	1.30E-18	Collecting duct principal cells	COL18A1
1.26E-22	0.77784908	0.421	0.146	2.08E-18	Collecting duct principal cells	ELF3
2.22E-22	0.56911285	0.368	0.113	3.66E-18	Collecting duct principal cells	MYO6
2.22E-22	1.09530726	0.789	0.488	3.66E-18	Collecting duct principal cells	ATP1B1
3.22E-22	0.79805017	0.487	0.187	5.30E-18	Collecting duct principal cells	EPCAM
7.02E-22	0.61164358	0.645	0.296	1.16E-17	Collecting duct principal cells	PSAP
8.53E-22	0.7182537	0.507	0.201	1.41E-17	Collecting duct principal cells	ARL6IP1
1.17E-21	0.82338526	0.461	0.176	1.93E-17	Collecting duct principal cells	GNAS
1.77E-20	0.53697388	0.303	0.087	2.91E-16	Collecting duct principal cells	MPHOSPH8
2.03E-20	0.63099327	0.809	0.513	3.35E-16	Collecting duct principal cells	CD63
2.53E-20	0.77765463	0.454	0.178	4.18E-16	Collecting duct principal cells	METAP2
5.52E-20	0.68024691	0.474	0.191	9.10E-16	Collecting duct principal cells	HIF1A
6.03E-20	0.55369549	0.618	0.273	9.93E-16	Collecting duct principal cells	HLA-C
1.64E-19	0.56511213	0.395	0.138	2.70E-15	Collecting duct principal cells	GRN
1.79E-19	0.4727657	0.987	0.772	2.95E-15	Collecting duct principal cells	MT-ND1
1.64E-18	0.63072818	0.382	0.137	2.71E-14	Collecting duct principal cells	CD151
1.88E-18	0.62058454	0.27	0.078	3.10E-14	Collecting duct principal cells	GPRC5C
1.89E-18	0.70864437	0.447	0.182	3.11E-14	Collecting duct principal cells	MRPS6
3.21E-18	0.62916521	0.355	0.124	5.29E-14	Collecting duct principal cells	TNFRSF12A
3.37E-18	0.59996423	0.434	0.17	5.56E-14	Collecting duct principal cells	REEP5
3.58E-18	0.65111516	0.678	0.359	5.90E-14	Collecting duct principal cells	TSPAN1
4.70E-18	0.75574827	0.5	0.22	7.73E-14	Collecting duct principal cells	PPP1CB
5.44E-18	0.65863845	0.368	0.135	8.96E-14	Collecting duct principal cells	SRSF11
5.70E-18	0.50189944	0.388	0.138	9.39E-14	Collecting duct principal cells	LMNA
5.87E-18	0.60876041	0.467	0.193	9.68E-14	Collecting duct principal cells	KRTCAP2
7.80E-18	0.52487362	0.961	0.797	1.29E-13	Collecting duct principal cells	MT-ND3
8.77E-18	0.42150128	0.283	0.084	1.44E-13	Collecting duct principal cells	SNHG9
1.13E-17	0.56583406	0.546	0.243	1.87E-13	Collecting duct principal cells	SON
1.46E-17	0.44690804	0.276	0.082	2.41E-13	Collecting duct principal cells	CHD9
5.88E-17	0.53124784	0.316	0.105	9.69E-13	Collecting duct principal cells	JAK1
8.12E-17	0.42709798	0.954	0.822	1.34E-12	Collecting duct principal cells	ACTB
9.10E-17	0.50048089	0.408	0.155	1.50E-12	Collecting duct principal cells	SDC4
9.32E-17	0.52749017	0.388	0.146	1.54E-12	Collecting duct principal cells	TMEM50A
1.66E-16	0.46765787	1	0.891	2.73E-12	Collecting duct principal cells	MT-CO1
1.70E-16	0.56709201	0.618	0.323	2.80E-12	Collecting duct principal cells	DDX5
2.37E-16	0.4870928	0.283	0.09	3.90E-12	Collecting duct principal cells	TINAGL1
2.86E-16	0.52557381	0.342	0.123	4.71E-12	Collecting duct principal cells	ACTN4
3.72E-16	0.49575021	0.263	0.081	6.13E-12	Collecting duct principal cells	TMEM123
9.22E-16	0.42116573	0.263	0.081	1.52E-11	Collecting duct principal cells	SERINC2
1.06E-15	0.48857854	0.441	0.185	1.75E-11	Collecting duct principal cells	LAMP1
1.47E-15	0.50504407	0.27	0.087	2.42E-11	Collecting duct principal cells	ARGLU1
1.88E-15	0.44049642	0.322	0.112	3.10E-11	Collecting duct principal cells	AES
2.33E-15	0.63306382	0.362	0.142	3.85E-11	Collecting duct principal cells	RCAN1
3.90E-15	0.52822245	0.461	0.205	6.43E-11	Collecting duct principal cells	PTP4A2
5.88E-15	0.41329298	0.257	0.08	9.68E-11	Collecting duct principal cells	11-Sep
5.88E-15	0.62711662	0.48	0.216	9.69E-11	Collecting duct principal cells	TXNIP
7.29E-15	0.44389983	0.349	0.129	1.20E-10	Collecting duct principal cells	IFI27L2
1.16E-14	0.526955	0.289	0.101	1.91E-10	Collecting duct principal cells	XBP1
1.30E-14	0.54308325	0.289	0.101	2.14E-10	Collecting duct principal cells	HNRNPH1
1.70E-14	0.39173663	1	0.821	2.80E-10	Collecting duct principal cells	MT-CYB
1.81E-14	0.54438188	0.276	0.095	2.99E-10	Collecting duct principal cells	COMT
2.24E-14	0.47481513	0.257	0.084	3.70E-10	Collecting duct principal cells	IP6K2
2.34E-14	0.55123977	0.296	0.105	3.85E-10	Collecting duct principal cells	IVNS1ABP

3.34E-14	0.55026607	0.822	0.61	5.50E-10	Collecting duct principal cells	H3F3B
3.88E-14	0.49925006	0.27	0.091	6.39E-10	Collecting duct principal cells	ARHGAP29
7.11E-14	0.50437944	0.441	0.193	1.17E-09	Collecting duct principal cells	CALR
9.41E-14	0.59894812	0.671	0.445	1.55E-09	Collecting duct principal cells	RTN4
1.37E-13	0.54851154	0.395	0.17	2.26E-09	Collecting duct principal cells	TRAM1
1.43E-13	0.49174826	0.296	0.11	2.35E-09	Collecting duct principal cells	AAK1
1.47E-13	0.60038836	0.533	0.283	2.42E-09	Collecting duct principal cells	ASAH1
1.50E-13	0.62293674	0.447	0.212	2.47E-09	Collecting duct principal cells	JPT1
1.70E-13	0.56445669	0.592	0.309	2.80E-09	Collecting duct principal cells	HLA-A
1.71E-13	0.48956911	0.5	0.236	2.81E-09	Collecting duct principal cells	HLA-B
2.12E-13	0.39099541	1	0.892	3.50E-09	Collecting duct principal cells	MT-CO3
2.15E-13	0.5257591	0.303	0.114	3.55E-09	Collecting duct principal cells	NFE2L2
3.34E-13	0.57943712	0.487	0.241	5.51E-09	Collecting duct principal cells	C9orf16
4.96E-13	0.39267036	0.987	0.826	8.17E-09	Collecting duct principal cells	MT-ND2
6.39E-13	0.45746352	0.388	0.167	1.05E-08	Collecting duct principal cells	TMCO1
9.88E-13	0.33183055	0.987	0.833	1.63E-08	Collecting duct principal cells	MT-ATP6
1.51E-12	0.37180376	0.941	0.793	2.48E-08	Collecting duct principal cells	ITM2B
1.74E-12	0.52571303	0.414	0.194	2.87E-08	Collecting duct principal cells	YWHAZ
2.29E-12	0.40646502	0.329	0.132	3.77E-08	Collecting duct principal cells	HNRNPM
6.94E-12	0.39176146	0.434	0.202	1.14E-07	Collecting duct principal cells	CANX
8.37E-12	0.45206887	0.454	0.224	1.38E-07	Collecting duct principal cells	PRKAR1A
8.93E-12	0.48977756	0.375	0.17	1.47E-07	Collecting duct principal cells	HNRNPU
9.39E-12	0.35114115	0.974	0.898	1.55E-07	Collecting duct principal cells	PTMA
1.04E-11	0.40144511	0.309	0.122	1.72E-07	Collecting duct principal cells	MTDH
1.12E-11	0.45828715	0.474	0.237	1.85E-07	Collecting duct principal cells	TMBIM4
1.33E-11	0.49726976	0.349	0.154	2.19E-07	Collecting duct principal cells	TMED10
1.46E-11	0.5173397	0.395	0.182	2.41E-07	Collecting duct principal cells	ANXA11
2.11E-11	0.49792865	0.309	0.123	3.48E-07	Collecting duct principal cells	MT-ND6
2.14E-11	0.41108553	0.375	0.166	3.52E-07	Collecting duct principal cells	CTTN
2.29E-11	0.45606211	0.599	0.341	3.77E-07	Collecting duct principal cells	NUCKS1
4.19E-11	0.35038326	0.276	0.107	6.90E-07	Collecting duct principal cells	DDX46
4.38E-11	0.42267384	0.336	0.145	7.21E-07	Collecting duct principal cells	TMED4
5.71E-11	0.49731486	0.487	0.253	9.40E-07	Collecting duct principal cells	HSP90B1
7.08E-11	0.41816401	0.414	0.199	1.17E-06	Collecting duct principal cells	LEPROT
8.36E-11	0.33425644	0.276	0.107	1.38E-06	Collecting duct principal cells	FUS
9.85E-11	0.5090537	0.382	0.185	1.62E-06	Collecting duct principal cells	EMX2
1.04E-10	0.55291937	0.664	0.44	1.71E-06	Collecting duct principal cells	KRT18
1.28E-10	0.48517091	0.612	0.378	2.12E-06	Collecting duct principal cells	TMEM59
1.60E-10	0.40409598	0.27	0.107	2.63E-06	Collecting duct principal cells	HNRNPD
1.77E-10	0.3882039	0.355	0.161	2.92E-06	Collecting duct principal cells	PDIA6
1.97E-10	0.44070224	0.303	0.128	3.24E-06	Collecting duct principal cells	CSNK1A1
2.32E-10	0.47024099	0.632	0.387	3.81E-06	Collecting duct principal cells	S100A13
3.86E-10	0.48646757	0.329	0.141	6.35E-06	Collecting duct principal cells	HLA-E
4.83E-10	0.74677528	0.388	0.2	7.96E-06	Collecting duct principal cells	NUPR1
4.96E-10	0.42660699	0.395	0.189	8.16E-06	Collecting duct principal cells	RBM39
5.25E-10	0.41603471	0.329	0.149	8.65E-06	Collecting duct principal cells	ZFAND5
6.27E-10	0.41935715	0.349	0.161	1.03E-05	Collecting duct principal cells	DPM3
7.24E-10	0.4677353	0.336	0.157	1.19E-05	Collecting duct principal cells	CAPNS1
7.33E-10	0.85248564	0.27	0.114	1.21E-05	Collecting duct principal cells	HES1
9.06E-10	0.39579123	0.888	0.619	1.49E-05	Collecting duct principal cells	MT-ND5
1.40E-09	0.38192141	0.276	0.114	2.30E-05	Collecting duct principal cells	SFPQ
1.44E-09	0.44722705	0.572	0.349	2.37E-05	Collecting duct principal cells	MRPL33
2.16E-09	0.37695954	0.296	0.13	3.55E-05	Collecting duct principal cells	GTF2I
2.58E-09	0.3191201	0.283	0.121	4.25E-05	Collecting duct principal cells	ZFP36L2
3.06E-09	0.39795107	0.599	0.352	5.05E-05	Collecting duct principal cells	MT-ND4L
3.94E-09	0.54673767	0.283	0.127	6.48E-05	Collecting duct principal cells	KLF6
4.22E-09	0.37091191	0.296	0.132	6.94E-05	Collecting duct principal cells	ATP6AP2
7.44E-09	0.36400167	0.684	0.475	0.0001225	Collecting duct principal cells	BEX3

7.73E-09	0.28567935	0.349	0.163	0.0001273	Collecting duct principal cells	ARG2
8.94E-09	0.38343586	0.257	0.109	0.0001472	Collecting duct principal cells	PARD6B
9.64E-09	0.33940387	0.309	0.141	0.0001587	Collecting duct principal cells	WDR1
1.07E-08	0.4052234	0.612	0.386	0.0001766	Collecting duct principal cells	NDUFC2
5.25E-08	0.35767632	0.349	0.176	0.0008642	Collecting duct principal cells	UBXN4
5.41E-08	0.33204527	0.257	0.113	0.0008903	Collecting duct principal cells	FAM133B
5.48E-08	0.38867644	0.612	0.405	0.000903	Collecting duct principal cells	CSTB
6.41E-08	0.31992763	0.362	0.184	0.0010556	Collecting duct principal cells	TMED2
7.19E-08	0.36033821	0.296	0.143	0.0011844	Collecting duct principal cells	LITAF
7.71E-08	0.3970115	0.263	0.121	0.0012705	Collecting duct principal cells	CCDC12
8.68E-08	0.37913443	0.625	0.394	0.0014294	Collecting duct principal cells	IFITM3
9.74E-08	0.27030393	0.441	0.235	0.0016036	Collecting duct principal cells	SDCBP
1.02E-07	0.30438884	0.539	0.321	0.0016819	Collecting duct principal cells	CD164
1.24E-07	0.39440619	0.408	0.226	0.002041	Collecting duct principal cells	VAMP2
1.33E-07	0.36525665	0.467	0.271	0.0021829	Collecting duct principal cells	PET100
1.84E-07	0.37542745	0.309	0.153	0.0030323	Collecting duct principal cells	PDIA3
1.85E-07	0.4072771	0.283	0.135	0.0030443	Collecting duct principal cells	JUNB
2.62E-07	0.27888059	0.908	0.715	0.0043187	Collecting duct principal cells	TMSB10
2.81E-07	0.32139503	0.336	0.171	0.0046309	Collecting duct principal cells	7-Sep
2.83E-07	0.35197256	0.408	0.219	0.0046655	Collecting duct principal cells	TSPO
3.44E-07	0.57169647	0.461	0.289	0.0056708	Collecting duct principal cells	TPM1
3.65E-07	0.32786345	0.263	0.125	0.0060052	Collecting duct principal cells	ACTR2
3.97E-07	0.29432013	0.276	0.133	0.0065311	Collecting duct principal cells	HSPA5
4.78E-07	0.36812845	0.454	0.278	0.007879	Collecting duct principal cells	PPIB
5.36E-07	0.29103798	0.572	0.363	0.0088264	Collecting duct principal cells	CTSH
5.42E-07	0.26114987	0.73	0.496	0.0089299	Collecting duct principal cells	TMBIM6
6.04E-07	0.3957598	0.408	0.233	0.0099542	Collecting duct principal cells	ATP6V0B
6.50E-07	0.3397801	0.789	0.571	0.0107116	Collecting duct principal cells	HMGB1
7.65E-07	0.33055575	0.355	0.192	0.0126031	Collecting duct principal cells	TCF25
7.97E-07	0.27982711	0.263	0.127	0.0131287	Collecting duct principal cells	TGIF1
8.82E-07	0.46481913	0.368	0.21	0.0145301	Collecting duct principal cells	CD59
1.10E-06	0.26710923	0.474	0.279	0.0181219	Collecting duct principal cells	BSG
1.13E-06	0.32066864	0.395	0.223	0.0186125	Collecting duct principal cells	SYPL1
1.53E-06	0.26392225	0.303	0.154	0.0252185	Collecting duct principal cells	ARPC1B
1.81E-06	0.33641365	0.428	0.256	0.0298941	Collecting duct principal cells	SRSF5
1.97E-06	0.29015535	0.296	0.153	0.0325172	Collecting duct principal cells	SUMO3
2.33E-06	0.31724975	0.382	0.21	0.0383903	Collecting duct principal cells	SRI
2.80E-06	0.27377432	0.296	0.155	0.0460521	Collecting duct principal cells	PPP2R1A
3.20E-06	0.29713266	0.743	0.552	0.0526353	Collecting duct principal cells	LAPTM4A
3.51E-06	0.32865657	0.5	0.316	0.0577698	Collecting duct principal cells	HMGN2
3.91E-06	0.2748626	0.263	0.133	0.0644669	Collecting duct principal cells	HP1BP3
4.06E-06	0.35283115	0.441	0.279	0.0668137	Collecting duct principal cells	BAG1
5.16E-06	0.42243202	0.316	0.175	0.0849532	Collecting duct principal cells	VIM
6.33E-06	0.32895994	0.526	0.351	0.1042837	Collecting duct principal cells	HNRNPC
6.51E-06	0.30151438	0.289	0.149	0.1073027	Collecting duct principal cells	YWHAH
8.03E-06	0.31040828	0.447	0.275	0.1322931	Collecting duct principal cells	NCL
1.11E-05	0.31791655	0.461	0.288	0.1834546	Collecting duct principal cells	FAM107B
1.22E-05	0.30376826	0.263	0.139	0.2008271	Collecting duct principal cells	EI24
1.57E-05	0.45671295	0.579	0.412	0.2591203	Collecting duct principal cells	KRT8
2.14E-05	0.28977176	0.375	0.222	0.3524246	Collecting duct principal cells	TAGLN2
2.61E-05	0.28570726	0.329	0.193	0.4300327	Collecting duct principal cells	HNRNPA3
2.64E-05	0.2666559	0.474	0.304	0.4345751	Collecting duct principal cells	SERP1
2.67E-05	0.25832959	0.533	0.364	0.4391494	Collecting duct principal cells	SSR4
2.74E-05	0.28648914	0.303	0.168	0.4520911	Collecting duct principal cells	H2AFV
2.75E-05	0.28996378	0.263	0.141	0.4522986	Collecting duct principal cells	C5orf24
2.76E-05	0.29203104	0.434	0.272	0.4546455	Collecting duct principal cells	TCEAL9
3.02E-05	0.30987387	0.48	0.32	0.4979625	Collecting duct principal cells	HIST1H4C
3.34E-05	0.35186766	0.421	0.278	0.5509476	Collecting duct principal cells	SPCS2

3.52E-05	0.27781406	0.263	0.144	0.580189	Collecting duct principal cells	PPIG
3.80E-05	0.29829634	0.658	0.509	0.6262356	Collecting duct principal cells	ATP6V0E1
4.43E-05	0.31760873	0.388	0.246	0.7300577	Collecting duct principal cells	YBX3
4.59E-05	0.33723301	0.296	0.174	0.7558335	Collecting duct principal cells	CAST
5.09E-05	0.37213834	0.408	0.265	0.8375777	Collecting duct principal cells	FOLR1
7.28E-05	0.25658012	0.322	0.192	1	Collecting duct principal cells	NDFIP1
0.000165	0.2624986	0.75	0.639	1	Collecting duct principal cells	CALM2
0.000214	0.26756663	0.283	0.17	1	Collecting duct principal cells	NME3
0.001129	0.25302781	0.717	0.599	1	Collecting duct principal cells	ENO1
0.001208	0.29842823	0.257	0.161	1	Collecting duct principal cells	CHMP1B
0.007334	0.39568201	0.48	0.408	1	Collecting duct principal cells	GLS
0	4.8322007	0.697	0.01	0	B cells	IGKC
0	3.38914666	0.577	0.001	0	B cells	IGHM
0	2.72136341	0.845	0.033	0	B cells	LTB
0	2.43220802	0.768	0.001	0	B cells	CD79A
0	2.430869	0.739	0.006	0	B cells	CD79B
0	2.41363845	0.915	0.059	0	B cells	CD52
0	2.27966679	0.817	0.038	0	B cells	HLA-DQB1
0	2.27311518	0.782	0.036	0	B cells	CD37
0	2.21843126	0.697	0.027	0	B cells	CXCR4
0	2.15485572	0.662	0.001	0	B cells	MS4A1
0	2.09289242	0.359	0.001	0	B cells	JCHAIN
0	1.72748141	0.592	0.016	0	B cells	HLA-DQA1
0	1.69103123	0.542	0.002	0	B cells	VPREB3
0	1.68622058	0.338	0	0	B cells	TCL1A
0	1.61925486	0.479	0.002	0	B cells	LINC00926
0	1.49259076	0.324	0	0	B cells	IGHD
0	1.39515757	0.458	0.013	0	B cells	SELL
0	1.35714592	0.43	0.005	0	B cells	BANK1
0	1.31537732	0.451	0.013	0	B cells	NCF1
0	1.27210621	0.43	0.012	0	B cells	MEF2C
0	1.17053615	0.373	0.003	0	B cells	HVCN1
0	1.07162585	0.289	0.006	0	B cells	FCMR
0	0.99930157	0.261	0.002	0	B cells	BCL11A
0	0.98448948	0.254	0	0	B cells	FCRLA
0	0.92714444	0.254	0.004	0	B cells	CCR7
3.41E-279	1.71988111	0.662	0.042	5.62E-275	B cells	LAPTM5
3.33E-275	2.15200643	0.852	0.076	5.48E-271	B cells	HLA-DPA1
1.65E-265	1.48779619	0.592	0.034	2.72E-261	B cells	LIMD2
7.48E-248	0.97434966	0.254	0.006	1.23E-243	B cells	ADAM28
1.90E-233	2.26470565	0.88	0.099	3.13E-229	B cells	HLA-DPB1
1.64E-225	1.02810747	0.268	0.008	2.69E-221	B cells	RNASE6
8.83E-218	0.88557575	0.254	0.007	1.45E-213	B cells	LYL1
5.16E-217	1.57320913	0.711	0.06	8.51E-213	B cells	ARHGDIB
1.46E-209	1.35086091	0.415	0.022	2.40E-205	B cells	HLA-DQA2
1.81E-208	1.18116593	0.373	0.017	2.99E-204	B cells	HLA-DMB
2.86E-187	1.26517985	0.451	0.028	4.71E-183	B cells	BIRC3
2.16E-156	1.35537613	0.556	0.05	3.55E-152	B cells	CORO1A
4.55E-143	1.14856971	0.437	0.034	7.49E-139	B cells	GPSM3
4.41E-141	1.13822759	0.415	0.031	7.26E-137	B cells	CD53
3.02E-139	0.82775389	0.261	0.013	4.98E-135	B cells	RALGPS2
4.25E-136	0.9840398	0.261	0.013	7.00E-132	B cells	ID3
5.07E-135	1.42159581	0.542	0.058	8.36E-131	B cells	HLA-DRB5
1.04E-130	1.27434529	0.373	0.027	1.71E-126	B cells	PLAC8
2.57E-128	1.01031763	0.401	0.032	4.24E-124	B cells	RAC2
7.15E-126	0.95094682	0.324	0.021	1.18E-121	B cells	EVI2B
3.45E-124	2.71282179	0.965	0.289	5.68E-120	B cells	HLA-DRA
1.86E-122	2.07273583	0.93	0.231	3.06E-118	B cells	HLA-DRB1

2.48E-122	1.03932688	0.338	0.024	4.08E-118	B cells	IFI16
2.91E-119	1.37080952	0.563	0.069	4.79E-115	B cells	HLA-DMA
3.62E-119	0.87771431	0.43	0.038	5.96E-115	B cells	CTSS
1.22E-116	1.30561552	0.451	0.044	2.01E-112	B cells	FXYD5
1.63E-115	1.04956268	0.472	0.048	2.69E-111	B cells	LSP1
1.45E-107	0.86118191	0.289	0.02	2.39E-103	B cells	RIPOR2
9.11E-106	0.80567643	0.268	0.018	1.50E-101	B cells	SWAP70
2.39E-104	1.12887648	0.275	0.019	3.94E-100	B cells	CD69
1.50E-102	0.86239739	0.282	0.02	2.47E-98	B cells	ACAP1
1.89E-100	1.63990946	1	0.298	3.12E-96	B cells	MALAT1
2.88E-99	2.6986632	1	0.497	4.74E-95	B cells	CD74
3.14E-98	1.52055337	0.859	0.209	5.18E-94	B cells	BTG1
9.11E-98	1.02195805	0.38	0.038	1.50E-93	B cells	TNFAIP8
2.74E-95	1.03713037	0.43	0.049	4.51E-91	B cells	RNASET2
1.90E-90	0.82733953	0.275	0.021	3.12E-86	B cells	IL2RG
2.19E-90	0.9349123	0.31	0.027	3.61E-86	B cells	UCP2
2.50E-90	1.13194265	0.303	0.026	4.12E-86	B cells	KLF2
3.77E-88	0.96791656	0.408	0.046	6.21E-84	B cells	PTPRC
1.21E-87	1.60994914	1	0.389	2.00E-83	B cells	TMSB4X
4.00E-85	1.37164466	0.866	0.234	6.59E-81	B cells	HLA-B
1.28E-84	0.99136722	0.303	0.028	2.11E-80	B cells	STK17A
1.57E-82	0.89374869	0.289	0.026	2.59E-78	B cells	ISG20
2.05E-82	1.55609674	0.549	0.093	3.38E-78	B cells	JUN
4.89E-82	0.89140021	0.317	0.031	8.05E-78	B cells	TRAF3IP3
3.97E-81	1.32927908	0.669	0.139	6.54E-77	B cells	HLA-E
1.24E-80	0.75088066	0.254	0.02	2.04E-76	B cells	DCK
7.23E-79	0.87566439	0.324	0.034	1.19E-74	B cells	PTPN6
4.52E-78	1.02850401	0.43	0.058	7.45E-74	B cells	POLR2J3.1
1.92E-76	0.78395966	0.268	0.024	3.16E-72	B cells	SMAP2
3.92E-72	1.37127828	1	0.867	6.46E-68	B cells	RPS2
2.63E-71	1.33781316	1	0.922	4.34E-67	B cells	RPS19
4.08E-71	1.21516279	0.993	0.971	6.73E-67	B cells	RPS27
3.08E-70	1.05911254	1	0.956	5.08E-66	B cells	RPLP2
6.96E-70	1.07452527	1	0.981	1.15E-65	B cells	RPS18
4.71E-69	1.14828218	1	0.935	7.76E-65	B cells	RPL18A
2.19E-68	0.8754513	1	0.99	3.60E-64	B cells	RPL13A
2.55E-65	1.11761563	1	0.858	4.21E-61	B cells	RPL39
1.50E-63	0.8117028	1	0.992	2.48E-59	B cells	RPL10
1.69E-63	1.12715338	1	0.822	2.78E-59	B cells	B2M
3.29E-63	0.86021284	1	0.989	5.42E-59	B cells	RPL13
2.24E-62	0.78121852	1	0.987	3.68E-58	B cells	RPL32
1.72E-61	1.15356523	0.986	0.851	2.83E-57	B cells	RPS3
2.89E-61	1.29320956	0.732	0.215	4.76E-57	B cells	TXNIP
5.42E-59	0.74899688	1	0.98	8.93E-55	B cells	RPS27A
9.11E-59	0.78184532	0.296	0.037	1.50E-54	B cells	STK4
1.86E-57	0.90979715	1	0.932	3.07E-53	B cells	RPL27A
4.50E-57	1.03165062	0.993	0.821	7.40E-53	B cells	RPL18
1.19E-55	0.92059762	1	0.956	1.96E-51	B cells	RPL21
2.73E-55	0.84592217	1	0.925	4.49E-51	B cells	RPL28
4.22E-55	0.75410458	0.282	0.035	6.95E-51	B cells	CD48
1.02E-53	0.83427611	1	0.959	1.68E-49	B cells	RPS15A
1.92E-53	0.80011465	0.993	0.956	3.17E-49	B cells	RPS8
3.39E-53	0.74152823	0.993	0.973	5.58E-49	B cells	RPS12
5.64E-53	1.00127205	0.465	0.094	9.30E-49	B cells	PNISR
6.65E-52	0.70832181	1	0.976	1.09E-47	B cells	RPL11
3.58E-51	1.04235189	0.782	0.272	5.90E-47	B cells	HLA-C
1.05E-49	0.60664141	1	0.994	1.73E-45	B cells	RPL34
3.96E-49	0.89567804	0.324	0.049	6.53E-45	B cells	GMFG

4.03E-49	0.78628386	1	0.938	6.63E-45	B cells	RPL23A
4.75E-49	1.26304986	0.535	0.133	7.83E-45	B cells	JUNB
1.77E-48	0.71959943	0.359	0.059	2.92E-44	B cells	EMP3
2.29E-48	0.92518356	0.993	0.894	3.78E-44	B cells	RPS29
3.47E-48	0.86902672	1	0.878	5.71E-44	B cells	RPL30
3.62E-47	0.67986864	0.993	0.978	5.96E-43	B cells	RPS14
3.87E-46	0.62229515	1	0.99	6.37E-42	B cells	RPLP1
6.96E-46	0.85945908	0.972	0.879	1.15E-41	B cells	RPS5
6.43E-44	0.5755874	1	0.997	1.06E-39	B cells	RPL41
1.80E-42	0.68053232	1	0.952	2.96E-38	B cells	RPL8
5.36E-42	1.06057915	0.754	0.308	8.83E-38	B cells	HLA-A
2.37E-41	0.53718185	1	0.983	3.90E-37	B cells	RPS15
7.63E-41	1.00463037	0.394	0.085	1.26E-36	B cells	IER2
3.41E-40	0.76007026	0.979	0.887	5.62E-36	B cells	RPL10A
3.84E-40	0.70867575	0.986	0.905	6.33E-36	B cells	FAU
4.07E-40	0.5821195	1	0.984	6.70E-36	B cells	RPS6
2.62E-39	0.56584014	1	0.979	4.32E-35	B cells	RPS23
5.72E-39	0.63556212	0.986	0.963	9.41E-35	B cells	RPS3A
6.80E-39	0.63406034	1	0.941	1.12E-34	B cells	RPL15
7.32E-39	0.91971849	0.944	0.704	1.21E-34	B cells	RPSA
1.11E-38	0.60959613	1	0.963	1.82E-34	B cells	RPS25
1.13E-37	1.12460726	0.373	0.081	1.87E-33	B cells	DUSP1
6.77E-37	0.84089573	0.951	0.711	1.11E-32	B cells	RPS21
6.79E-37	0.62756674	0.993	0.922	1.12E-32	B cells	RPS9
9.20E-37	0.65068052	0.268	0.044	1.52E-32	B cells	EVL
1.61E-36	0.55707349	0.993	0.972	2.64E-32	B cells	RPL3
2.07E-35	0.51356322	1	0.98	3.41E-31	B cells	RPS28
2.77E-33	0.87941183	0.732	0.322	4.57E-29	B cells	DDX5
4.12E-33	0.59330141	1	0.954	6.79E-29	B cells	RPL35A
4.79E-33	0.94153483	0.88	0.599	7.88E-29	B cells	RPS10
1.21E-31	0.564567	0.993	0.942	2.00E-27	B cells	RPS4X
3.68E-31	0.68039379	0.958	0.82	6.07E-27	B cells	RPS11
6.71E-31	0.77338686	0.965	0.822	1.11E-26	B cells	ACTB
8.45E-31	0.58073919	0.993	0.937	1.39E-26	B cells	RPL36
1.39E-30	0.57547506	0.986	0.943	2.29E-26	B cells	RPS13
1.52E-30	0.89492983	0.732	0.371	2.50E-26	B cells	PABPC1
1.74E-30	0.93125963	0.338	0.081	2.87E-26	B cells	TSC22D3
3.92E-30	0.5053675	0.993	0.946	6.46E-26	B cells	RPL35
7.46E-30	0.70571962	0.345	0.082	1.23E-25	B cells	PSMB9
1.34E-29	0.54120292	0.993	0.937	2.20E-25	B cells	RPL9
3.90E-29	0.71004436	0.275	0.055	6.43E-25	B cells	FOS
4.39E-29	0.71102024	0.275	0.057	7.24E-25	B cells	TAPBP
6.02E-29	0.78339033	0.444	0.134	9.91E-25	B cells	SNX2
8.63E-29	0.88160981	0.275	0.058	1.42E-24	B cells	H1FX
5.61E-27	0.73416119	0.915	0.568	9.24E-23	B cells	CYBA
5.08E-26	0.49571285	1	0.924	8.36E-22	B cells	RPS20
2.49E-25	0.80624965	0.331	0.089	4.10E-21	B cells	DNAJB1
3.53E-25	0.53082615	0.986	0.898	5.81E-21	B cells	PTMA
5.12E-25	0.76524396	0.289	0.07	8.43E-21	B cells	HSPA1B
3.93E-24	0.87805227	0.697	0.359	6.48E-20	B cells	NOP53
1.37E-23	0.60541878	0.254	0.058	2.25E-19	B cells	KMT2E
3.63E-23	0.37859562	1	0.969	5.98E-19	B cells	RPL7
1.10E-22	0.4407148	1	0.977	1.81E-18	B cells	RPL19
1.72E-22	0.53649633	0.972	0.715	2.84E-18	B cells	TMSB10
2.44E-22	0.46702655	0.979	0.931	4.02E-18	B cells	RPS16
3.21E-22	0.59748176	0.254	0.06	5.29E-18	B cells	WSB1
8.18E-22	0.4336872	0.993	0.959	1.35E-17	B cells	RPL31
8.68E-22	0.48956489	0.986	0.871	1.43E-17	B cells	RPL27

1.25E-21	0.48632201	0.993	0.913	2.05E-17	B cells	RPL37
3.15E-21	0.619122	0.359	0.111	5.19E-17	B cells	PSMB8
6.44E-20	0.59157696	0.261	0.068	1.06E-15	B cells	ANXA6
2.66E-19	0.45303746	0.338	0.102	4.38E-15	B cells	IFITM2
5.20E-19	0.60633923	0.254	0.067	8.56E-15	B cells	PARP1
4.02E-18	0.37393819	0.993	0.965	6.62E-14	B cells	RPL26
7.66E-18	0.604	0.345	0.118	1.26E-13	B cells	ARL6IP5
2.93E-17	0.41140906	1	0.963	4.82E-13	B cells	RPL12
3.72E-17	0.61099063	0.282	0.087	6.12E-13	B cells	ARGLU1
5.87E-17	0.62659518	0.303	0.098	9.67E-13	B cells	DRAM2
6.78E-17	0.41814447	0.965	0.872	1.12E-12	B cells	RACK1
1.10E-16	0.49467739	0.923	0.764	1.81E-12	B cells	RPL4
2.18E-16	0.69149328	0.563	0.284	3.58E-12	B cells	NAP1L1
1.59E-15	0.36004023	0.972	0.899	2.62E-11	B cells	UBA52
4.00E-15	0.76162168	0.845	0.61	6.59E-11	B cells	H3F3B
4.29E-15	0.47220445	0.944	0.767	7.06E-11	B cells	EEF1D
2.66E-14	0.71357372	0.275	0.092	4.37E-10	B cells	ZFP36
2.98E-14	0.64963403	0.324	0.121	4.91E-10	B cells	ZFP36L2
3.14E-14	0.54838213	0.352	0.137	5.18E-10	B cells	N4BP2L2
3.96E-14	0.32054612	0.993	0.94	6.52E-10	B cells	RPL29
4.82E-14	0.55018332	0.317	0.116	7.95E-10	B cells	YPEL3
5.36E-14	0.41262707	0.93	0.87	8.83E-10	B cells	RPL5
5.89E-14	0.57029791	0.345	0.136	9.70E-10	B cells	SRSF7
7.42E-14	0.47838093	0.289	0.1	1.22E-09	B cells	SRSF10
2.65E-13	0.46819258	0.261	0.088	4.37E-09	B cells	RNPS1
7.06E-13	0.49883873	0.838	0.672	1.16E-08	B cells	EEF1B2
7.78E-13	0.44782587	0.923	0.821	1.28E-08	B cells	RPLP0
1.50E-12	0.61202182	0.331	0.135	2.47E-08	B cells	SRSF11
4.89E-12	0.59516254	0.38	0.171	8.05E-08	B cells	7-Sep
9.92E-12	0.53258302	0.282	0.107	1.63E-07	B cells	FUS
1.46E-11	0.59310652	0.43	0.21	2.40E-07	B cells	HERPUD1
2.89E-11	0.28124181	0.993	0.959	4.76E-07	B cells	RPS7
2.93E-11	0.51451343	0.669	0.423	4.82E-07	B cells	ACTG1
4.54E-11	0.33286233	0.986	0.931	7.48E-07	B cells	RPL6
4.81E-11	0.76877452	0.31	0.136	7.93E-07	B cells	SMIM14
1.76E-10	0.47639662	0.521	0.298	2.90E-06	B cells	EIF3F
2.23E-10	0.50964947	0.479	0.259	3.67E-06	B cells	CNBP
2.64E-10	0.50403595	0.437	0.226	4.35E-06	B cells	VAMP2
1.45E-09	0.55820551	0.373	0.19	2.39E-05	B cells	RBM39
1.55E-09	0.44275904	0.254	0.103	2.55E-05	B cells	TAF7
2.36E-09	0.46880041	0.373	0.183	3.89E-05	B cells	EIF3D
2.41E-09	0.38550382	0.676	0.448	3.96E-05	B cells	SH3BGRL3
3.66E-09	0.31065229	0.986	0.949	6.02E-05	B cells	RPL37A
4.91E-09	0.310292	0.972	0.846	8.08E-05	B cells	PFDN5
6.92E-09	0.41126971	0.69	0.468	0.0001139	B cells	RPL36A
8.12E-09	0.42205653	0.352	0.171	0.0001338	B cells	ACTR3
1.21E-08	0.50116597	0.535	0.332	0.0001997	B cells	ZFAS1
2.31E-08	0.41340098	0.401	0.217	0.0003802	B cells	TPM3
2.79E-08	0.26555588	0.979	0.882	0.0004593	B cells	RPL22
2.81E-08	0.41108613	0.697	0.479	0.0004636	B cells	RPL17
3.70E-08	0.53365601	0.268	0.122	0.0006099	B cells	MTDH
6.13E-08	0.42068257	0.345	0.177	0.0010105	B cells	SELENOF
6.89E-08	0.36369927	0.739	0.566	0.0011346	B cells	ARPC3
6.91E-08	0.31338755	0.958	0.869	0.0011387	B cells	RPL38
7.92E-08	0.36155039	0.739	0.56	0.0013053	B cells	HNRNPA1
8.58E-08	0.46314818	0.472	0.278	0.0014129	B cells	ERP29
1.27E-07	0.44753428	0.394	0.223	0.0020892	B cells	SYPL1
1.62E-07	0.34188675	0.782	0.604	0.0026654	B cells	RPS17

2.44E-07	0.43947303	0.359	0.195	0.0040234	B cells	YWHAZ
3.58E-07	0.26092985	0.944	0.891	0.0059033	B cells	RPL7A
5.46E-07	0.42570104	0.528	0.357	0.0090011	B cells	ARPC2
6.69E-07	0.53662828	0.261	0.127	0.0110233	B cells	KLF6
2.44E-06	0.37389534	0.31	0.166	0.040252	B cells	PAPOLA
4.95E-06	0.33987734	0.324	0.179	0.0815127	B cells	HNRNPA0
1.26E-05	0.34781491	0.345	0.2	0.208269	B cells	SELENOT
1.64E-05	0.29547717	0.718	0.559	0.2696785	B cells	EIF3E
1.98E-05	0.34619121	0.331	0.194	0.3256582	B cells	ARPC5
2.34E-05	0.31954081	0.268	0.146	0.385127	B cells	RBMX
2.72E-05	0.38009618	0.359	0.222	0.4472854	B cells	TAGLN2
3.58E-05	0.34888183	0.296	0.17	0.5888897	B cells	HNRNPU
4.82E-05	0.3305246	0.394	0.256	0.7944689	B cells	SRSF5
5.49E-05	0.40889733	0.451	0.312	0.9047921	B cells	PNRC1
6.17E-05	0.25289247	0.775	0.63	1	B cells	EEF2
7.73E-05	0.3847728	0.352	0.223	1	B cells	LSM7
8.99E-05	0.31988886	0.324	0.198	1	B cells	RBM3
0.000124	0.29807915	0.282	0.165	1	B cells	ARPC4
0.000138	0.27658944	0.451	0.304	1	B cells	SERP1
0.000161	0.29665314	0.289	0.172	1	B cells	ZFP36L1
0.000171	0.32840816	0.401	0.273	1	B cells	SSR2
0.00082	0.26448397	0.352	0.238	1	B cells	TMBIM4
0.001632	0.27195862	0.352	0.244	1	B cells	SON
0.002447	0.25327158	0.289	0.193	1	B cells	HNRNPA3
0.007442	0.26069892	0.415	0.319	1	B cells	SET
0.007545	0.25941887	0.289	0.203	1	B cells	ARF6
0	2.92694431	0.911	0.001	0	Collecting duct intercalated cells	ATP6V1G3
0	2.29424914	0.785	0.007	0	Collecting duct intercalated cells	NUPR2
0	1.96012123	0.544	0.001	0	Collecting duct intercalated cells	ATP6V0D2
0	1.72461298	0.519	0.009	0	Collecting duct intercalated cells	ERP27
0	1.5116789	0.481	0	0	Collecting duct intercalated cells	DMRT2
0	1.36232286	0.418	0.003	0	Collecting duct intercalated cells	TMEM213
0	1.35854871	0.278	0.001	0	Collecting duct intercalated cells	SPINK1
0	1.35581578	0.494	0	0	Collecting duct intercalated cells	SRARP
0	1.27790754	0.342	0.001	0	Collecting duct intercalated cells	ADTRP
0	1.15063709	0.329	0.003	0	Collecting duct intercalated cells	BACE2
0	1.10045168	0.329	0.003	0	Collecting duct intercalated cells	FAM24B
0	1.03968353	0.316	0.003	0	Collecting duct intercalated cells	RCAN2
0	1.030599	0.266	0	0	Collecting duct intercalated cells	10-Mar
2.92E-273	0.91116963	0.266	0.003	4.81E-269	Collecting duct intercalated cells	SMIM5
1.78E-230	1.71433192	0.633	0.027	2.93E-226	Collecting duct intercalated cells	SMIM6
2.76E-223	1.05852043	0.316	0.006	4.55E-219	Collecting duct intercalated cells	HOXB-AS3
1.15E-179	1.16134896	0.38	0.012	1.90E-175	Collecting duct intercalated cells	MAL
2.63E-154	1.0800009	0.329	0.011	4.34E-150	Collecting duct intercalated cells	PTGER3
6.53E-142	1.12593412	0.253	0.007	1.08E-137	Collecting duct intercalated cells	RGCC
1.88E-140	0.64261712	0.266	0.007	3.10E-136	Collecting duct intercalated cells	FXYD4
5.12E-97	2.32043303	0.759	0.101	8.43E-93	Collecting duct intercalated cells	LGALS3
8.12E-93	1.58357839	0.43	0.031	1.34E-88	Collecting duct intercalated cells	OXCT1
2.01E-89	1.03591662	0.291	0.014	3.31E-85	Collecting duct intercalated cells	DMKN
1.19E-84	1.0789709	0.329	0.02	1.97E-80	Collecting duct intercalated cells	MARCKSL1
2.37E-83	1.32465629	0.405	0.03	3.90E-79	Collecting duct intercalated cells	IL18
1.22E-81	2.41038039	0.823	0.14	2.01E-77	Collecting duct intercalated cells	DEFB1
3.08E-68	2.06697922	0.722	0.125	5.08E-64	Collecting duct intercalated cells	CKB
1.34E-64	1.07975267	0.418	0.04	2.21E-60	Collecting duct intercalated cells	ARHGAP18
5.40E-61	1.35140473	0.873	0.183	8.90E-57	Collecting duct intercalated cells	S100A6
1.70E-52	1.09312739	0.43	0.052	2.80E-48	Collecting duct intercalated cells	GLTP
2.42E-50	1.74690587	0.671	0.143	3.99E-46	Collecting duct intercalated cells	LITAF
4.30E-45	1.85173235	1	0.486	7.08E-41	Collecting duct intercalated cells	C12orf75

1.28E-43	0.93695916	0.304	0.032	2.11E-39	Collecting duct intercalated cells	PPP2R5A
1.39E-43	1.34644942	0.987	0.391	2.29E-39	Collecting duct intercalated cells	TMSB4X
1.13E-35	1.51032307	0.62	0.158	1.86E-31	Collecting duct intercalated cells	COX7A1
1.91E-35	0.77588158	0.266	0.029	3.15E-31	Collecting duct intercalated cells	PRKAG2
4.65E-33	1.21771684	0.987	0.733	7.66E-29	Collecting duct intercalated cells	PPDPF
1.62E-28	0.99387576	0.987	0.867	2.67E-24	Collecting duct intercalated cells	RPS2
7.09E-28	1.16549724	0.646	0.201	1.17E-23	Collecting duct intercalated cells	PFN2
1.76E-27	1.16335269	0.747	0.283	2.90E-23	Collecting duct intercalated cells	HIGD1A
1.49E-25	0.79941509	1	0.949	2.45E-21	Collecting duct intercalated cells	RPL37A
3.14E-25	0.95244342	0.342	0.064	5.17E-21	Collecting duct intercalated cells	DUSP15
7.18E-25	0.65666776	0.266	0.039	1.18E-20	Collecting duct intercalated cells	RAB25
1.25E-24	0.66259908	1	0.997	2.06E-20	Collecting duct intercalated cells	RPL41
1.86E-24	1.04102838	0.329	0.061	3.06E-20	Collecting duct intercalated cells	EXOSC7
2.15E-24	0.77350305	0.975	0.698	3.54E-20	Collecting duct intercalated cells	CHCHD10
3.55E-24	0.65843439	1	0.952	5.85E-20	Collecting duct intercalated cells	RPL8
4.86E-24	1.081258	0.924	0.639	8.01E-20	Collecting duct intercalated cells	CALM2
4.90E-24	0.64101498	1	0.937	8.08E-20	Collecting duct intercalated cells	RPS24
5.04E-24	0.92374735	0.975	0.671	8.31E-20	Collecting duct intercalated cells	CYSTM1
5.71E-24	0.77374686	1	0.956	9.40E-20	Collecting duct intercalated cells	RPS8
6.37E-24	0.75231566	1	0.959	1.05E-19	Collecting duct intercalated cells	RPL31
9.27E-24	0.57717731	1	0.984	1.53E-19	Collecting duct intercalated cells	RPS6
2.07E-23	0.63824678	1	0.973	3.41E-19	Collecting duct intercalated cells	RPS12
2.93E-23	0.74466234	0.975	0.942	4.82E-19	Collecting duct intercalated cells	RPL15
3.53E-23	0.64587962	1	0.923	5.81E-19	Collecting duct intercalated cells	EIF1
4.43E-23	0.85423024	1	0.956	7.29E-19	Collecting duct intercalated cells	RPL21
2.43E-21	0.73097078	1	0.931	4.00E-17	Collecting duct intercalated cells	RPL6
1.12E-20	0.79197741	0.266	0.047	1.85E-16	Collecting duct intercalated cells	HPRT1
4.59E-20	0.53280725	0.987	0.976	7.56E-16	Collecting duct intercalated cells	RPL11
5.43E-20	0.54724087	1	0.978	8.95E-16	Collecting duct intercalated cells	RPS14
8.59E-20	0.74228356	1	0.971	1.41E-15	Collecting duct intercalated cells	RPS27
1.09E-19	1.16385435	0.57	0.213	1.79E-15	Collecting duct intercalated cells	IDH2
1.72E-19	0.55838281	1	0.972	2.84E-15	Collecting duct intercalated cells	RPL3
4.38E-19	0.91388359	0.405	0.102	7.22E-15	Collecting duct intercalated cells	IFITM2
5.70E-19	0.53060633	1	0.981	9.39E-15	Collecting duct intercalated cells	RPS18
9.30E-19	0.58885615	1	0.943	1.53E-14	Collecting duct intercalated cells	ATP5F1E
3.76E-18	0.67502865	1	0.942	6.19E-14	Collecting duct intercalated cells	RPS4X
4.85E-18	1.16889039	0.633	0.286	7.99E-14	Collecting duct intercalated cells	SLC25A39
6.38E-18	0.92363155	0.253	0.049	1.05E-13	Collecting duct intercalated cells	PHLDA1
1.91E-17	0.70493841	1	0.937	3.15E-13	Collecting duct intercalated cells	RPL36
2.74E-17	0.81812669	0.873	0.582	4.51E-13	Collecting duct intercalated cells	COX5A
1.17E-16	1.00775317	0.582	0.242	1.92E-12	Collecting duct intercalated cells	C9orf16
4.00E-16	0.59404262	0.266	0.056	6.59E-12	Collecting duct intercalated cells	LRRFIP2
9.00E-16	0.47219773	1	0.987	1.48E-11	Collecting duct intercalated cells	RPL32
1.81E-15	0.5846252	0.949	0.879	2.97E-11	Collecting duct intercalated cells	ATP5MG
1.99E-15	0.45313622	0.987	0.963	3.27E-11	Collecting duct intercalated cells	RPS3A
3.37E-15	0.54061631	1	0.932	5.55E-11	Collecting duct intercalated cells	RPL27A
4.39E-15	0.78972321	0.937	0.719	7.22E-11	Collecting duct intercalated cells	SLC25A5
5.28E-15	0.51667469	1	0.904	8.69E-11	Collecting duct intercalated cells	NDUFA4
5.33E-15	0.81517137	0.848	0.637	8.77E-11	Collecting duct intercalated cells	ATP6V1F
7.59E-15	0.51598894	0.987	0.954	1.25E-10	Collecting duct intercalated cells	RPL35A
1.13E-14	0.68438867	0.924	0.741	1.87E-10	Collecting duct intercalated cells	UQCR11
1.26E-14	0.79278399	0.506	0.186	2.07E-10	Collecting duct intercalated cells	SLC25A4
1.65E-14	0.51669624	0.987	0.938	2.71E-10	Collecting duct intercalated cells	RPL23A
1.71E-14	0.62509342	0.949	0.815	2.82E-10	Collecting duct intercalated cells	COX7A2
1.71E-14	0.72578032	0.532	0.198	2.82E-10	Collecting duct intercalated cells	RBM3
2.22E-14	0.92290231	0.671	0.352	3.66E-10	Collecting duct intercalated cells	ATP6V1E1
3.14E-14	1.02429689	0.734	0.396	5.17E-10	Collecting duct intercalated cells	CD24
3.33E-14	0.54355234	0.987	0.959	5.48E-10	Collecting duct intercalated cells	RPS15A

5.61E-14	0.45218549	1	0.947	9.25E-10	Collecting duct intercalated cells	RPL35
9.45E-14	0.46001016	1	0.994	1.56E-09	Collecting duct intercalated cells	RPL34
1.25E-13	0.59424424	0.949	0.78	2.05E-09	Collecting duct intercalated cells	COX6C
2.23E-13	0.49429704	1	0.97	3.67E-09	Collecting duct intercalated cells	RPL7
2.35E-13	0.64738691	0.962	0.785	3.87E-09	Collecting duct intercalated cells	UQCR10
2.84E-13	0.74863527	0.772	0.494	4.68E-09	Collecting duct intercalated cells	NDUFA5
4.26E-13	0.54584019	0.962	0.887	7.01E-09	Collecting duct intercalated cells	RPL10A
4.48E-13	0.50892513	0.987	0.943	7.38E-09	Collecting duct intercalated cells	RPS13
7.54E-13	0.6065536	1	0.858	1.24E-08	Collecting duct intercalated cells	RPL39
9.48E-13	0.48503628	0.962	0.851	1.56E-08	Collecting duct intercalated cells	RPS3
1.50E-12	0.72964321	0.861	0.538	2.48E-08	Collecting duct intercalated cells	S100A11
2.38E-12	0.73249548	0.519	0.215	3.92E-08	Collecting duct intercalated cells	SVIP
2.71E-12	0.67638302	0.43	0.156	4.46E-08	Collecting duct intercalated cells	DYNLT3
3.09E-12	0.60233088	0.899	0.718	5.09E-08	Collecting duct intercalated cells	SLC25A6
4.38E-12	0.50082473	0.911	0.761	7.22E-08	Collecting duct intercalated cells	COX8A
4.73E-12	0.54353956	0.253	0.064	7.80E-08	Collecting duct intercalated cells	FGF7
5.77E-12	0.33926566	1	0.99	9.51E-08	Collecting duct intercalated cells	RPL13A
7.34E-12	0.63534027	0.342	0.107	1.21E-07	Collecting duct intercalated cells	SPINT1-AS1
7.81E-12	0.62482894	0.899	0.675	1.29E-07	Collecting duct intercalated cells	ISCU
8.30E-12	0.61248836	0.253	0.066	1.37E-07	Collecting duct intercalated cells	MSI2
1.30E-11	0.55883233	1	0.964	2.15E-07	Collecting duct intercalated cells	RPL12
1.46E-11	0.46499089	0.987	0.965	2.41E-07	Collecting duct intercalated cells	RPL26
1.86E-11	0.79626639	0.532	0.245	3.07E-07	Collecting duct intercalated cells	NDUFA8
2.07E-11	0.53031128	0.924	0.775	3.41E-07	Collecting duct intercalated cells	NDUFA1
2.15E-11	0.71098571	0.456	0.189	3.54E-07	Collecting duct intercalated cells	ETFRF1
2.18E-11	0.54948422	0.899	0.75	3.59E-07	Collecting duct intercalated cells	TMA7
2.67E-11	0.77546009	0.38	0.134	4.39E-07	Collecting duct intercalated cells	MAP1LC3A
5.57E-11	0.36605234	1	0.963	9.17E-07	Collecting duct intercalated cells	RPS25
5.92E-11	0.58192454	0.886	0.64	9.76E-07	Collecting duct intercalated cells	DYNLL1
6.97E-11	0.45495298	0.975	0.891	1.15E-06	Collecting duct intercalated cells	RPL7A
9.87E-11	0.91412108	0.696	0.484	1.63E-06	Collecting duct intercalated cells	CYCS
1.62E-10	0.64854797	0.494	0.219	2.68E-06	Collecting duct intercalated cells	TSPO
1.89E-10	0.76223752	0.734	0.479	3.12E-06	Collecting duct intercalated cells	RPL17
2.36E-10	0.40050773	1	0.935	3.88E-06	Collecting duct intercalated cells	RPL14
2.37E-10	0.8087085	0.658	0.384	3.91E-06	Collecting duct intercalated cells	MPC1
2.45E-10	0.60801539	0.519	0.226	4.03E-06	Collecting duct intercalated cells	VAMP2
2.86E-10	0.42908665	1	0.909	4.71E-06	Collecting duct intercalated cells	RPL24
3.14E-10	0.41341725	1	0.924	5.16E-06	Collecting duct intercalated cells	RPS20
3.27E-10	0.4656602	0.899	0.729	5.38E-06	Collecting duct intercalated cells	OST4
5.13E-10	0.70605726	0.696	0.404	8.45E-06	Collecting duct intercalated cells	S100A10
5.61E-10	0.44742261	1	0.937	9.24E-06	Collecting duct intercalated cells	RPL9
5.62E-10	0.36671873	1	0.983	9.26E-06	Collecting duct intercalated cells	RPS15
7.22E-10	0.70897903	0.494	0.224	1.19E-05	Collecting duct intercalated cells	FAM200B
7.42E-10	0.59880391	0.481	0.211	1.22E-05	Collecting duct intercalated cells	SRI
9.58E-10	0.48955114	0.911	0.765	1.58E-05	Collecting duct intercalated cells	RPL4
1.08E-09	0.58898282	0.861	0.609	1.77E-05	Collecting duct intercalated cells	COMMD6
1.27E-09	0.73246222	0.671	0.423	2.10E-05	Collecting duct intercalated cells	ACTG1
1.73E-09	0.35216398	0.987	0.911	2.85E-05	Collecting duct intercalated cells	COX4I1
2.02E-09	0.52938285	0.38	0.144	3.33E-05	Collecting duct intercalated cells	VBP1
2.36E-09	0.65433904	0.684	0.422	3.89E-05	Collecting duct intercalated cells	COX7A2L
2.79E-09	0.60411734	0.797	0.559	4.59E-05	Collecting duct intercalated cells	MINOS1
2.89E-09	0.5191592	0.886	0.705	4.76E-05	Collecting duct intercalated cells	RPSA
2.90E-09	0.37715141	0.975	0.971	4.77E-05	Collecting duct intercalated cells	COX7C
3.10E-09	0.5093426	0.861	0.68	5.11E-05	Collecting duct intercalated cells	ATP5PO
3.40E-09	0.47563045	0.937	0.754	5.61E-05	Collecting duct intercalated cells	CHCHD2
3.44E-09	0.3925439	0.987	0.926	5.67E-05	Collecting duct intercalated cells	RPL28
3.45E-09	0.66249252	0.582	0.321	5.69E-05	Collecting duct intercalated cells	NDUFA12
3.72E-09	0.44847906	0.949	0.87	6.13E-05	Collecting duct intercalated cells	RPL5

8.23E-09	0.28070095	0.975	0.922	0.0001355	Collecting duct intercalated cells	RPS19
8.91E-09	0.64909332	0.57	0.315	0.0001468	Collecting duct intercalated cells	ATP6V1D
1.22E-08	0.54938879	0.747	0.507	0.0002003	Collecting duct intercalated cells	YWHAE
1.22E-08	0.6905924	0.266	0.089	0.0002008	Collecting duct intercalated cells	TBC1D1
1.30E-08	0.54979544	0.823	0.526	0.0002142	Collecting duct intercalated cells	RARRES3
1.49E-08	0.48655777	0.873	0.73	0.0002455	Collecting duct intercalated cells	TOMM7
1.83E-08	0.56670747	0.557	0.289	0.0003016	Collecting duct intercalated cells	TPM1
1.86E-08	0.56690498	0.81	0.604	0.0003063	Collecting duct intercalated cells	RPS17
1.97E-08	0.42846165	0.937	0.882	0.0003248	Collecting duct intercalated cells	RPL22
2.34E-08	0.53290141	0.709	0.448	0.0003862	Collecting duct intercalated cells	ANAPC16
2.67E-08	0.59079312	0.392	0.171	0.0004399	Collecting duct intercalated cells	OTUD6B-AS1
2.85E-08	0.57396009	0.658	0.405	0.00047	Collecting duct intercalated cells	CSTB
2.99E-08	0.5732641	0.481	0.236	0.0004917	Collecting duct intercalated cells	MIEN1
3.96E-08	0.41974102	0.962	0.913	0.0006519	Collecting duct intercalated cells	RPL37
4.02E-08	0.57796266	0.329	0.127	0.0006623	Collecting duct intercalated cells	EPS8
4.40E-08	0.27852558	1	0.989	0.000725	Collecting duct intercalated cells	RPL13
4.47E-08	0.61942316	0.304	0.115	0.000737	Collecting duct intercalated cells	ZFYVE21
6.40E-08	0.41627612	0.962	0.837	0.0010545	Collecting duct intercalated cells	FXYD2
6.54E-08	0.72786496	0.481	0.245	0.0010779	Collecting duct intercalated cells	BOLA3
7.93E-08	0.49428496	0.937	0.816	0.0013053	Collecting duct intercalated cells	UQCRB
8.26E-08	0.44637121	0.937	0.869	0.0013611	Collecting duct intercalated cells	RPL38
1.02E-07	0.4391931	0.962	0.895	0.0016773	Collecting duct intercalated cells	RPS29
1.12E-07	0.3609416	0.975	0.879	0.0018505	Collecting duct intercalated cells	RPL30
1.15E-07	0.28343746	1	0.996	0.0018994	Collecting duct intercalated cells	EEF1A1
1.16E-07	0.48247253	0.494	0.238	0.0019051	Collecting duct intercalated cells	ZCRB1
1.37E-07	0.37919968	0.975	0.932	0.0022592	Collecting duct intercalated cells	RPS16
1.49E-07	0.59413321	0.329	0.135	0.0024496	Collecting duct intercalated cells	PNRC2
1.50E-07	0.52837865	0.797	0.561	0.0024783	Collecting duct intercalated cells	HNRNPA1
1.76E-07	0.33538098	0.975	0.9	0.0029019	Collecting duct intercalated cells	UBA52
2.42E-07	0.45763143	0.886	0.773	0.0039874	Collecting duct intercalated cells	COX7B
2.54E-07	0.5695723	0.747	0.509	0.0041835	Collecting duct intercalated cells	COA3
2.70E-07	0.53550915	0.468	0.237	0.0044416	Collecting duct intercalated cells	GABARAPL1
3.14E-07	0.4065513	0.266	0.095	0.0051664	Collecting duct intercalated cells	ZFAND1
3.52E-07	0.48381493	0.43	0.202	0.0058053	Collecting duct intercalated cells	PCNP
4.08E-07	0.79210771	0.418	0.213	0.0067119	Collecting duct intercalated cells	CHP1
4.70E-07	0.3972881	0.949	0.871	0.0077436	Collecting duct intercalated cells	RPL27
5.57E-07	0.45042878	0.797	0.605	0.0091754	Collecting duct intercalated cells	SLIRP
5.67E-07	0.52865626	0.316	0.129	0.0093431	Collecting duct intercalated cells	EFHD1
5.89E-07	0.71422811	0.481	0.281	0.0096958	Collecting duct intercalated cells	YWHAQ
6.50E-07	0.44076209	0.266	0.1	0.0107048	Collecting duct intercalated cells	FAM229B
7.10E-07	0.36611484	0.975	0.854	0.0116906	Collecting duct intercalated cells	SRP14
7.16E-07	0.36058529	0.911	0.845	0.0117999	Collecting duct intercalated cells	COX6B1
7.68E-07	0.49889348	0.709	0.526	0.0126533	Collecting duct intercalated cells	COX17
9.00E-07	0.45908636	0.342	0.148	0.0148209	Collecting duct intercalated cells	TPD52
9.16E-07	0.55657879	0.671	0.469	0.0150862	Collecting duct intercalated cells	RPL36A
1.04E-06	0.59440652	0.354	0.159	0.0171685	Collecting duct intercalated cells	HAX1
1.13E-06	0.57536832	0.304	0.127	0.0185303	Collecting duct intercalated cells	NIPSNAP2
1.55E-06	0.27558989	0.937	0.879	0.0255242	Collecting duct intercalated cells	RPS5
1.65E-06	0.52604679	0.519	0.306	0.0271727	Collecting duct intercalated cells	VDAC2
1.74E-06	0.51689495	0.468	0.25	0.0286777	Collecting duct intercalated cells	TUBA1B
1.87E-06	0.47891174	0.62	0.402	0.030772	Collecting duct intercalated cells	SNU13
2.51E-06	0.50206034	0.734	0.484	0.0413553	Collecting duct intercalated cells	RPS4Y1
2.66E-06	0.31140601	0.962	0.872	0.0437724	Collecting duct intercalated cells	RACK1
3.07E-06	0.53538545	0.329	0.149	0.0505636	Collecting duct intercalated cells	YWHAH
3.74E-06	0.46498253	0.38	0.183	0.0616699	Collecting duct intercalated cells	MRPS6
3.79E-06	0.55315103	0.506	0.293	0.0624845	Collecting duct intercalated cells	RHEB
3.80E-06	0.581869	0.354	0.171	0.0625491	Collecting duct intercalated cells	LINC00671
4.17E-06	0.49785376	0.367	0.178	0.068695	Collecting duct intercalated cells	GNAS

4.34E-06	0.35666986	0.911	0.792	0.0715222	Collecting duct intercalated cells	ATP5MPL
4.49E-06	0.37763132	0.81	0.739	0.0739274	Collecting duct intercalated cells	UQCRH
4.52E-06	0.55438086	0.342	0.166	0.0745201	Collecting duct intercalated cells	PDHB
4.55E-06	0.49361792	0.519	0.303	0.0750253	Collecting duct intercalated cells	NHP2
4.68E-06	0.59183819	0.494	0.29	0.0771574	Collecting duct intercalated cells	HADHB
4.70E-06	0.50370577	0.38	0.186	0.0773683	Collecting duct intercalated cells	DLD
5.05E-06	0.42867338	0.81	0.6	0.0831273	Collecting duct intercalated cells	RPS10
5.78E-06	0.3198976	0.975	0.886	0.0952151	Collecting duct intercalated cells	NACA
5.82E-06	0.67404087	0.532	0.333	0.0959084	Collecting duct intercalated cells	SELENOW
7.42E-06	0.27360206	0.911	0.879	0.1221902	Collecting duct intercalated cells	COX5B
7.55E-06	0.29671131	0.987	0.935	0.1243188	Collecting duct intercalated cells	RPL18A
7.87E-06	0.48187886	0.57	0.35	0.1295965	Collecting duct intercalated cells	MRPL33
7.94E-06	0.38320448	0.924	0.777	0.1307585	Collecting duct intercalated cells	COX6A1
7.98E-06	0.42317171	0.253	0.102	0.1314032	Collecting duct intercalated cells	SNRNP25
8.16E-06	0.37608734	0.304	0.133	0.1343872	Collecting duct intercalated cells	TRAPPC3
8.31E-06	0.53118085	0.557	0.361	0.1369429	Collecting duct intercalated cells	VAPA
8.34E-06	0.38651133	0.418	0.213	0.1373256	Collecting duct intercalated cells	BTG1
8.62E-06	0.4132898	0.304	0.135	0.141959	Collecting duct intercalated cells	MRPL16
1.04E-05	0.3466238	0.975	0.854	0.1710745	Collecting duct intercalated cells	RPL23
1.18E-05	0.37446852	0.924	0.821	0.1948483	Collecting duct intercalated cells	RPLP0
1.71E-05	0.46318	0.671	0.482	0.2811814	Collecting duct intercalated cells	HMGN1
1.78E-05	0.49471679	0.392	0.212	0.2932363	Collecting duct intercalated cells	COPRS
1.89E-05	0.41284059	0.873	0.776	0.3112997	Collecting duct intercalated cells	NDUFS5
1.92E-05	0.52788313	0.519	0.333	0.3156949	Collecting duct intercalated cells	ANAPC13
1.96E-05	0.4446399	0.266	0.116	0.3234318	Collecting duct intercalated cells	WASHC3
2.28E-05	0.25011712	1	0.979	0.3763242	Collecting duct intercalated cells	RPS23
2.35E-05	0.4248976	0.696	0.546	0.3869593	Collecting duct intercalated cells	NDUFB3
2.40E-05	0.53112155	0.646	0.46	0.3960895	Collecting duct intercalated cells	NDUFA6
2.49E-05	0.30946991	0.962	0.905	0.4100277	Collecting duct intercalated cells	FAU
2.50E-05	0.41164046	0.785	0.624	0.4113871	Collecting duct intercalated cells	EIF3K
2.82E-05	0.27006402	0.975	0.98	0.4641683	Collecting duct intercalated cells	RPS27A
2.96E-05	0.49855749	0.557	0.386	0.4867371	Collecting duct intercalated cells	CYC1
2.99E-05	0.38491818	0.658	0.448	0.4930185	Collecting duct intercalated cells	HSBP1
3.34E-05	0.30890643	0.861	0.732	0.5503095	Collecting duct intercalated cells	PRDX5
3.36E-05	0.49688727	0.304	0.149	0.5531143	Collecting duct intercalated cells	GLO1
3.43E-05	0.47158562	0.57	0.392	0.5642902	Collecting duct intercalated cells	UBE2D3
3.56E-05	0.45418378	0.316	0.153	0.585695	Collecting duct intercalated cells	SUMO3
3.70E-05	0.47110115	0.43	0.249	0.6095081	Collecting duct intercalated cells	DMAC1
3.93E-05	0.57317088	0.633	0.5	0.6470325	Collecting duct intercalated cells	EIF4A2
4.49E-05	0.36464908	0.595	0.376	0.7401784	Collecting duct intercalated cells	SDHD
4.50E-05	0.60334413	0.43	0.259	0.7405029	Collecting duct intercalated cells	C6orf48
4.61E-05	0.53370457	0.608	0.446	0.7591751	Collecting duct intercalated cells	VDAC1
4.63E-05	0.37645057	0.582	0.392	0.7621333	Collecting duct intercalated cells	NDUFB5
5.12E-05	0.41230152	0.532	0.339	0.8438586	Collecting duct intercalated cells	CALM3
5.76E-05	0.41845689	0.646	0.446	0.9485636	Collecting duct intercalated cells	RTN4
6.22E-05	0.37413266	0.329	0.165	1	Collecting duct intercalated cells	PAIP1
7.12E-05	0.49383222	0.253	0.118	1	Collecting duct intercalated cells	APIP
7.54E-05	0.30541173	0.848	0.75	1	Collecting duct intercalated cells	SKP1
8.22E-05	0.44575925	0.481	0.298	1	Collecting duct intercalated cells	EIF3F
8.89E-05	0.45154618	0.392	0.221	1	Collecting duct intercalated cells	DNAJC19
0.000104	0.39843626	0.456	0.254	1	Collecting duct intercalated cells	MCCD1
0.000107	0.3621809	0.633	0.45	1	Collecting duct intercalated cells	C19orf70
0.000111	0.50635265	0.494	0.317	1	Collecting duct intercalated cells	EIF3L
0.000114	0.39761823	0.354	0.189	1	Collecting duct intercalated cells	AK3
0.000114	0.26686665	0.873	0.786	1	Collecting duct intercalated cells	ATP5PF
0.000117	0.46770254	0.304	0.155	1	Collecting duct intercalated cells	SELENOM
0.000126	0.55775966	0.392	0.235	1	Collecting duct intercalated cells	PKM
0.000128	0.46090388	0.532	0.347	1	Collecting duct intercalated cells	UFC1

0.000134	0.4822629	0.329	0.176	1	Collecting duct intercalated cells	GTF2A2
0.000143	0.46173442	0.342	0.182	1	Collecting duct intercalated cells	ANXA11
0.000173	0.34802008	0.481	0.297	1	Collecting duct intercalated cells	RTRAF
0.000184	0.4208666	0.582	0.395	1	Collecting duct intercalated cells	IFITM3
0.000199	0.44852253	0.481	0.309	1	Collecting duct intercalated cells	TAX1BP1
0.000204	0.45477485	0.304	0.156	1	Collecting duct intercalated cells	IAH1
0.000208	0.38790125	0.456	0.287	1	Collecting duct intercalated cells	SCOC
0.00022	0.36774833	0.278	0.138	1	Collecting duct intercalated cells	PMPCB
0.000222	0.47001832	0.468	0.301	1	Collecting duct intercalated cells	PNKD
0.000297	0.41045014	0.557	0.388	1	Collecting duct intercalated cells	CNN3
0.0003	0.39923413	0.253	0.124	1	Collecting duct intercalated cells	MTX2
0.000311	0.32560308	0.861	0.771	1	Collecting duct intercalated cells	UBL5
0.000317	0.51299728	0.38	0.234	1	Collecting duct intercalated cells	ATP6V0B
0.000321	0.39734715	0.722	0.559	1	Collecting duct intercalated cells	EIF3E
0.00033	0.29963938	0.278	0.136	1	Collecting duct intercalated cells	FAM45A
0.000365	0.38935997	0.266	0.135	1	Collecting duct intercalated cells	ISCA1
0.00042	0.44928561	0.266	0.134	1	Collecting duct intercalated cells	ANAPC15
0.000437	0.31953592	0.266	0.133	1	Collecting duct intercalated cells	RAB11B
0.000447	0.44479797	0.595	0.472	1	Collecting duct intercalated cells	CCNI
0.000448	0.27123328	0.962	0.899	1	Collecting duct intercalated cells	PTMA
0.000465	0.2710943	0.835	0.698	1	Collecting duct intercalated cells	NDUFC1
0.000476	0.26961162	0.962	0.821	1	Collecting duct intercalated cells	RPL18
0.000522	0.35422675	0.62	0.438	1	Collecting duct intercalated cells	SRP9
0.000579	0.43243898	0.62	0.456	1	Collecting duct intercalated cells	SELENOH
0.000585	0.28017754	0.772	0.642	1	Collecting duct intercalated cells	PRR13
0.000599	0.34752285	0.759	0.59	1	Collecting duct intercalated cells	SUMO2
0.000663	0.40264408	0.278	0.146	1	Collecting duct intercalated cells	ARPP19
0.000674	0.45222403	0.506	0.351	1	Collecting duct intercalated cells	POLR2K
0.0007	0.27894877	0.899	0.8	1	Collecting duct intercalated cells	MIF
0.000751	0.32207038	0.582	0.382	1	Collecting duct intercalated cells	CAMK2N1
0.000791	0.42338799	0.684	0.583	1	Collecting duct intercalated cells	ATP5F1B
0.000924	0.32728578	0.57	0.396	1	Collecting duct intercalated cells	EIF3H
0.000998	0.27253736	0.494	0.321	1	Collecting duct intercalated cells	CHMP5
0.001046	0.34761925	0.291	0.158	1	Collecting duct intercalated cells	YPEL5
0.001046	0.41124618	0.392	0.245	1	Collecting duct intercalated cells	MIR4458HG
0.001151	0.26501494	0.886	0.782	1	Collecting duct intercalated cells	SAT1
0.001156	0.32641727	0.291	0.161	1	Collecting duct intercalated cells	CCDC124
0.001159	0.27631122	0.253	0.128	1	Collecting duct intercalated cells	PTP4A1
0.001161	0.31308217	0.316	0.175	1	Collecting duct intercalated cells	CAPG
0.001419	0.40815596	0.266	0.147	1	Collecting duct intercalated cells	RBMX
0.00144	0.28562101	0.43	0.275	1	Collecting duct intercalated cells	SYF2
0.001864	0.37133925	0.658	0.521	1	Collecting duct intercalated cells	NDUFA13
0.00189	0.42598468	0.405	0.275	1	Collecting duct intercalated cells	RAB11A
0.001914	0.38261154	0.43	0.294	1	Collecting duct intercalated cells	TMEM14B
0.001937	0.36959519	0.342	0.211	1	Collecting duct intercalated cells	TBCB
0.002266	0.25265013	0.443	0.29	1	Collecting duct intercalated cells	UQCRC2
0.002276	0.31857416	0.684	0.583	1	Collecting duct intercalated cells	NDUFB8
0.002503	0.28161623	0.418	0.265	1	Collecting duct intercalated cells	MOCS2
0.002726	0.29638687	0.494	0.34	1	Collecting duct intercalated cells	TMEM230
0.002954	0.34876202	0.418	0.277	1	Collecting duct intercalated cells	CAPZB
0.002999	0.30256011	0.253	0.14	1	Collecting duct intercalated cells	PMF1
0.003071	0.3288932	0.253	0.14	1	Collecting duct intercalated cells	TATDN1
0.00319	0.28718928	0.709	0.582	1	Collecting duct intercalated cells	ATP5MC1
0.003414	0.26738117	0.253	0.139	1	Collecting duct intercalated cells	SUCLA2
0.003659	0.3969932	0.494	0.38	1	Collecting duct intercalated cells	ATP5PB
0.003684	0.31938647	0.342	0.212	1	Collecting duct intercalated cells	DNAJC8
0.003756	0.26816589	0.43	0.28	1	Collecting duct intercalated cells	NDUFA10
0.004094	0.32867276	0.392	0.26	1	Collecting duct intercalated cells	DUT

0.004123	0.27860778	0.861	0.756	1	Collecting duct intercalated cells	ATP5MF
0.004304	0.27005389	0.456	0.311	1	Collecting duct intercalated cells	EIF2S2
0.004319	0.32925875	0.519	0.376	1	Collecting duct intercalated cells	TMEM141
0.004417	0.39774831	0.316	0.206	1	Collecting duct intercalated cells	PTP4A2
0.005195	0.27255401	0.899	0.767	1	Collecting duct intercalated cells	OAZ1
0.005252	0.26910701	0.481	0.329	1	Collecting duct intercalated cells	MRPS33
0.005314	0.31870992	0.329	0.205	1	Collecting duct intercalated cells	MEAF6
0.00557	0.32053555	0.405	0.278	1	Collecting duct intercalated cells	SNRPF
0.005694	0.28547759	0.481	0.344	1	Collecting duct intercalated cells	C11orf58
0.00625	0.32005405	0.392	0.268	1	Collecting duct intercalated cells	TXNL4A
0.006561	0.31974775	0.57	0.45	1	Collecting duct intercalated cells	HIGD2A
0.006782	0.34043267	0.456	0.333	1	Collecting duct intercalated cells	PTGES3
0.006822	0.32046579	0.582	0.495	1	Collecting duct intercalated cells	HSP90AB1
0.006878	0.33702864	0.253	0.149	1	Collecting duct intercalated cells	RSRC2
0.007275	0.2501856	0.671	0.552	1	Collecting duct intercalated cells	PRDX6
0.007611	0.29814209	0.38	0.253	1	Collecting duct intercalated cells	NBDY
0.007829	0.3185557	0.608	0.518	1	Collecting duct intercalated cells	UQCRFS1
0.009535	0.27975278	0.722	0.636	1	Collecting duct intercalated cells	ATP5PD
0.009611	0.32031671	0.329	0.218	1	Collecting duct intercalated cells	TCEA1
0.009678	0.28307457	0.418	0.287	1	Collecting duct intercalated cells	SNRPN
